# Supplementary material for: Synthesis of Novel Lipophilic Polyamines via Ugi Reaction and Evaluation of Their Anticancer Activity
Source: Molecules. 2022 Sep 21;27(19):6218. doi: 10.3390/molecules27196218 (PMC9572921; doi:10.3390/molecules27196218)
Supplement: Supplementary file 1 [file molecules-27-06218-s001.zip › molecules-1888508-supplementary.pdf]

Supporting information

Synthesis of Novel Lipophilic Polyamines via Ugi Reaction and Evaluation of Their Anticancer Activity

Artemiy Nichugovskiy [1], Maksimova Varvara [2], Trapeznikova Ekaterina [3], Elizaveta Eshtukova-Shcheglova[1], Ivanov Igor [1], Yakubovskaya Marianna [2], Kirsanov Kirill [2,4], Cheshkov Dmitry [5], Gian Cesare Tron [6], Mikhail Maslov [1]

[1] Lomonosov Institute of Fine Chemical Technologies, MIREA—Russian Technological University, 86 Vernadsky Ave., 119571 Moscow, Russia

[2] N.N. Blokhin National Medical Research Center of Oncology, 23 Kashirskoe sh., 115478, Moscow, Russia

[3] I.M. Sechenov First Moscow State Medical University, 8-2 Trubetskaya Str., 119991 Moscow, Russia

[4] Peoples' Friendship University of Russia, 6 Miklukho-Maklaya Str., 117198 Moscow, Russia

[5] State Scientific Research Institute of Chemistry and Technology of Organoelement Compounds, 38 Shosse Entuziastov, 105118 Moscow, Russia

[6] Dipartimento di Scienza del Farmaco, Università del Piemonte Orientale, 2 Largo Donegani, 28100 Novara, Italy

1,8-diamino-*N*[8]-acetyl-*N*[1]-*tert*-butyl-1,7-dioxo-*N*[3],*N*[6]-dibenzyl-3,6-diazaoctane (**11a**)

Yield: 11%, colorless oil. Eluent: $CHCl_3$-MeOH (10:1). ^1H NMR (600 MHz, acetone-d6, main rotamer) δ 1.31 (s, 9H, $(CH_3)_3$), 1.93 (s, 3H, $COCH_3$), 2.62 (br. s, 2H, $CH_2NC\underline{H}_2$), 3.05 (s, 2H, $COCH_2N$), 3.50 (s, 2H, CH_2NCO), 3.60 (s, 2H, $COC\underline{H}_2NH$), 4.05 (d, 2H, *J* = 4.8 Hz, $PhCH_2$), 4.37 (s, 2H, $PhCH_2NCO$), 7.07 – 7.46 (m, 12H, 2 Ph, 2 NH). ^{13}C NMR (150 MHz, acetone-d6, main rotamer) δ 22.71, 28.96, 42.04, 44.42, 50.41, 52.11, 59.47, 59.95, 127.58, 128.34, 128.72, 129.35, 129.40, 129.67, 137.71, 138.67, 169.62, 170.21, 170.36. HRMS ESI m/z: $[M+H]^+$ calcd for $C_{26}H_{37}N_4O_3$ 453.2860, found: 453.2860.

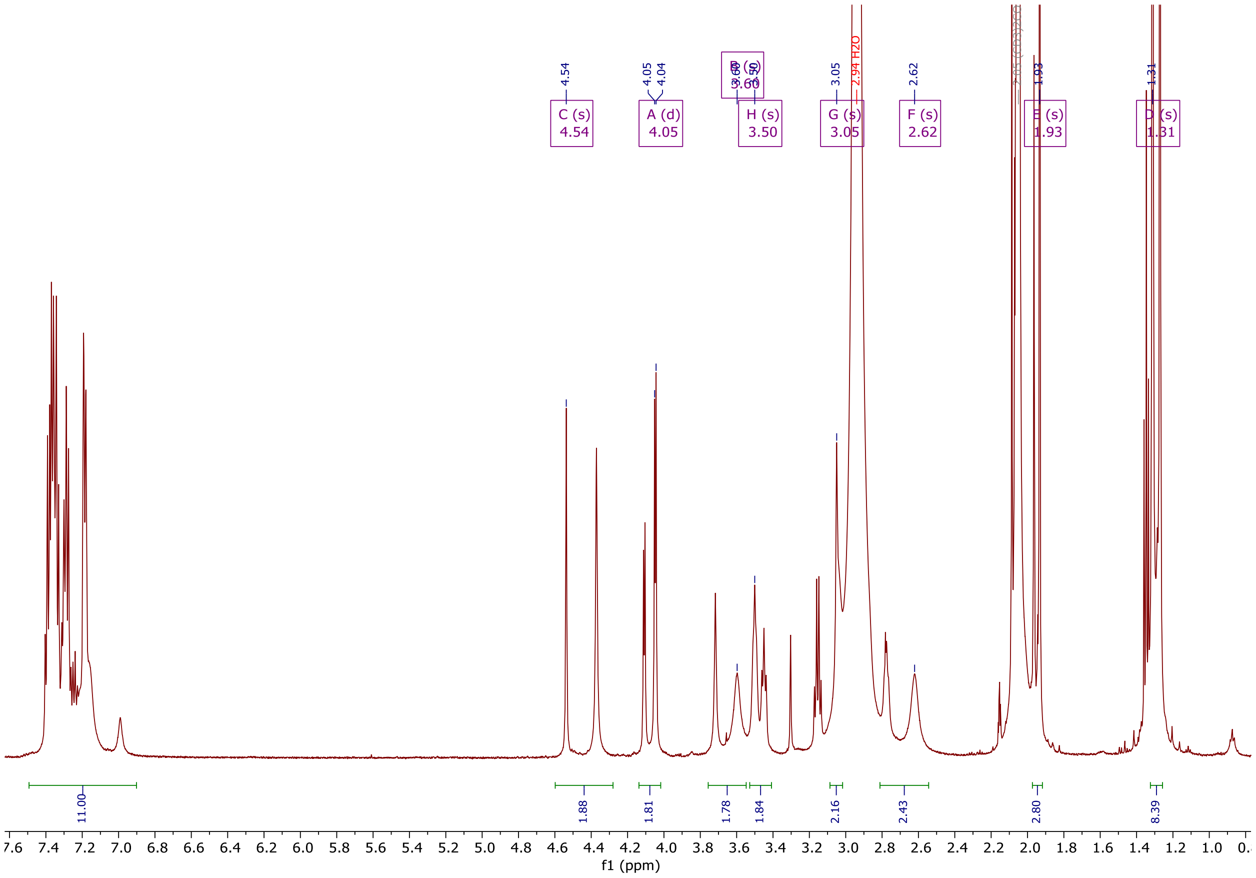

Figure S1. ^1H NMR spectrum of compound **11a.**

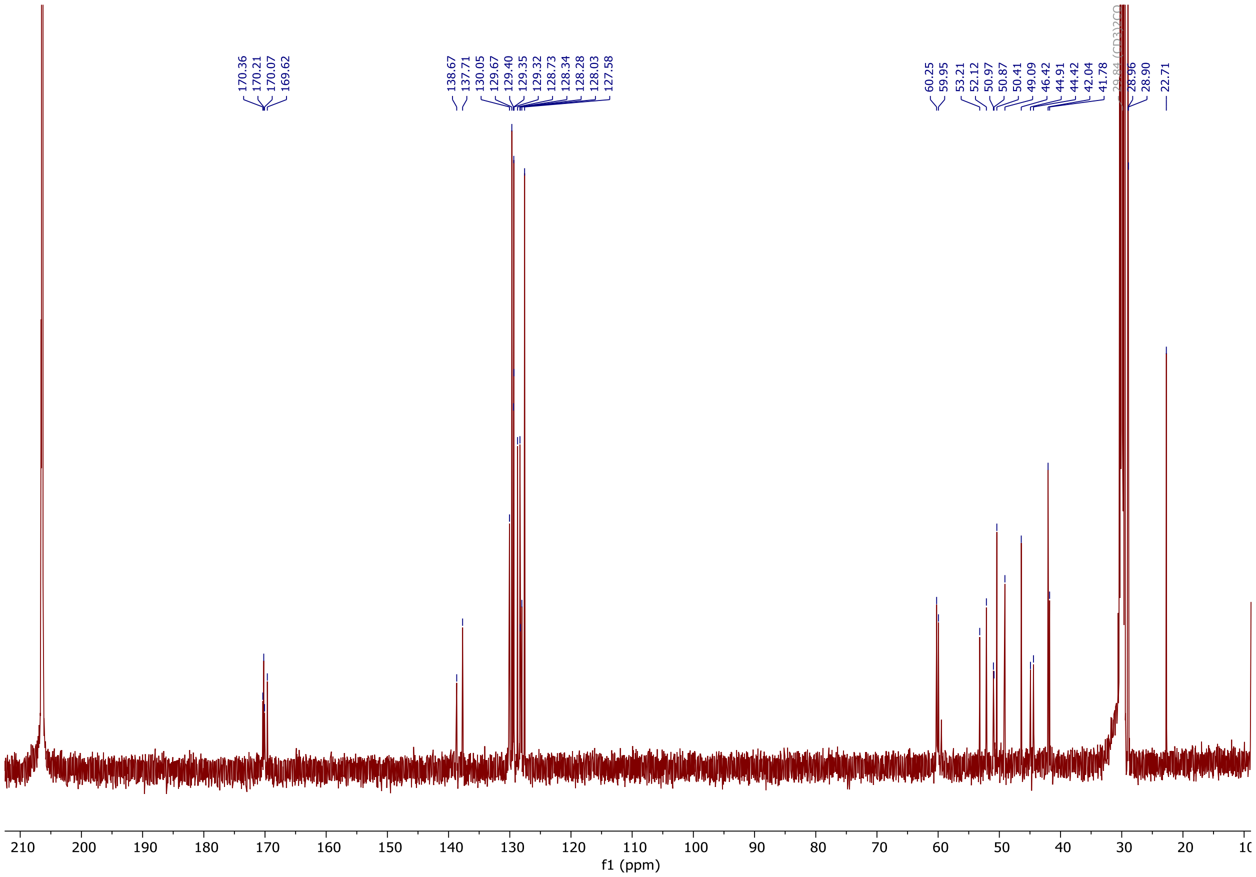

Figure S2. ^{13}C NMR spectrum of compound **11a.**

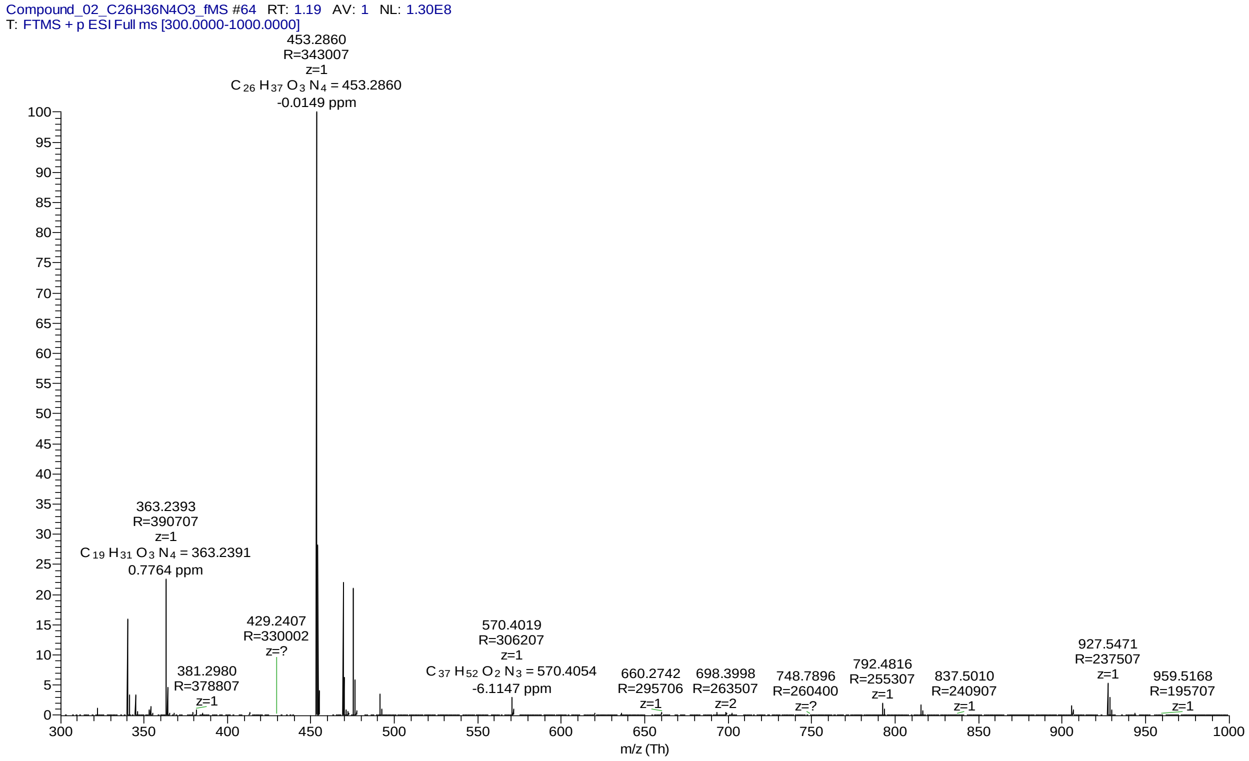

Figure S3. HRMS spectrum of compound **11a.**

1,8-diamino-*N*[8]-acetyl-*N*[1]-octadecyl-1,7-dioxo-*N*[3],*N*[6]-dibenzyl-3,6-diazaoctane (**11b**)

Yield: 10%, colorless oil. Eluent: $CHCl_3$-MeOH (10:1). ^1H NMR (300 MHz, $CDCl_3$, main rotamer) δ 0.88 (t, 3H, *J* = 7.0 Hz, $(CH_2)_{15}C\underline{H}_3$), 1.25 (br. s, 30H, $(C\underline{H}_2)_{15}CH_3$), 1.39 – 1.57 (m, 2H, $CH_2C\underline{H}_2(CH_2)_{15}$), 2.05 (s, 3H, $COCH_3$), 2.59 (t, 2H, *J* = 6.5 Hz, $COCH_2NC\underline{H}_2$), 3.16 (s, 2H, $COCH_2N$), 3.20 – 3.35 (m, 2H, $C\underline{H}_2CH_2(CH_2)_{15}$), 3.47 (t, 2H, *J* = 6.5 Hz, $CONC\underline{H}_2$), 3.57 (s, 2H, $COC\underline{H}_2NH$), 4.04 (s, 2H, $PhCH_2$), 4.14 (s, 2H, $PhCH_2NCO$), 6.51 (s, 1H, $N\underline{H}COCH_2$), 6.97 – 7.50 (m, 11H, 2 Ph, $CH_3CON\underline{H}$). ^{13}C NMR (75 MHz, $CDCl_3$, main rotamer) δ 14.09, 22.67, 29.34, 29.37, 29.61, 29.65, 29.69, 29.78, 31.91, 39.14, 41.60, 43.90, 49.61, 51.18, 59.04, 59.76, 125.29, 126.32, 128.21, 128.60, 129.02, 129.13, 134.96, 138.05, 168.83, 169.88, 170.64. HRMS ESI m/z: $[M+H]^+$ calcd for $C_{40}H_{65}N_4O_3$ 649.5051, found: 649.5051.

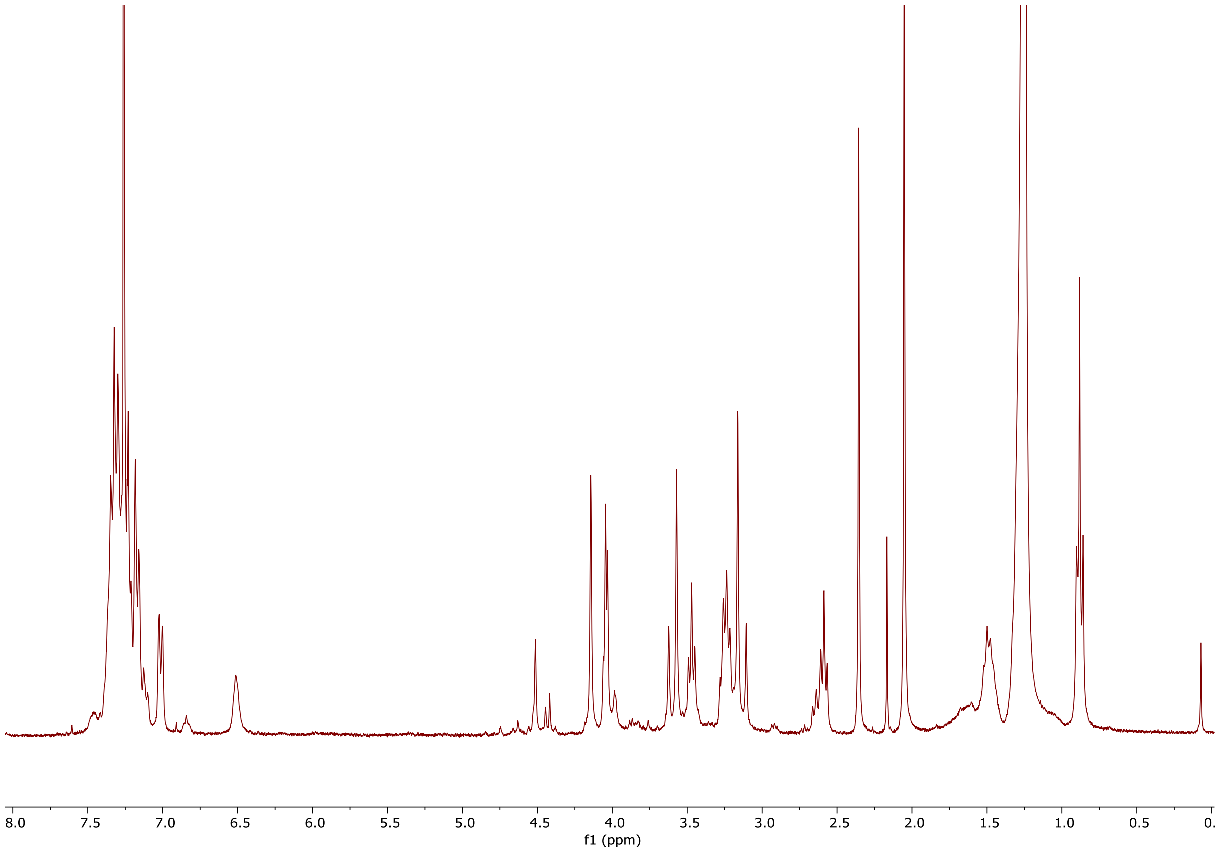

Figure S4. ^1H NMR spectrum of compound **11b.**

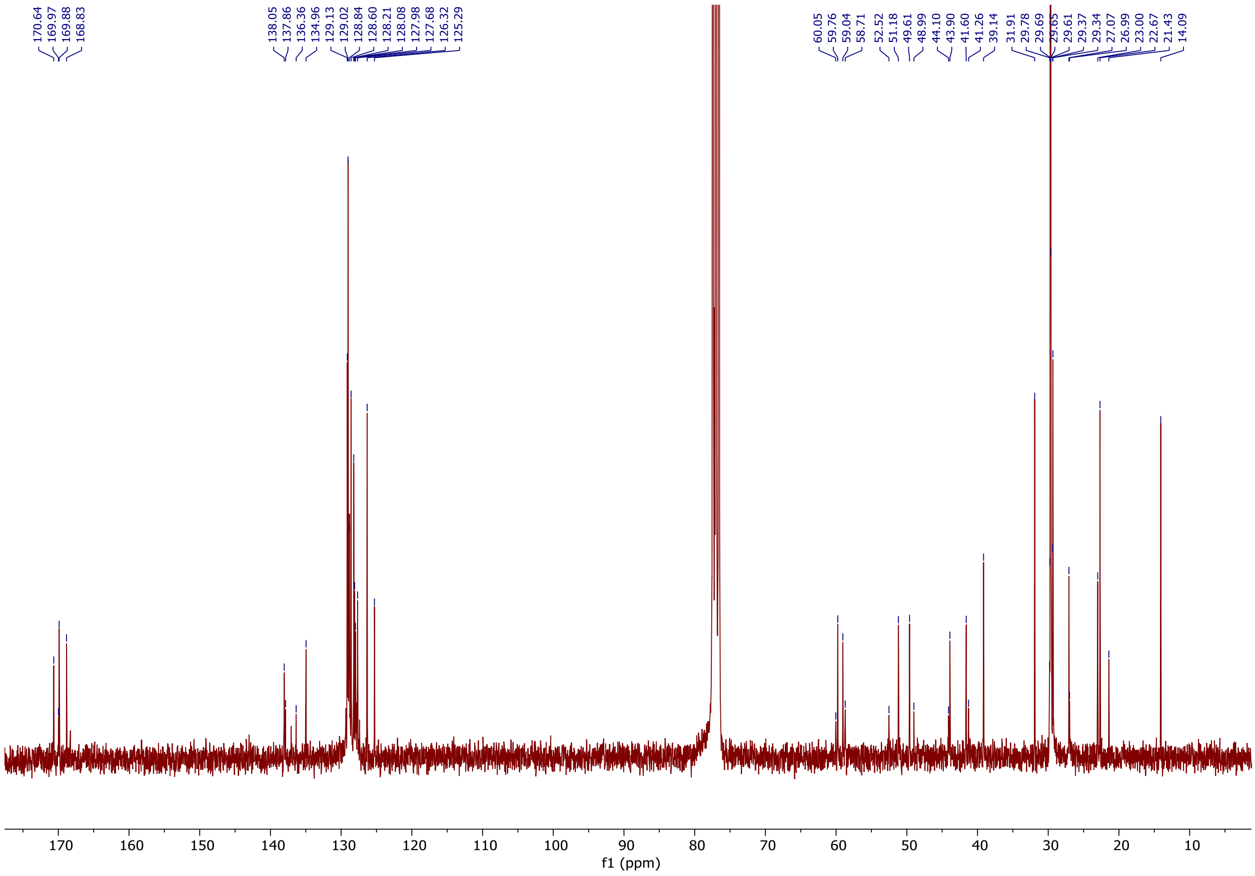

Figure S5. ^{13}C NMR spectrum of compound **11b.**

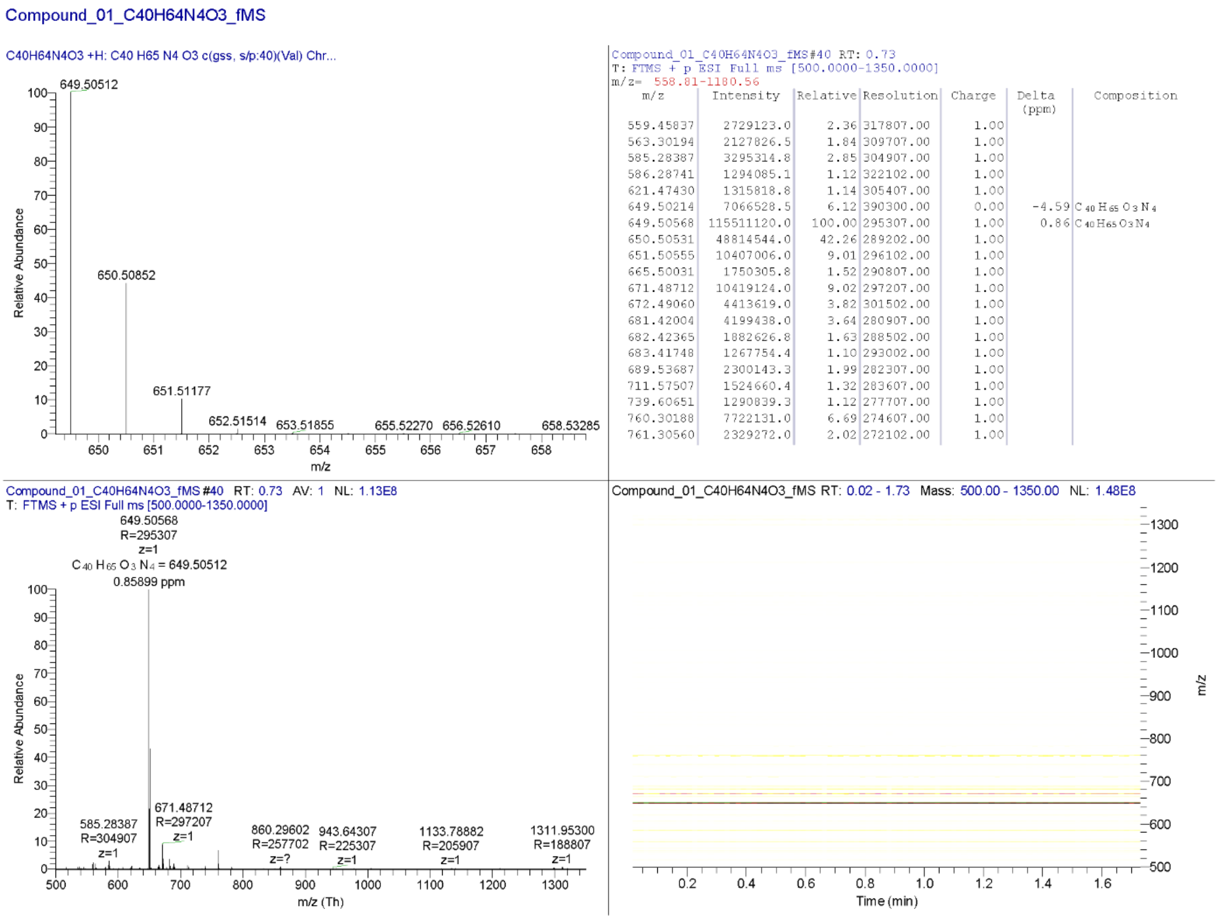

Figure S6. HRMS spectrum of compound **11b.**

1,9-diamino-*N*[9]-acetyl-*N*[1]-octadecyl-1,8-dioxo-*N*[3],*N*[7]-dibenzyl-3,7-diazanonane (**11c**)

Yield: 48%, colorless oil. Eluent: EA-MeOH (9:1). ^{1}H NMR (400 MHz, $CDCl_3$, COSY, HSQC, HMBC) δ 0.87 (t, 3H, *J* = 6.9 Hz, $(CH_2)_{15}C\underline{H}_3$), 1.25 (br. s, 30H, $(C\underline{H}_2)_{15}CH_3$), 1.38 – 1.52 (m, 2H, $CH_2C\underline{H}_2(CH_2)_{15}$), 1.64 – 1.80 (m, 2H, $NCH_2C\underline{H}_2CH_2N$), 2.04 (s, 3H, $COCH_3$), 2.36 – 2.58 (m, 2H, $PhCH_2NC\underline{H}_2$), 3.06 (s, 2H, $COCH_2N$), 3.09 – 3.31 (m, 3H, $C\underline{H}_2CH_2(CH_2)_{15}$, $PhCH_2N(CO)C\underline{H}_2$), 3.39 (t, 1H, *J* = 7.3 Hz, $PhCH_2N(CO)C\underline{H}_2$), 3.57 (s, 2H, $COC\underline{H}_2NH$), 4.05 (d, 2H, *J* = 3.9 Hz, $PhCH_2$), 4.41 (s, 2H, $PhCH_2NCO$), 6.57 (s, 1H, $N\underline{H}COCH_2$), 6.84 – 7.54 (m, 11H, 2 Ph, $CH_3CON\underline{H}$). ^{13}C NMR (101 MHz, $CDCl_3$) δ 14.09, 22.67, 22.99, 26.97, 29.31, 29.34, 29.58, 29.61, 29.64, 29.66, 29.69, 31.91, 39.04, 41.51, 44.21, 48.78, 50.08, 51.98, 57.97, 59.91, 126.30, 127.87, 128.06, 128.71, 128.80, 129.13, 135.27, 136.60, 168.06, 170.00, 170.10. HRMS ESI m/z: $[M+H]^+$ calcd for $C_{41}H_{67}N_4O_3$ 663.5208, found: 663.5196. HRMS ESI m/z: $[M+Na]^+$ calcd for $C_{41}H_{66}NaN_4O_3$ 685.5033, found: 685.5001.

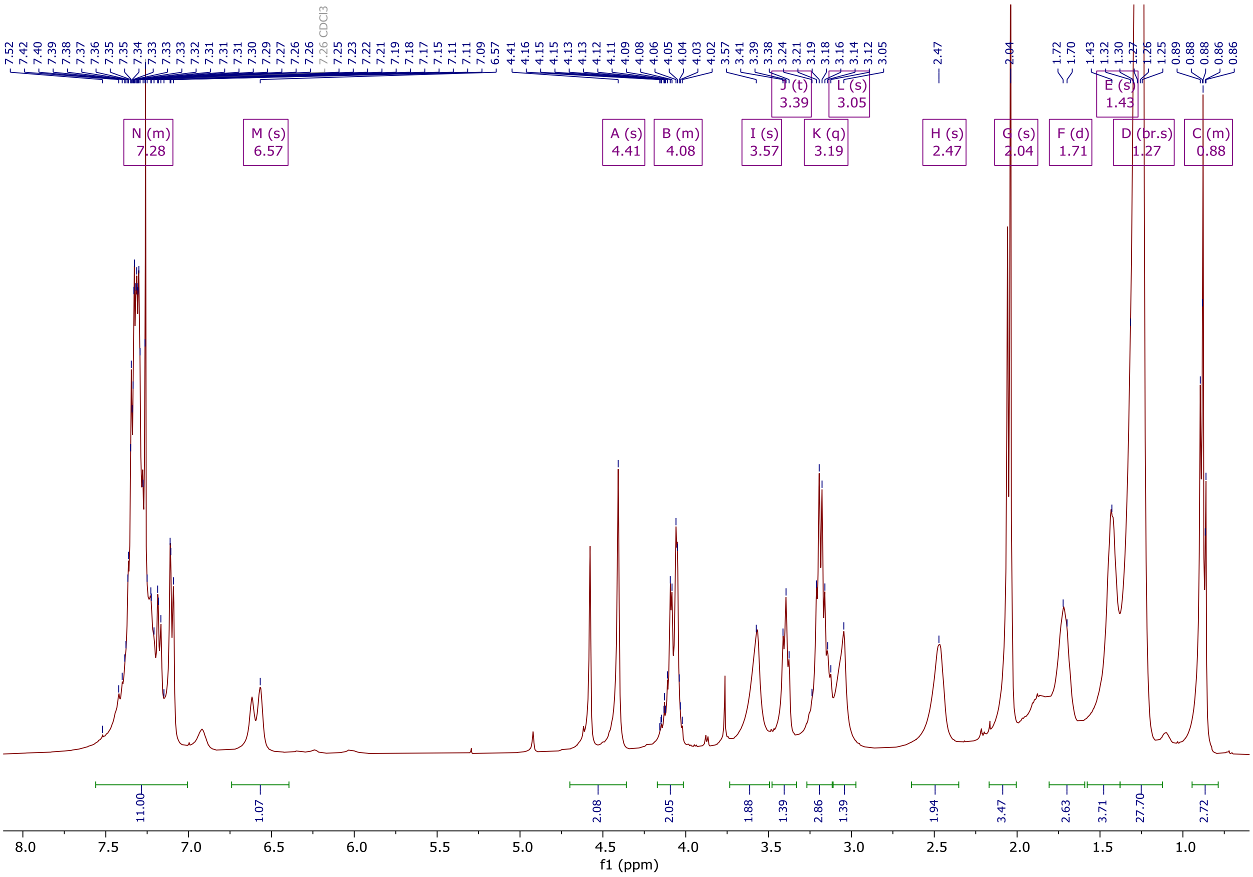

Figure S7. ^{1}H NMR spectrum of compound **11c.**

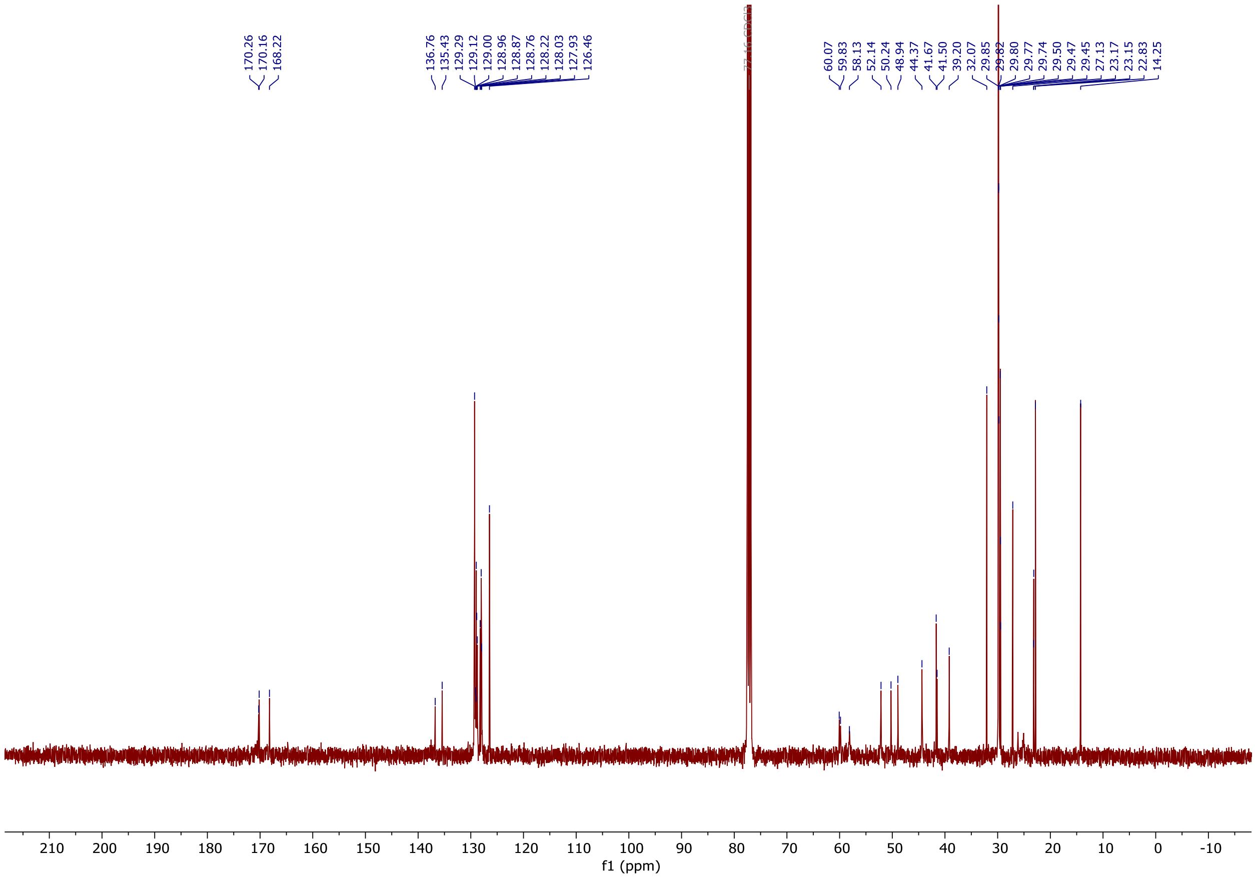

Figure S8. ^{13}C NMR spectrum of compound **11c.**

3a-128

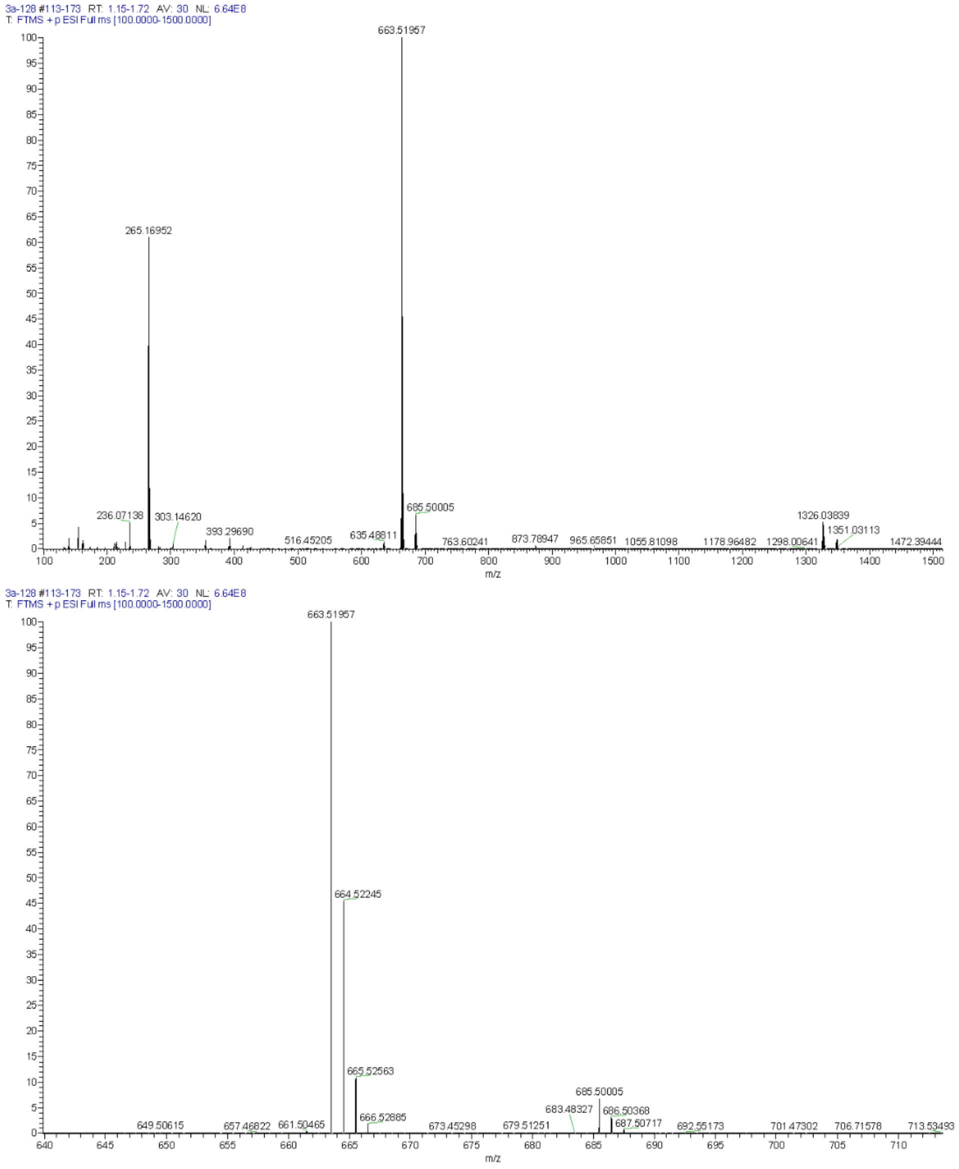

Figure S9. HRMS spectrum of compound **11c.**

1,6-diamino-*N*[1]-acetyl-*N*[6]-octadecyl-6-oxo-*N*[1],*N*[3]-dibenzyl-4-azahexane (**11d**)

Yield: 35%, colorless oil. Eluent: PE-EA (4:6). ^{1}H NMR (400 MHz, $CDCl_3$) δ 0.80 (t, 3H, J = 6.6 Hz, $(CH_2)_{15}C\underline{H}_3$), 1.18 (br. s, 30H, $(C\underline{H}_2)_{15}CH_3$), 1.29 – 1.41 (m, 2H, $CH_2C\underline{H}_2(CH_2)_{15}$), 1.55 – 1.70 (m, 2H, $NCH_2C\underline{H}_2CH_2N$), 2.01 (s, 3H, $COCH_3$), 2.31 – 2.43 (m, 2H, $PhCH_2NC\underline{H}_2$), 2.96 (s, 2H, $COCH_2N$), 3.05 – 3.17 (m, 2H, $C\underline{H}_2CH_2(CH_2)_{15}$), 3.29 (t, 2H, J = 7.5 Hz, $PhCH_2N(CO)C\underline{H}_2$), 3.48 (s, 2H, $PhCH_2$), 4.37 (s, 2H, $PhCH_2NCO$), 6.76 – 7.38 (m, 11H, 2 Ph, N$\underline{H}$). ^{13}C NMR (101 MHz, $CDCl_3$) δ 13.99, 21.29, 21.65, 22.56, 25.09, 26.09, 26.85, 29.16, 29.19, 29.23, 29.47, 29.50, 29.54, 29.58, 31.80, 38.83, 38.86, 43.58, 45.96, 48.18, 51.85, 52.14, 52.27, 57.94, 59.61, 126.11, 127.30, 127.32, 127.46, 127.57, 127.59, 127.86, 128.23, 128.38, 128.48, 128.50, 128.55, 128.64, 128.69, 128.73, 128.84, 128.95, 129. HRMS ESI m/z: $[M+H]^+$ calcd for $C_{39}H_{63}N_3O_2$ 606.4993, found: 606.4991.

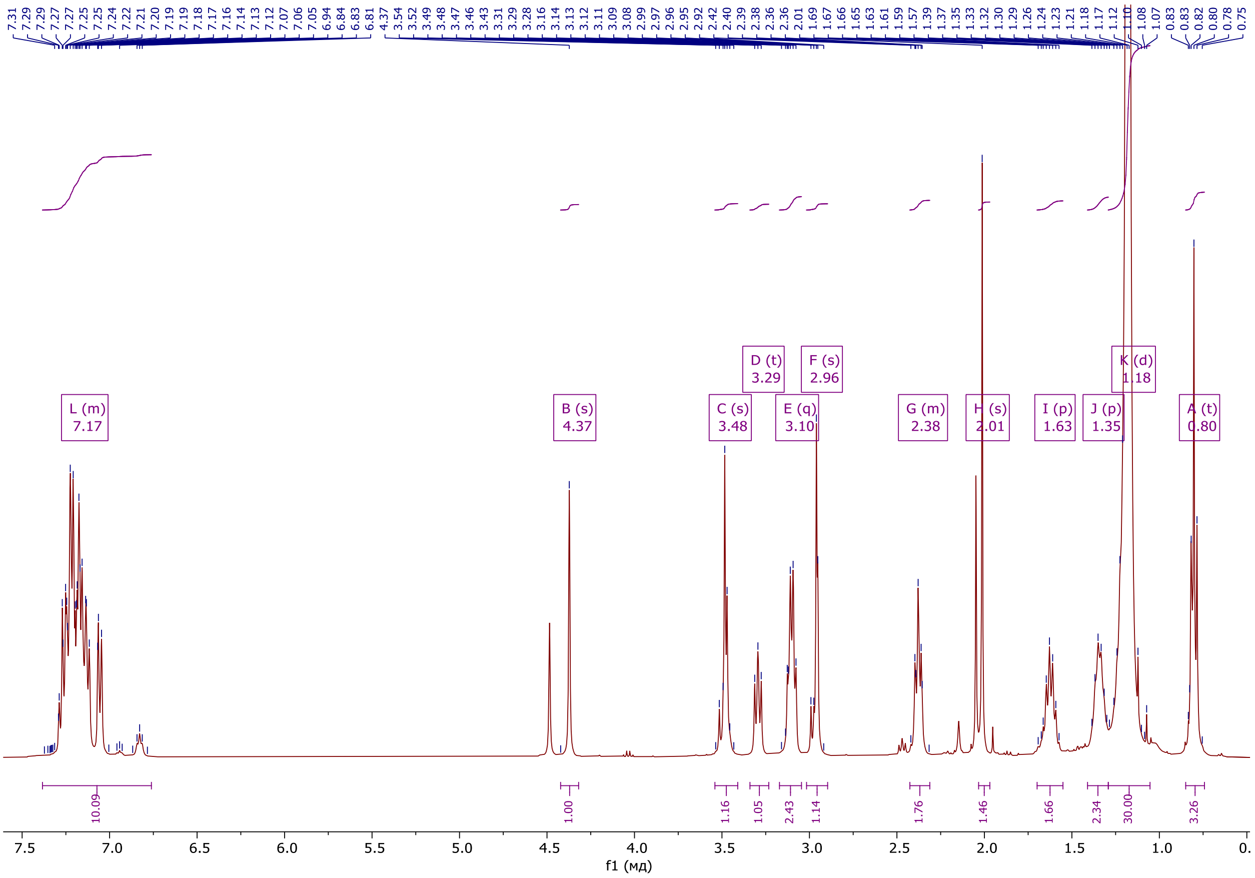

Figure S10. ^{1}H NMR spectrum of compound **11d.**

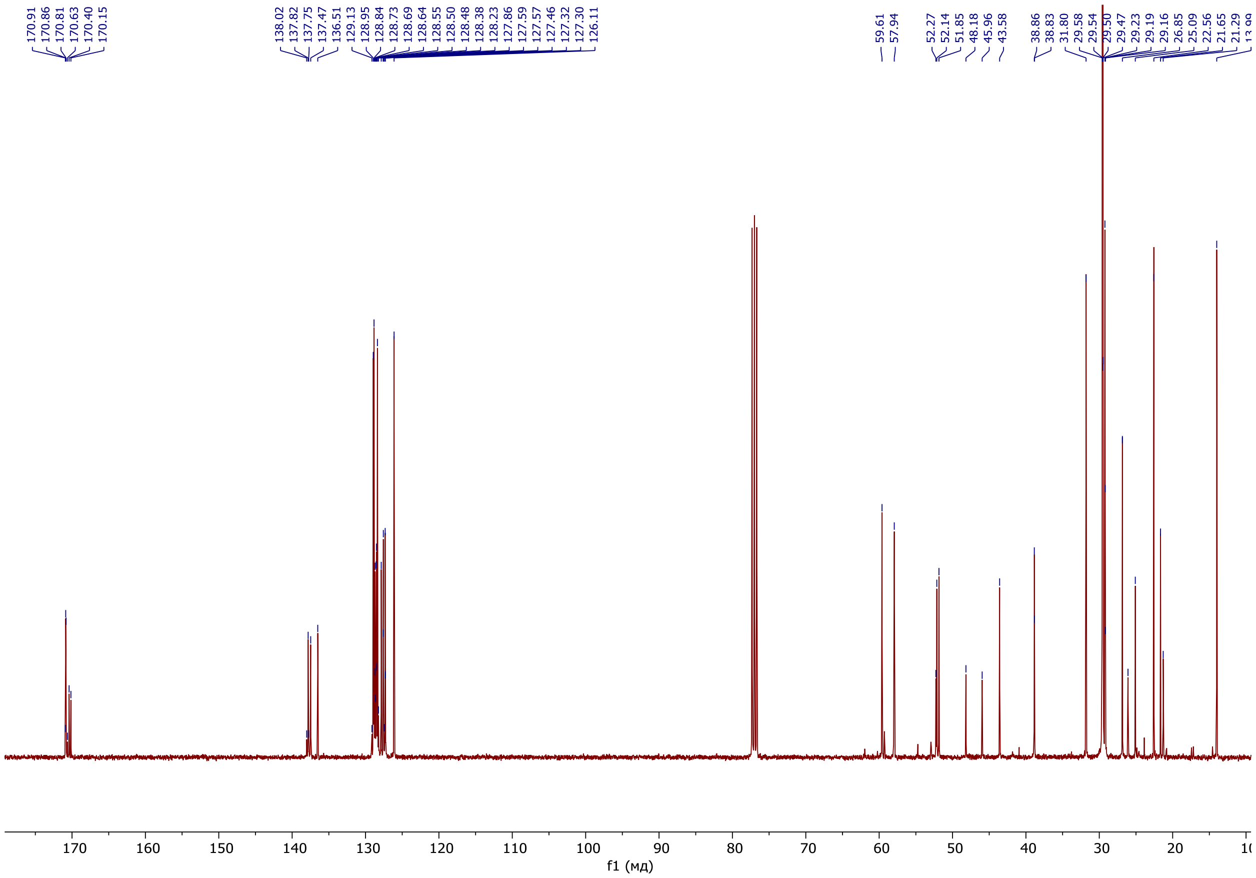

Figure S11. ^{13}C NMR spectrum of compound **11d.**

3a-118

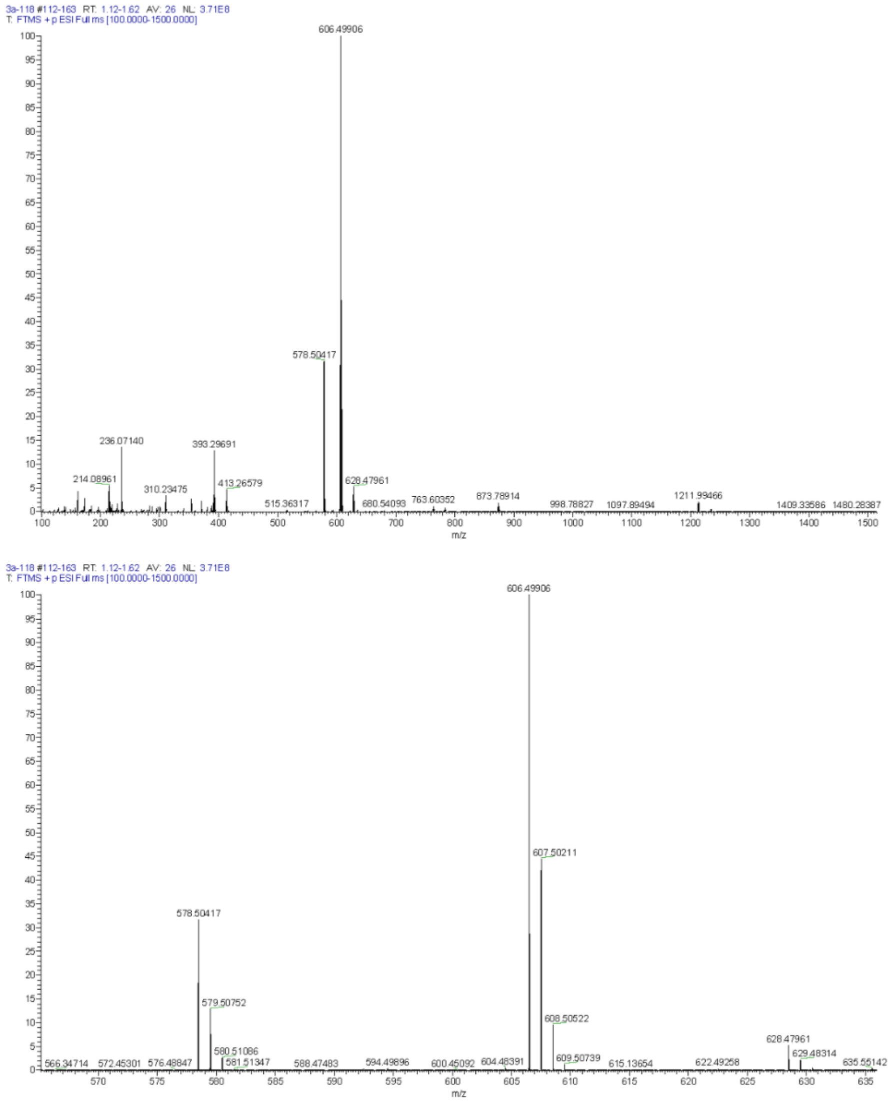

Figure S12. HRMS spectrum of compound **11d.**

1,7-diamino-*N*[1]-acetyl-*N*[7]-octadecyl-7-oxo-*N*[1],*N*[4]-dibenzyl-5-azaheptane (**11e**)

Yield: 30%, colorless oil. Eluent: EA. ^{1}H NMR (300 MHz, main rotamer, $CDCl_3$) δ 0.71 (t, 3H, *J* = 6.7 Hz, $(CH_2)_{15}C\underline{H}_3$), 1.12 (br.s, 30H, $(C\underline{H}_2)_{15}CH_3$,), 1.19 – 1.41 (m, 6H, $CH_2C\underline{H}_2(CH_2)_{15}$, $NCH_2(C\underline{H}_2)_2CH_2N$), 1.93 (s, 3H, $COCH_3$), 2.21 – 2.36 (m, 2H $PhCH_2NC\underline{H}_2$,), 2.88 (s, 2H, NCH_2CO), 2.94 – 3.22 (m, 4H, $COCH_2N$, $C\underline{H}_2CH_2(CH_2)_{15}$), 3.40 (s, 2H, $PhCH_2$), 4.32 (s, 2H, $PhCH_2NCO$), 6.78 – 7.31 (m, 2 Ph, NH, 11H). ^{13}C NMR (75 MHz, $CDCl_3$) δ 14.22, 21.56, 21.88, 22.78, 24.67, 25.32, 26.37, 27.06, 29.44, 29.79, 32.01, 39.01, 46.01, 47.88, 48.26, 52.18, 54.80, 58.14, 59.65, 59.92, 126.25, 127.48, 127.70, 128.01, 128.64, 128.83, 128.93, 129.03, 136.90, 137.74, 138.08, 138.24, 170.94, 171.15. HRMS FTICR m/z: $[M+H]^+$ calcd for $C_{40}H_{66}N_3O_2$ 620.5150, found: 620.5136.

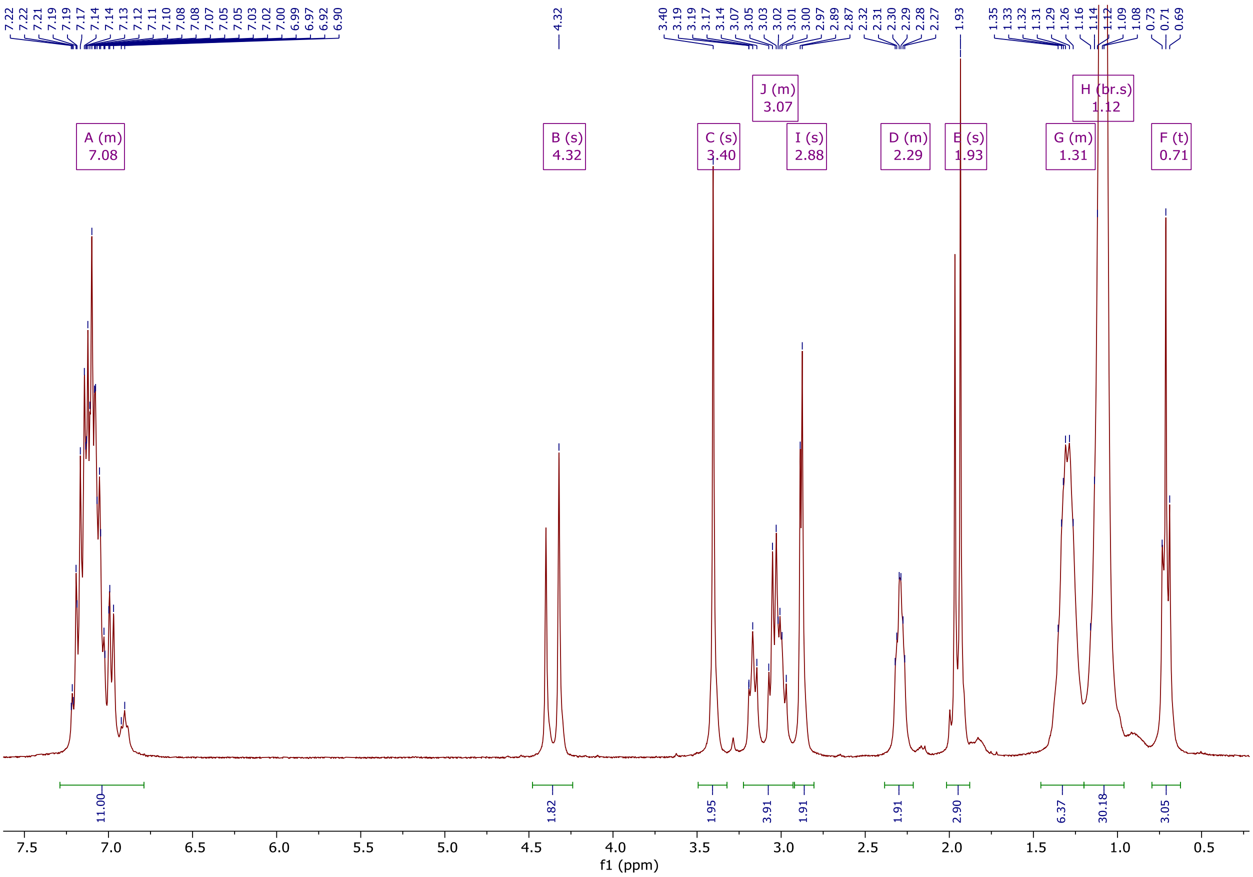

Figure S13. ^{1}H NMR spectrum of compound **11e.**

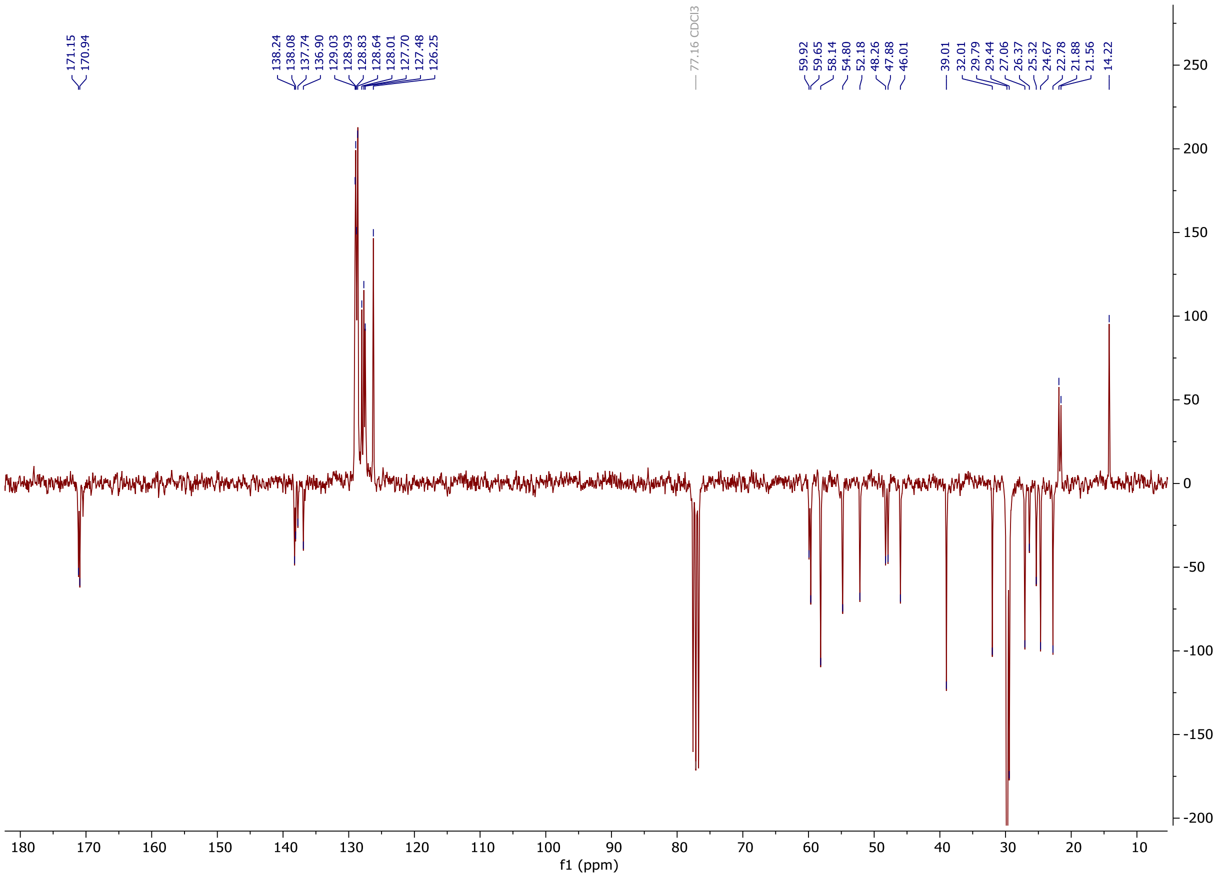

Figure S14. ^{13}C (APT) NMR spectrum of compound **11e.**

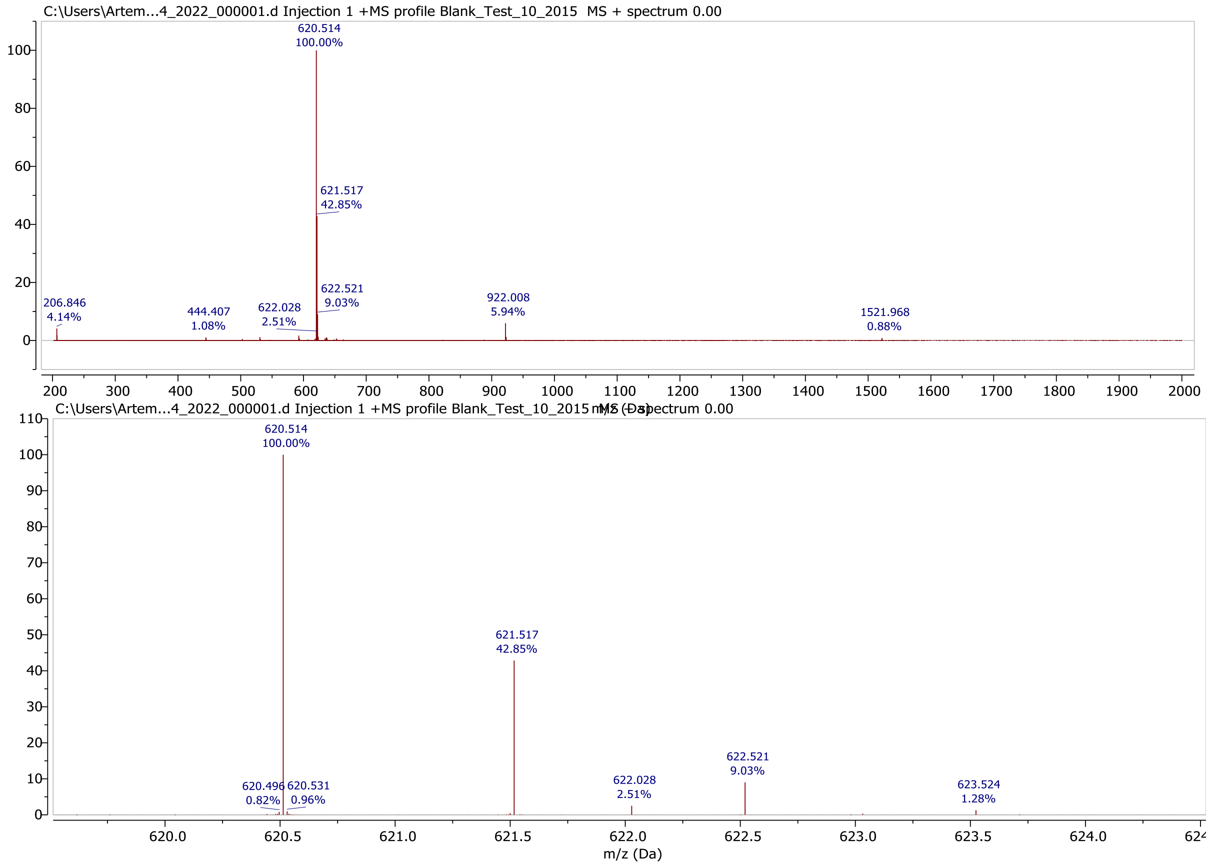

Figure S15. HRMS spectrum of compound **11e.**

N^1-(N-acetylglycyl)-N^4-[(N-octadecyl)aminocarbonyl]methylpiperazin (**12a**)

Yield: 60%, colorless oil. Eluent: DCM-MeOH (20:1). ^{1}H NMR (300 MHz, $CDCl_3$, ^{1}H-^{1}H COSY) δ 0.84 (d, 3H, J = 6.9 Hz $(CH_2)_{15}C\underline{H}_3$), 1.22 (br. s, 30H, $(C\underline{H}_2)_{15}CH_3$), 1.41 – 1.54 (m, 2H, $CH_2C\underline{H}_2(CH_2)_{15}$), 2.01 (s, 3H, $COCH_3$), 2.47 – 2.55 (m, 4H, 2 $COCH_2NC\underline{H}_2$ Pip), 3.00 (s, 2H, $COCH_2N$), 3.19 – 3.29 (m, 2H, $C\underline{H}_2CH_2(CH_2)_{15}$), 3.38 – 3.46 (m, 2H, 2 $CONC\underline{H}_eH_a$ Pip), 3.60 – 3.66 (m, 2H, 2 $CONCH_e\underline{H}_a$ Pip), 4.02 (d, 2H, J = 4.1 Hz, $COC\underline{H}_2NH$), 6.61 (t, 1H, J = 4.1 Hz, $N\underline{H}COCH_3$), 6.92 (t, 1H, J = 5.5 Hz, CH_2CONH). ^{13}C NMR (75 MHz, $CDCl_3$) δ 14.06, 22.64, 22.93, 26.95, 29.24, 29.31, 29.53, 29.56, 29.61, 29.65, 29.67, 31.88, 39.00, 41.23, 41.98, 44.35, 52.97, 53.19, 61.49, 166.55, 169.00, 170.08. HRMS FTICR m/z: $[M+H]^+$ calcd for $C_{28}H_{55}N_4O_3$ 495.4269, found: 495.4269.

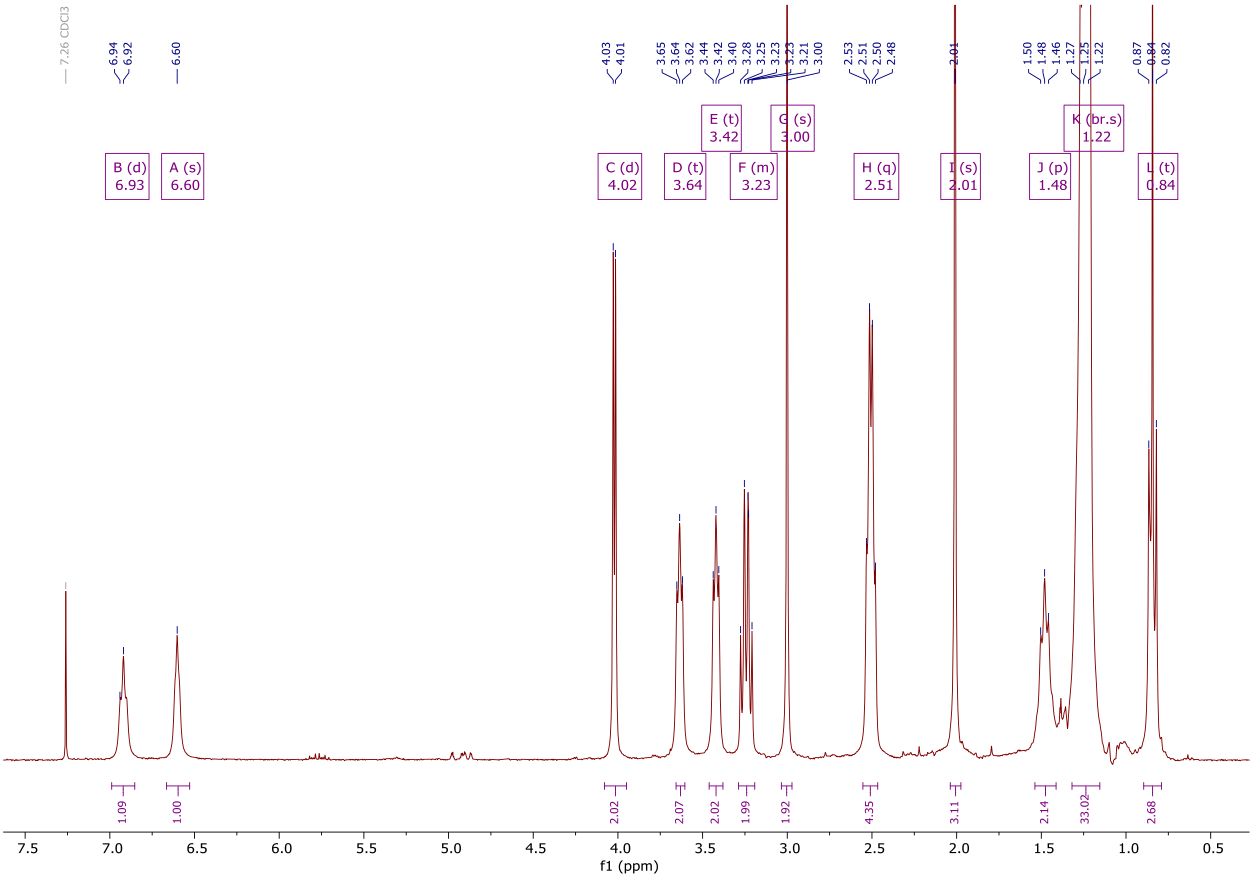

Figure S16. ^{1}H NMR spectrum of compound **12a.**

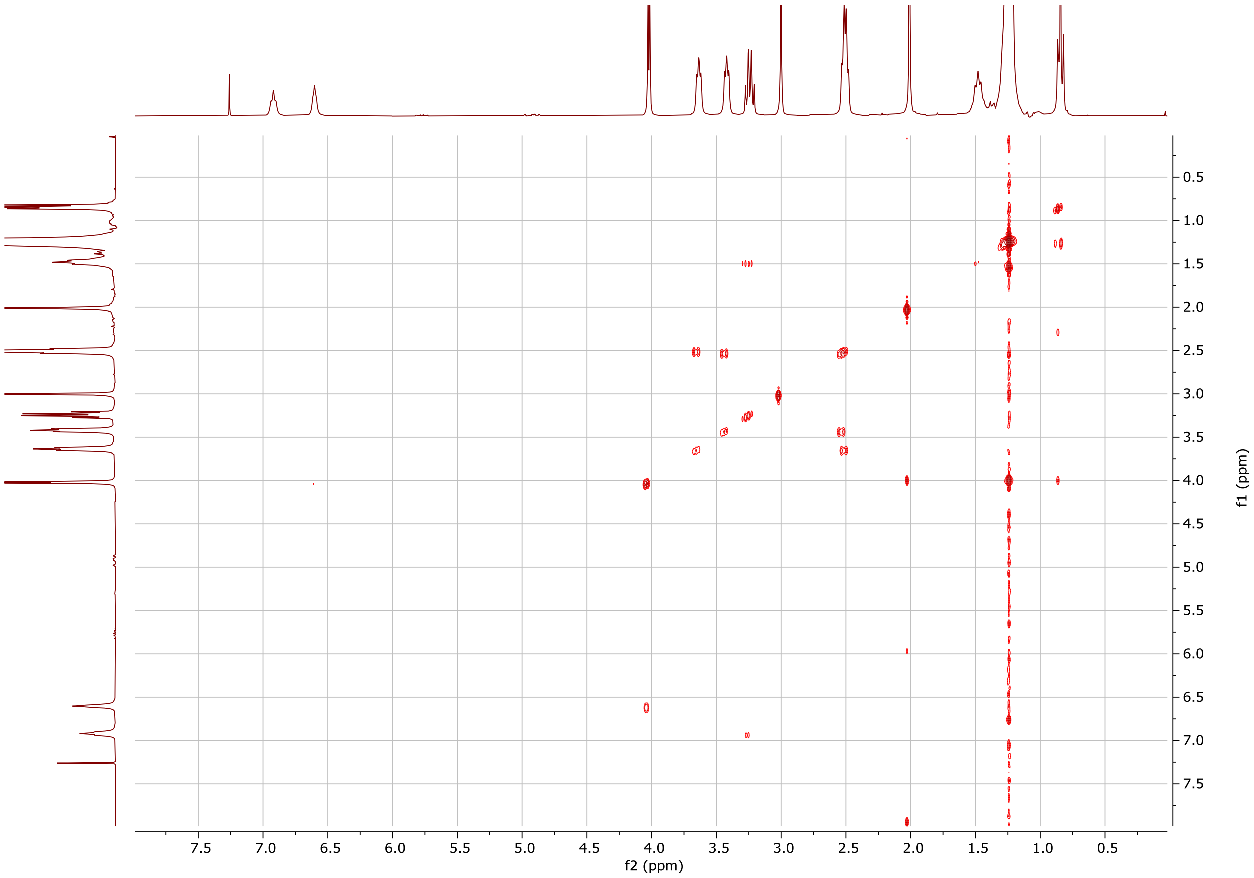

Figure S17. {^{1}H-^{1}H} COSY NMR spectrum of compound **12a.**

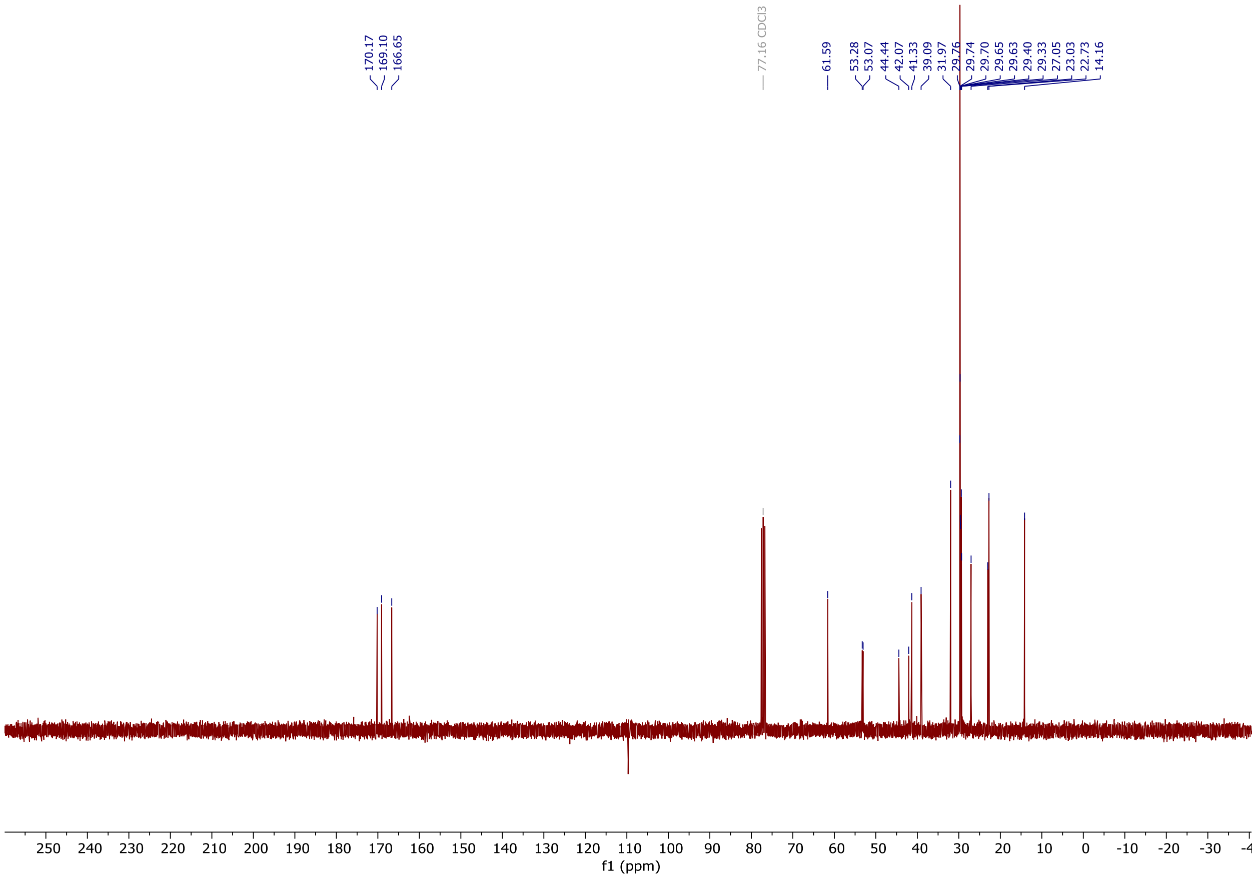

Figure S18. ^{13}C NMR spectrum of compound **12a.**

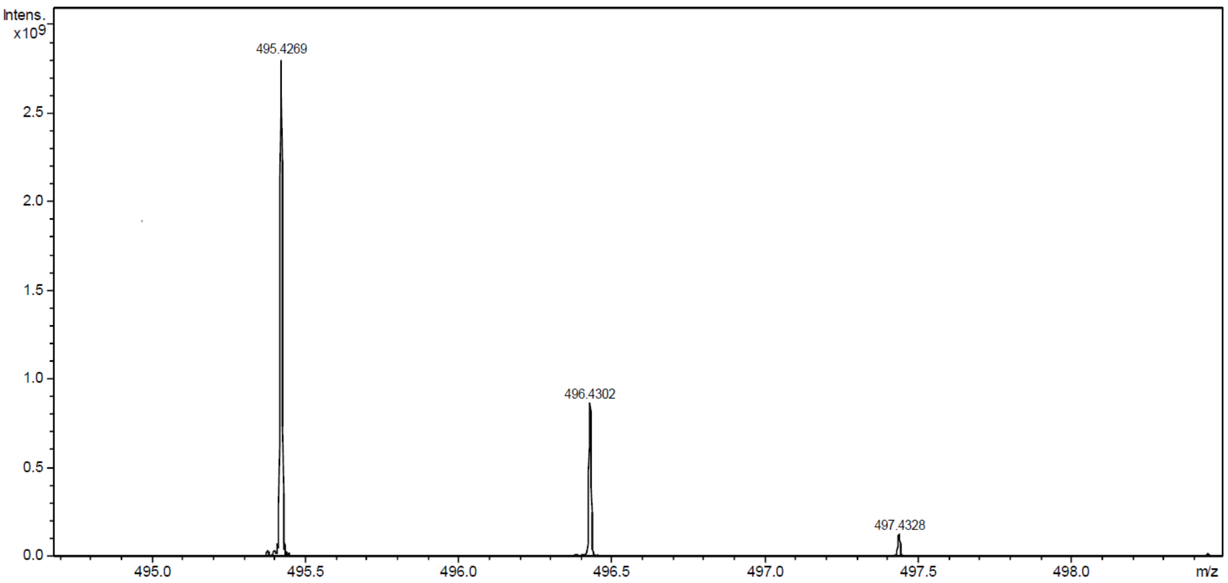

Figure S19. HRMS spectrum of compound **12a.**

N^1-acetyl-N^4-[(N-octadecyl)aminocarbonyl]methylpiperazin (**12b**)

Yield: 80%, colorless oil. Eluent: DCM-MeOH (30:1). ^{1}H NMR (300 MHz, $CDCl_3$, ^{1}H-^{1}H COSY) δ 0.90 (t, 3H, J = 7.0 Hz, $(CH_2)_{15}C\underline{H}_3$), 1.26 (br. s, 30H, $(C\underline{H}_2)_{15}CH_3$), 1.48 – 1.61 (m, 2H, $CH_2C\underline{H}_2(CH_2)_{15}$), 2.11 (s, 3H, $COCH_3$), 2.49 – 2.59 (m, 4H, 2 $COCH_2NC\underline{H}_2$ Pip), 3.05 (s, 2H, $COCH_2N$), 3.20 – 3.37 (m, 2H, $C\underline{H}_2CH_2(CH_2)_{15}$), 3.46 – 3.54 (m, 2H, 2 $CONC\underline{H}_eH_a$ Pip), 3.63 – 3.69 (m, 2H, 2 $CONCH_e\underline{H}_a$ Pip), 7.06 (br. s, 1H, NH). ^{13}C NMR (75 MHz, $CDCl_3$) δ 14.09, 21.28, 22.66, 26.94, 29.24, 29.32, 29.54, 29.57, 29.62, 29.66, 31.89, 38.98, 41.32, 46.15, 53.12, 53.45, 61.47, 168.95, 169.13. HRMS FTICR m/z: $[M+H]^+$ calcd for $C_{28}H_{55}N_4O_3$ 438.4054, found: 438.4054.

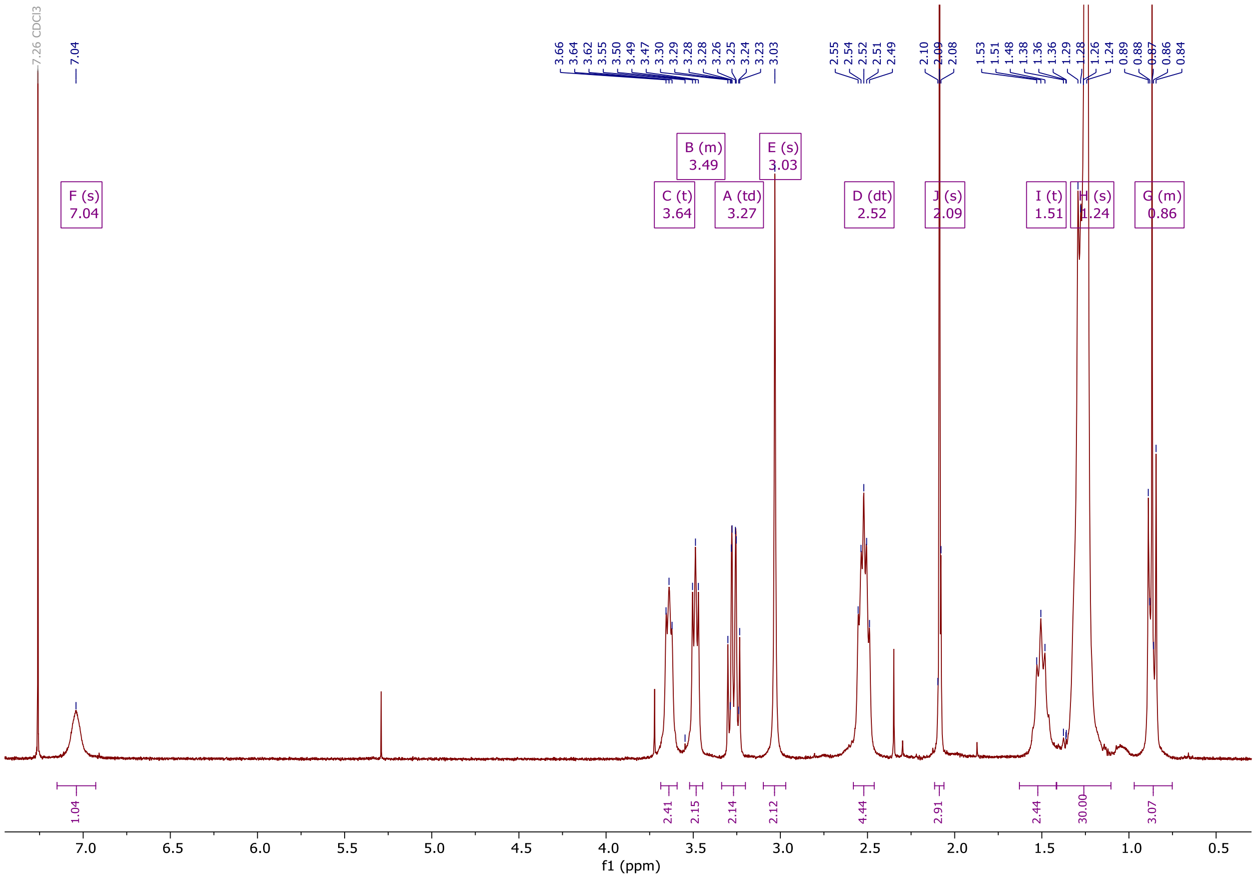

Figure S20. ^{1}H NMR spectrum of compound **12b.**

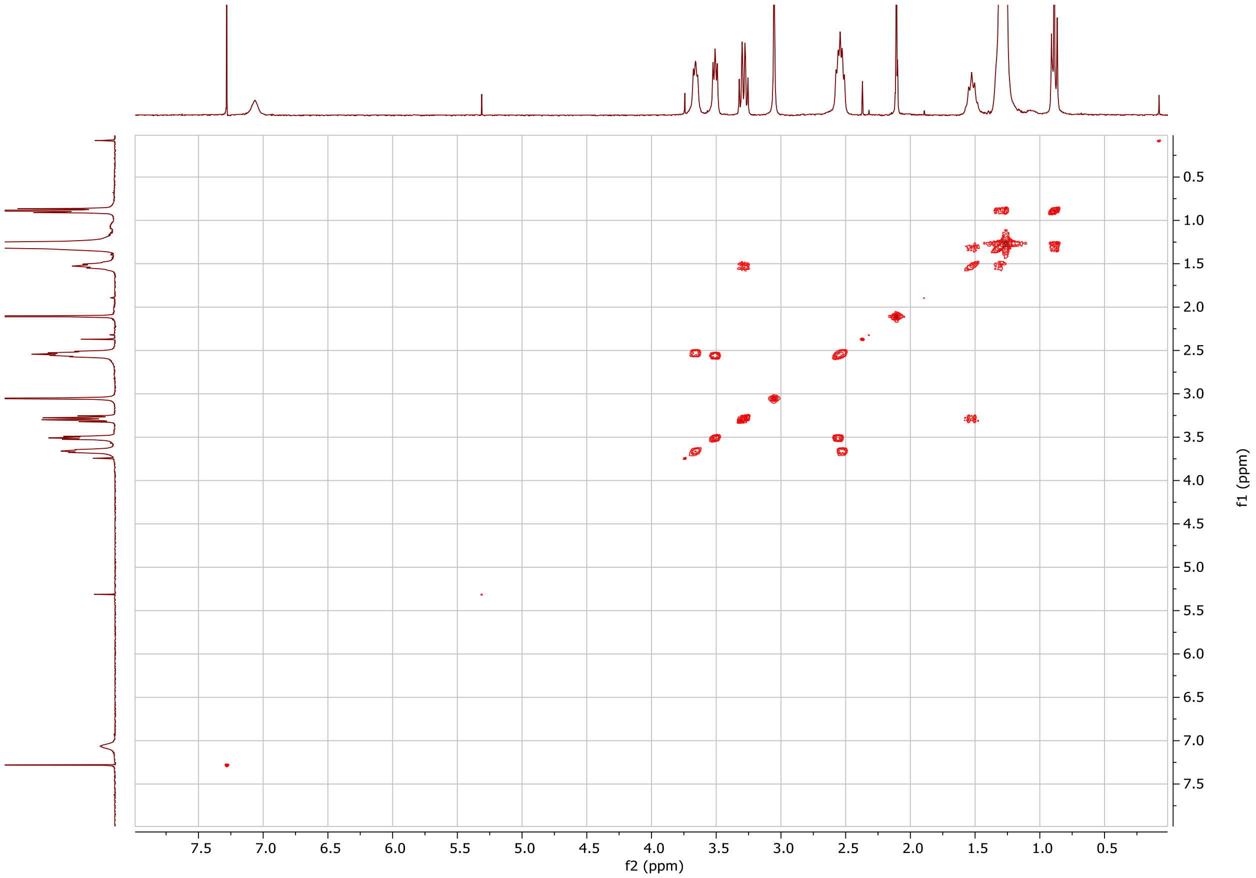

Figure S21. {^{1}H-^{1}H} COSY NMR spectrum of compound **12b.**

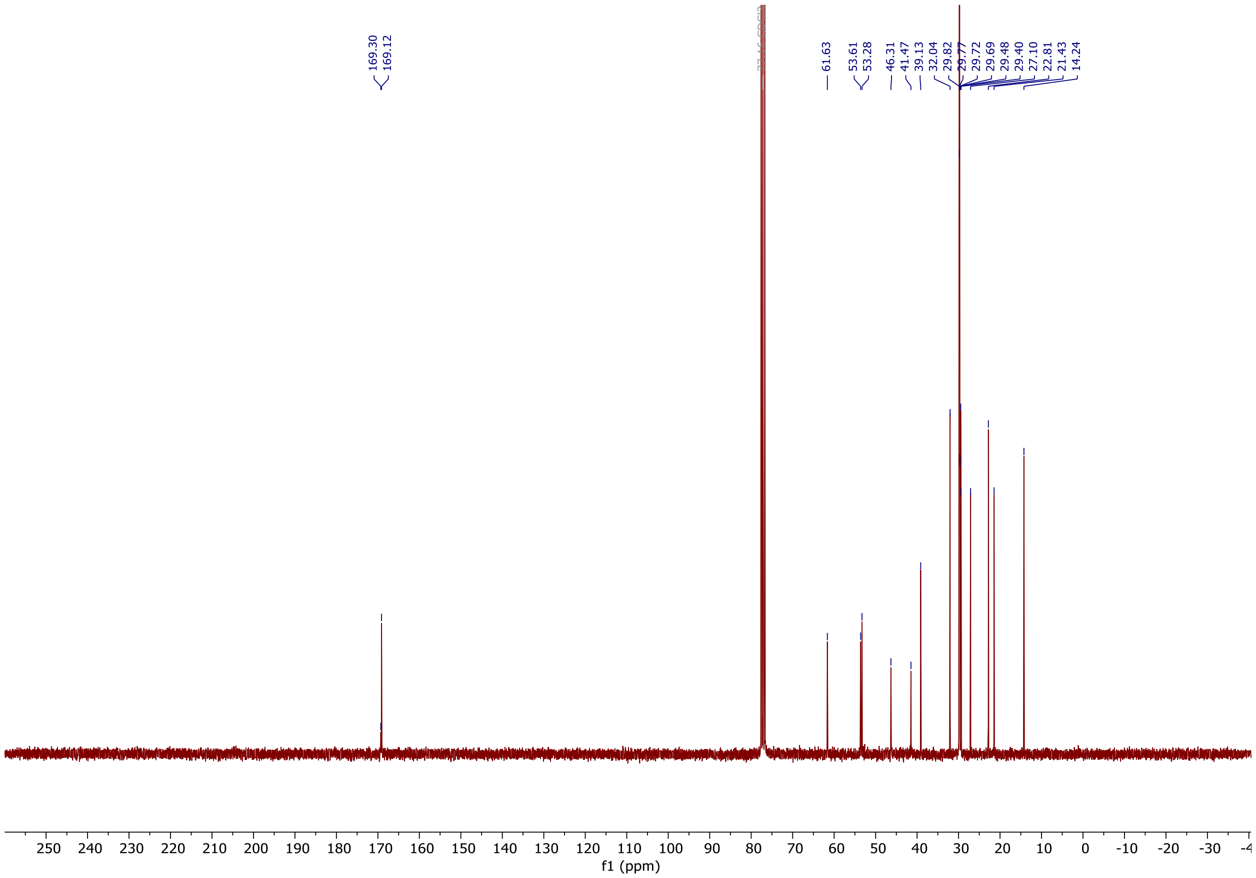

Figure S22. ^{13}C NMR spectrum of compound **12b.**

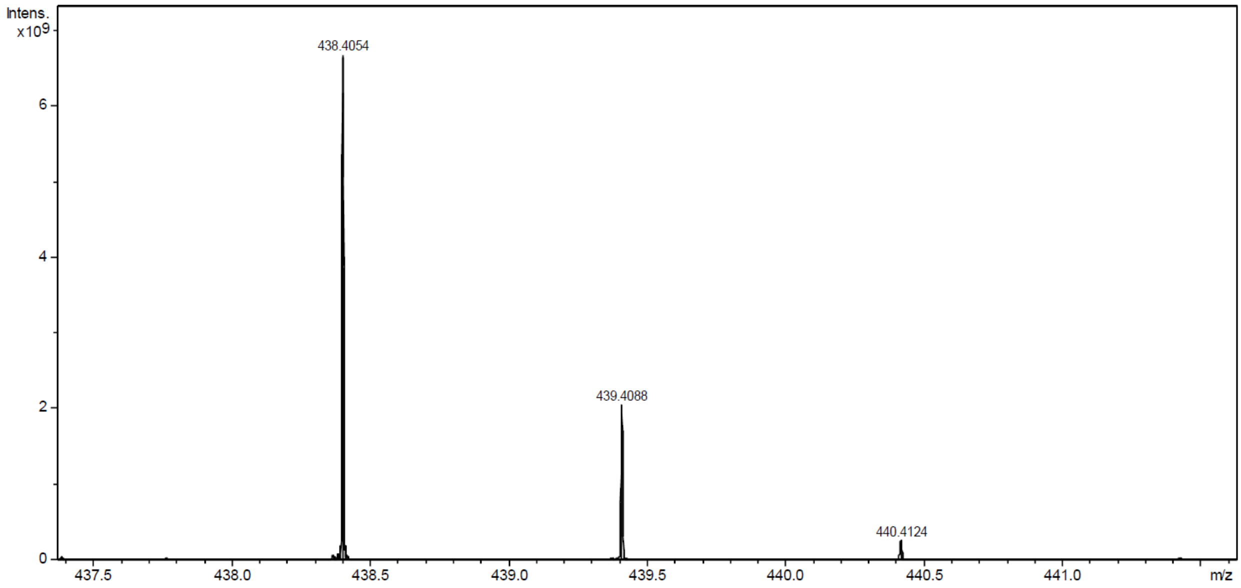

Figure S23. HRMS spectrum of compound **12b.**

N^1-(N-acetylglycyl)-N^4-[N-(*rac*-1-decyloxy-2-ethyloxyprop-3-yl)aminocarbonyl]methylpiperazin (**12c**)

Yield: 47%, colorless oil. Eluent: DCM-MeOH (15:1). ^{1}H NMR (600 MHz, DMSO-*d6*, COSY, HSQC, HMBC) δ 0.85 (t, 3H, J = 6.9 Hz, $(CH_2)_7C\underline{H}_3$), 1.09 (t, 3H, J = 7.0 Hz, $OCH_2C\underline{H}_3$), 1.24 (br. s, 14H, $(C\underline{H}_2)_7CH_3$), 1.43 – 1.51 (m, 2H, $OCH_2C\underline{H}_2$), 1.86 (s, 3H, $COCH_3$), 2.34 – 2.42 (m, 2H, 2 $COCH_2NC\underline{H}_eH_a$ Pip), 2.42 – 2.48 (m, 2H, 2 $COCH_2NCH_e\underline{H}_a$ Pip), 2.93 (d, J = 15.5 Hz, 1H, $COC\underline{H}_aH_bN$), 2.96 (d, J = 15.5 Hz, 1H, $COCH_a\underline{H}_bN$), 3.07 – 3.13 (m, 1H, $CONHC\underline{H}_aH_b$), 3.25 – 3.32 (m, 1H, $NHCH_a\underline{H}_bCH$), 3.33 – 3.40 (m, 4H, $C\underline{H}_2OC\underline{H}_2$), 3.40 – 3.52 (m, 6H, $C\underline{H}OC\underline{H}_aH_bCH_3$, 2 $CONCH_2$ Pip), 3.52 – 3.59 (m, 1H, $OCH_a\underline{H}_bCH_3$), 3.92 (d, 2H, J = 5.5 Hz, $COC\underline{H}_2NH$), 7.63 -7.70 (m, 1H, $CHCH_2N\underline{H}$), 7.91 (t, 1H, J = 5.5 Hz, $COCH_2N\underline{H}$). ^{13}C NMR (151 MHz, DMSO-*d6*) δ 13.86, 15.50, 22.04, 22.35, 25.57, 28.66, 28.79, 28.95, 28.97, 28.99, 29.00, 29.11, 31.25, 39.59, 40.32, 41.27, 44.00, 52.38, 52.74, 60.87, 64.39, 70.59, 71.05, 76.33, 166.95, 168.84, 169.19. HRMS FTICR m/z: $[M+H]^+$ calcd for $C_{27}H_{53}N_4O_5$ 513.4010, found: 513.4010.

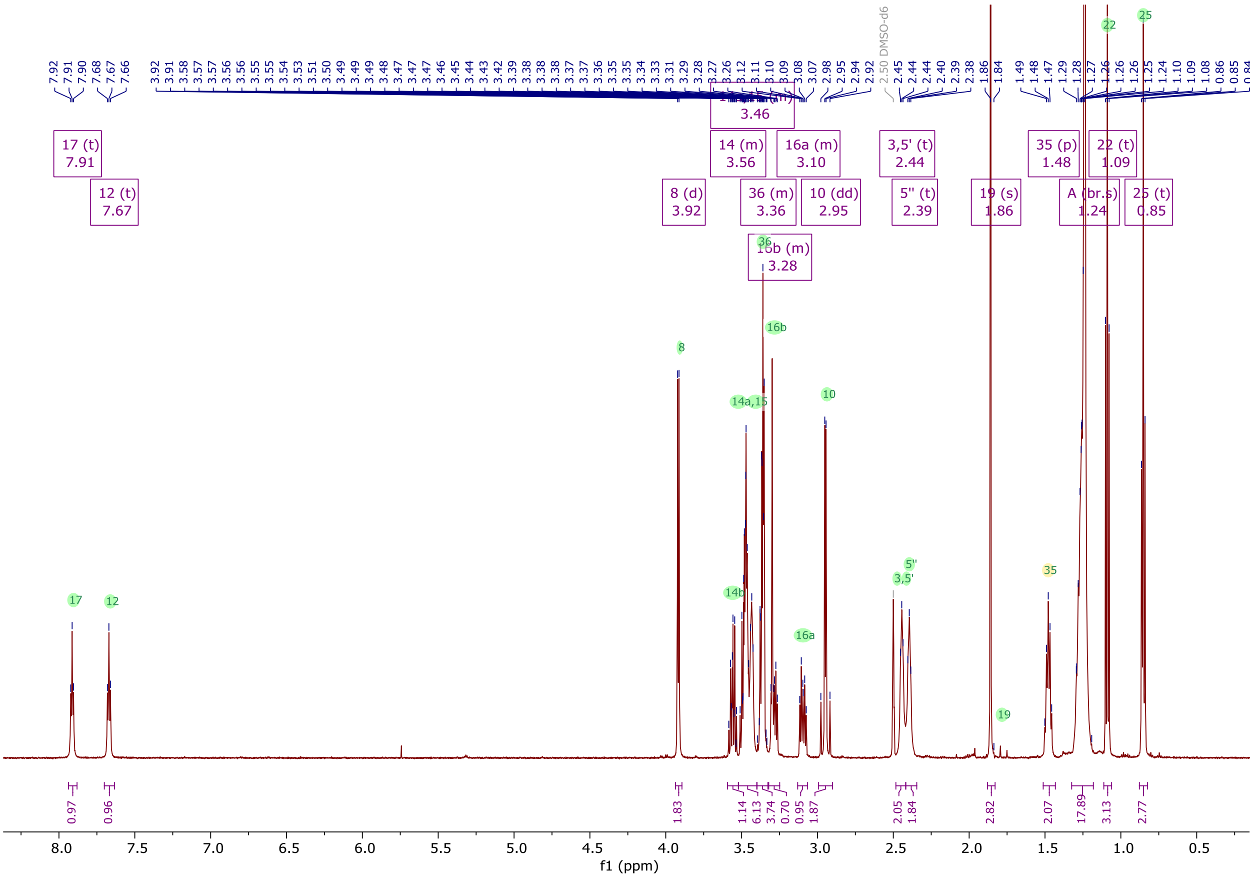

Figure S24. ^{1}H NMR spectrum of compound **12c.**

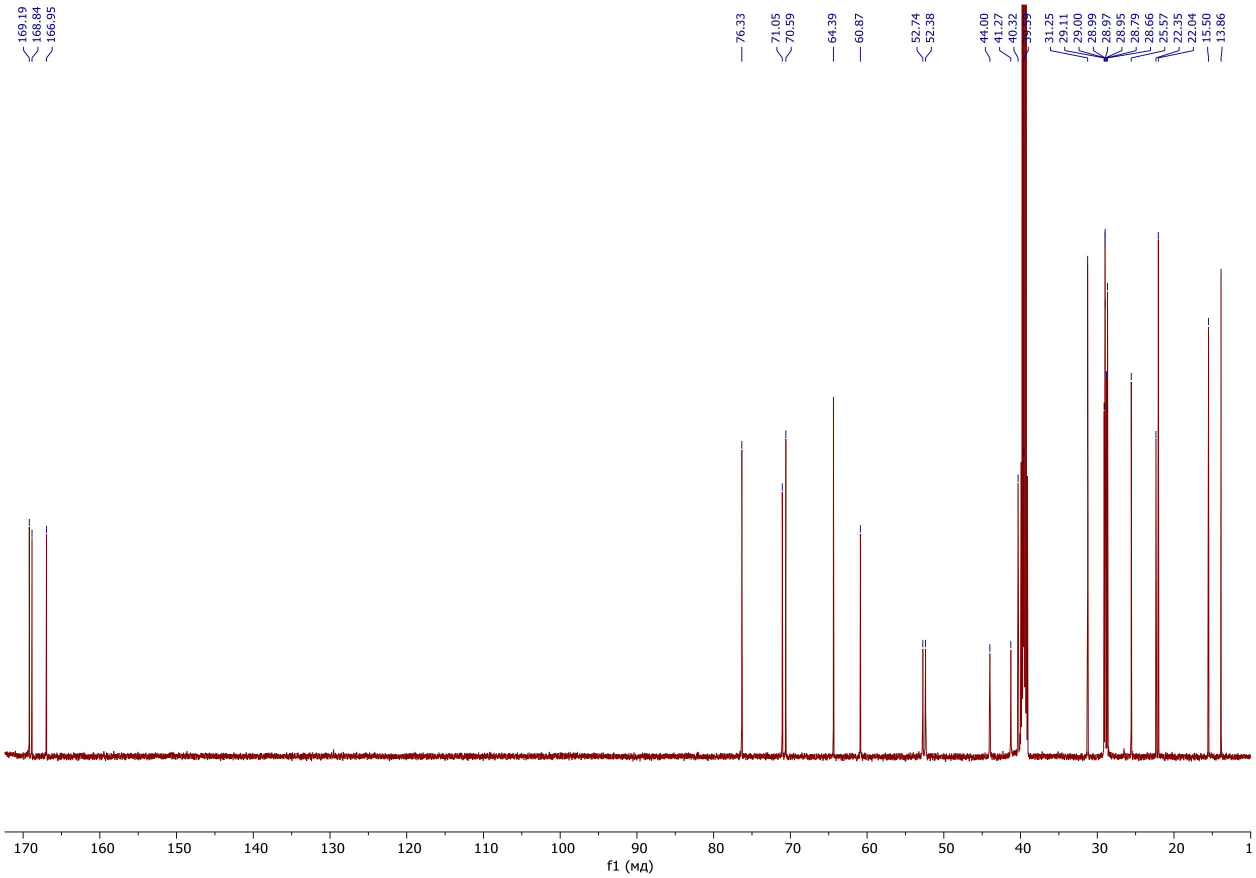

Figure S25. ^{13}C NMR spectrum of compound **12c.**

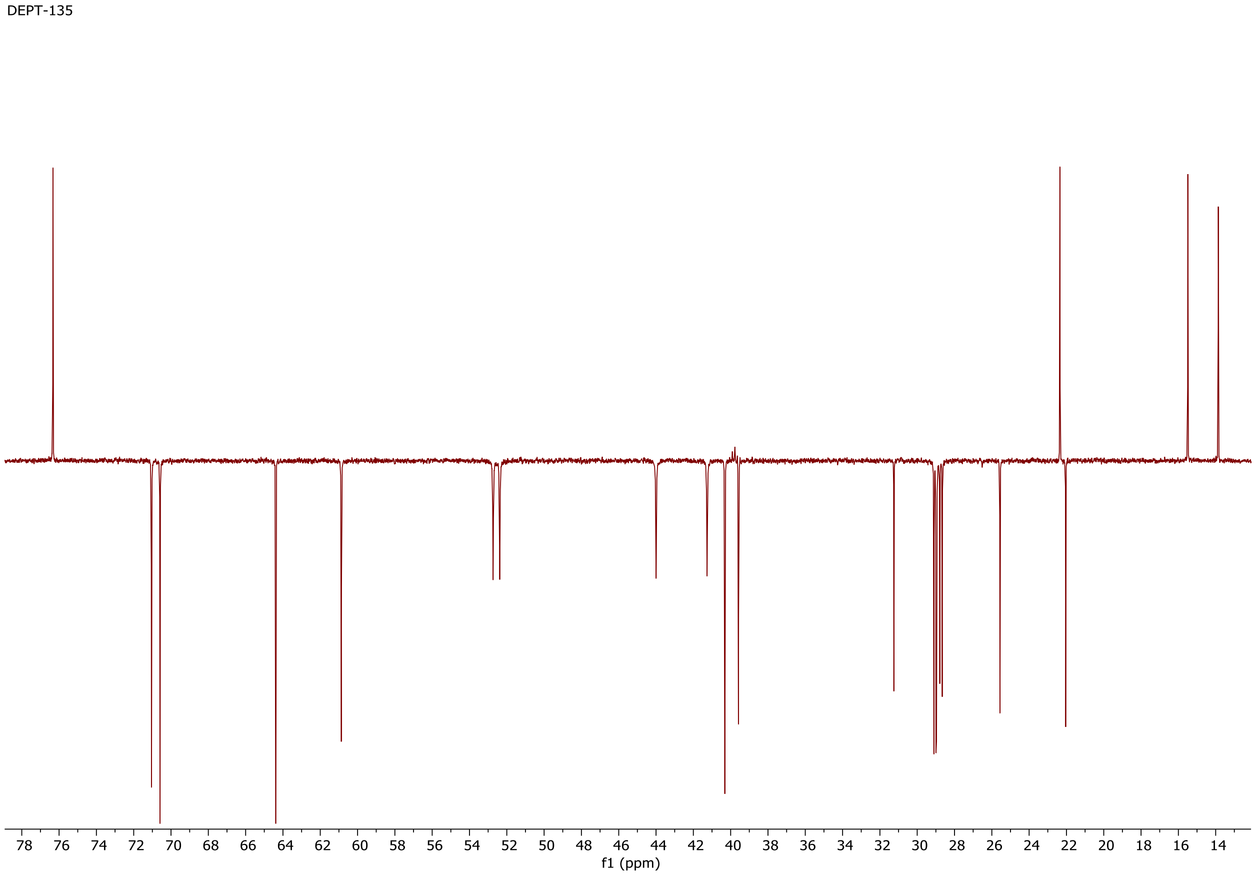

Figure S26. DEPT-135 NMR spectrum of compound **12c.**

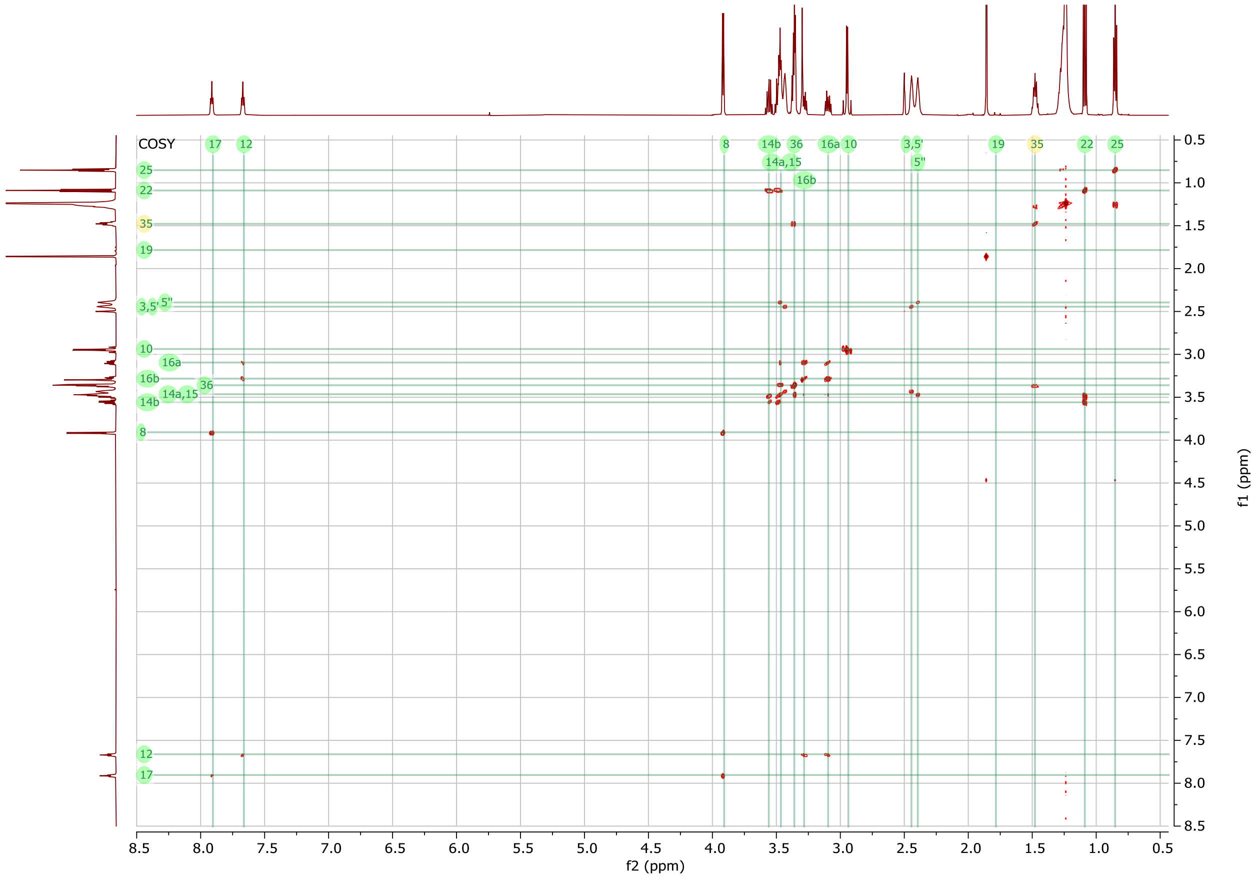

Figure S27. {¹H-¹H} COSY NMR spectrum of compound **12c.**

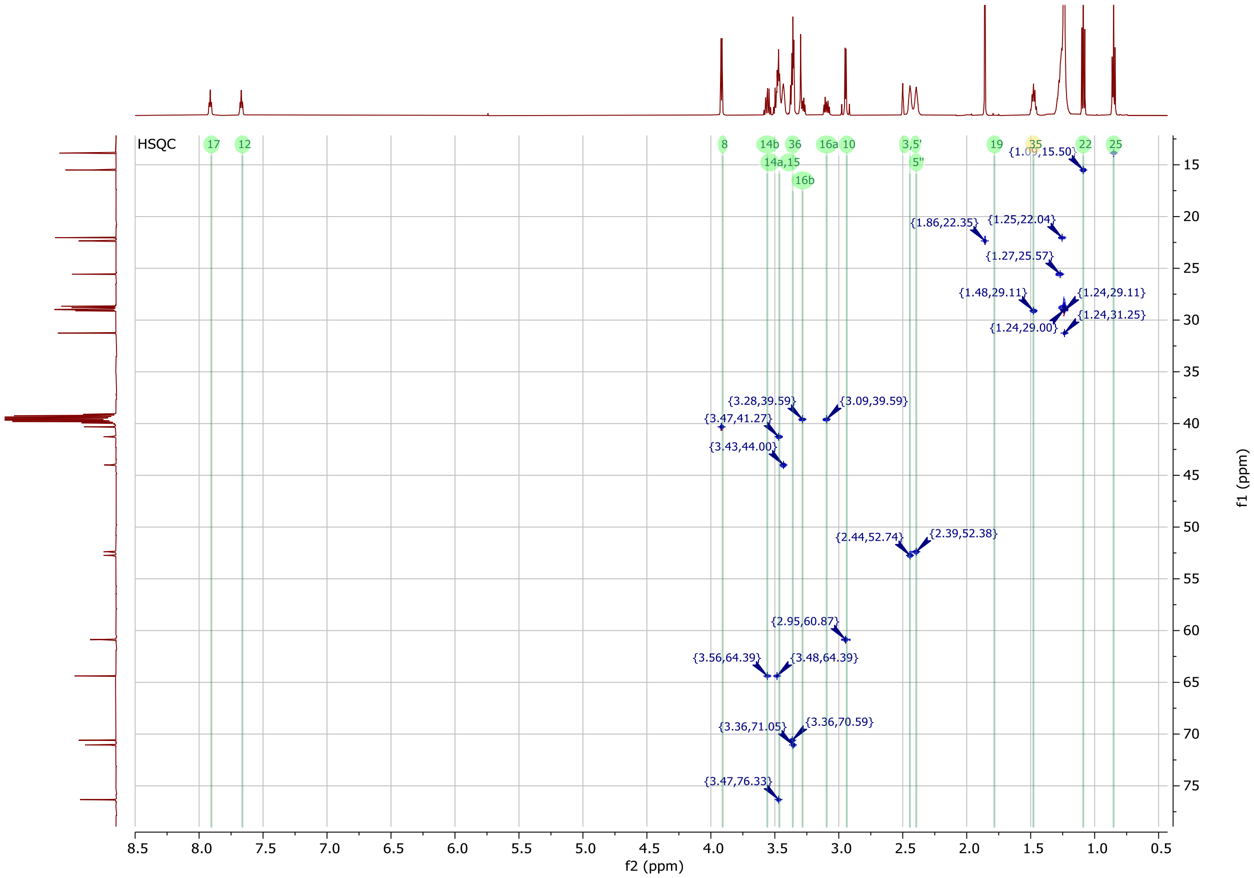

Figure S28. {¹H-¹³C} HSQC NMR spectrum of compound **12c.**

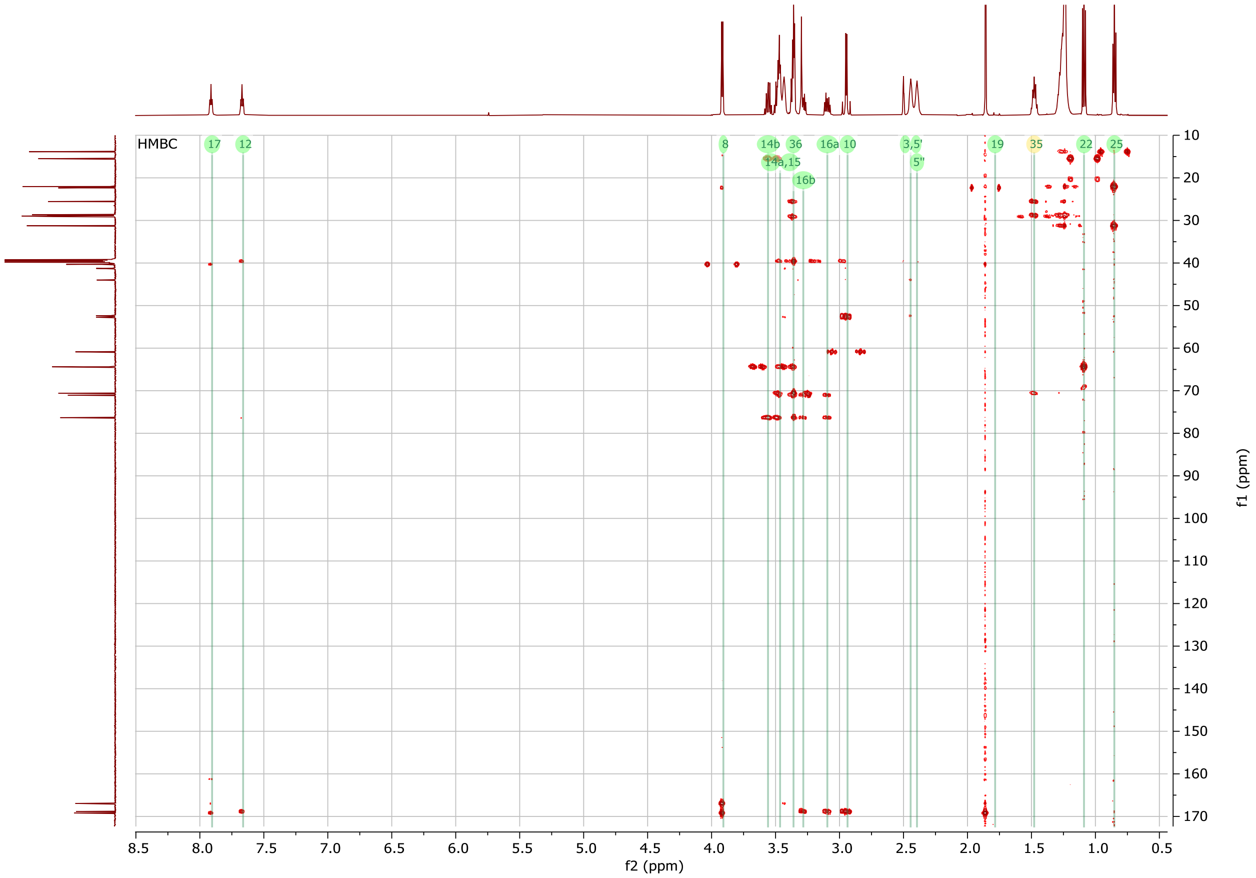

Figure S29. {^{1}H-^{13}C} HMBC NMR spectrum of compound **12c.**

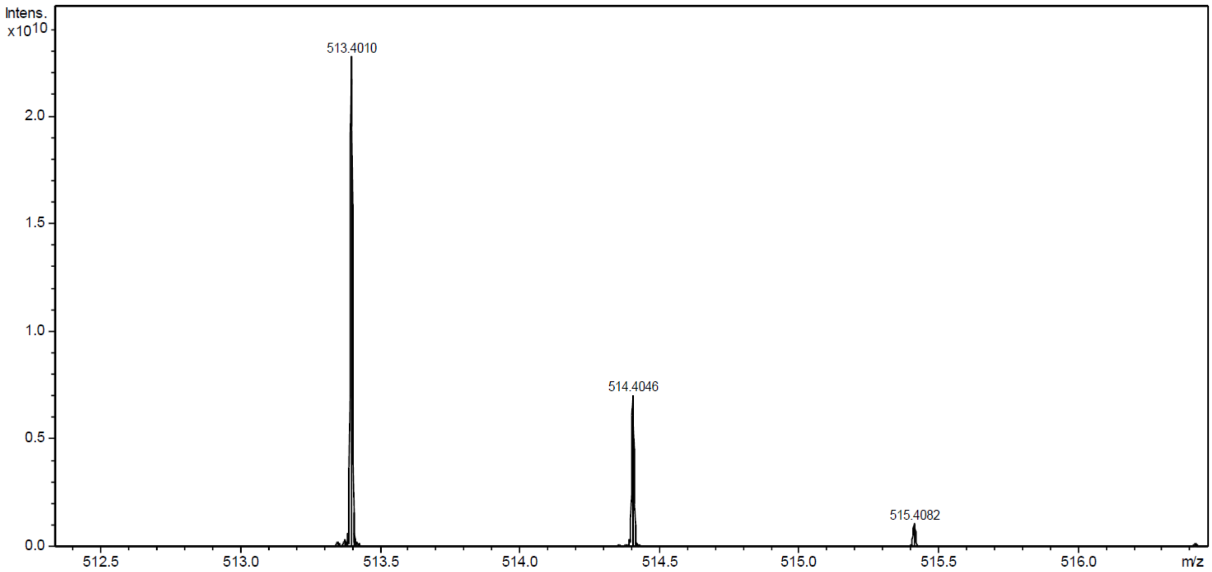

Figure S30. HRMS spectrum of compound **12c.**

N[1]-acetyl-*N*[4]-[*N*-(*rac*-1-decyloxy-2-ethyloxyprop-3-yl)aminocarbonyl]methylpiperazin (**12d**)

Yield: 54%, colorless oil. Eluent: DCM-MeOH (30:1). ^{1}H NMR (600 MHz, DMSO-d_6, HSQC, HMBC) δ 0.85 (t, 3H, *J* = 7.0 Hz, $(CH_2)_7C\underline{H}_3$), 1.09 (t, 3H, *J* = 7.0 Hz, $OCH_2C\underline{H}_3$), 1.24 (br. s, 14H, $(C\underline{H}_2)_7CH_3$), 1.44 – 1.51 (m, 2H, $OCH_2C\underline{H}_2$), 1.98 (s, 3H, $COCH_3$), 2.35 – 2.39 (m, 2H, 2 $COCH_2NC\underline{H}_eH_a$ Pip), 2.43 – 2.45 (m, 2H, 2 $COCH_2NCH_e\underline{H}_a$ Pip), 2.93 (d, 1H, *J* = 15.5 Hz, $COC\underline{H}_aH_bN$), 2.96 (d, 1H, *J* = 15.5 Hz, $COCH_a\underline{H}_bN$), 3.05 – 3.13 (m, 1H, $CONHC\underline{H}_aH_b$), 3.29 (ddd, 1H, *J* = 13.4, 6.3, 5.1 Hz, $CONHCH_a\underline{H}_b$), 3.32 – 3.39 (m, 4H, $C\underline{H}_2OC\underline{H}_2$), 3.40 – 3.52 (m, 6H, $C\underline{H}OC\underline{H}_aH_bCH_3$, 2 $CONCH_2$ Pip), 3.52 – 3.59 (m, 1H, $CHOCH_a\underline{H}_bCH_3$), 7.63 – 7.68 (m, 1H, NH). ^{13}C NMR (151 MHz, DMSO-d_6, DEPT-135) δ 13.84, 15.48, 22.04, 25.56, 28.66, 28.79, 28.95, 28.97, 28.99, 29.00 , 29.10, 31.25, 39.56, 40.72, 45.57, 52.43, 52.90, 60.89, 64.36, 64.42, 70.59, 71.06, 76.30, 168.04, 168.82. HRMS FTICR m/z: $[M+H]^+$ calcd for $C_{28}H_{55}N_4O_3$ 456.3796, found: 456.3796.

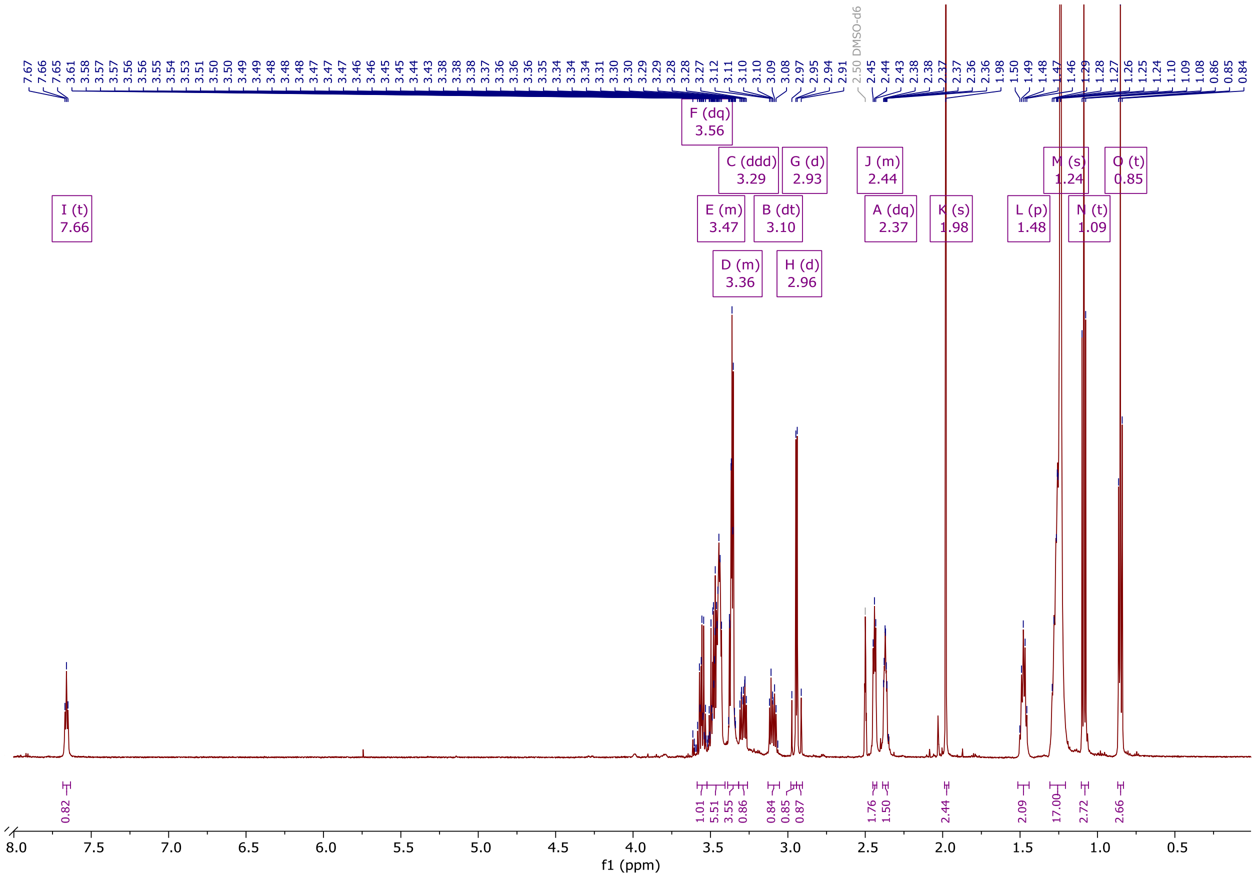

Figure S31. ^{1}H NMR spectrum of compound **12d.**

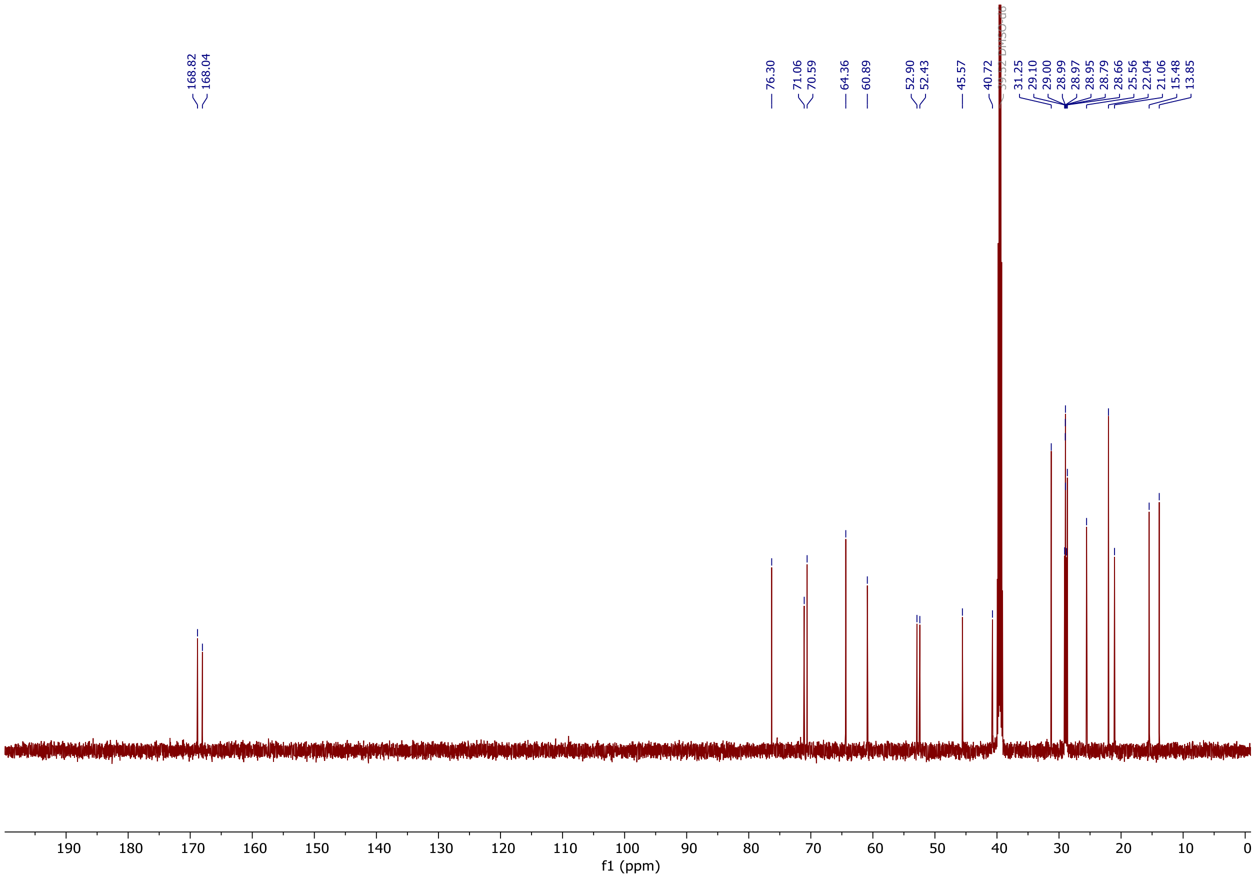

Figure S32. ^{13}C NMR spectrum of compound **12d.**

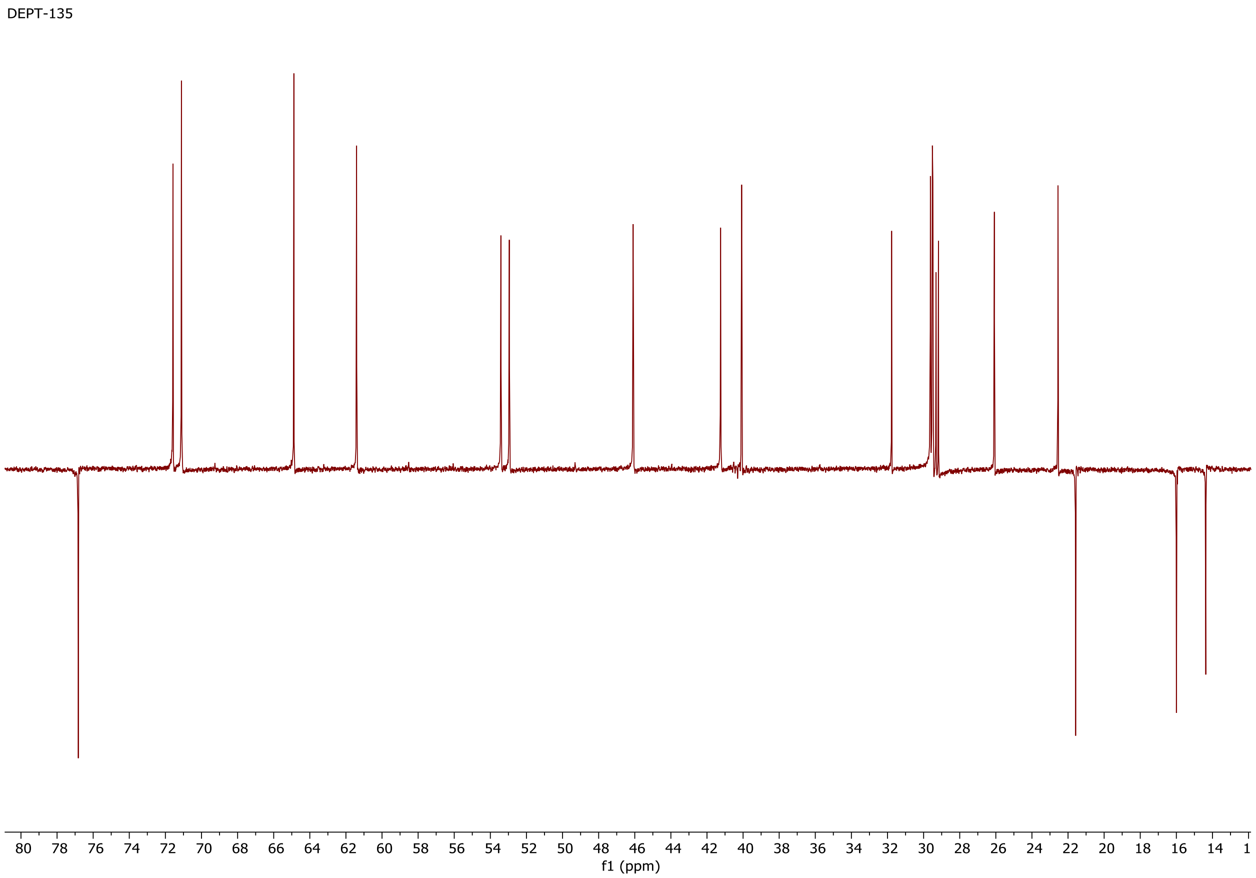

Figure S33. DEPT-135 NMR spectrum of compound **12d.**

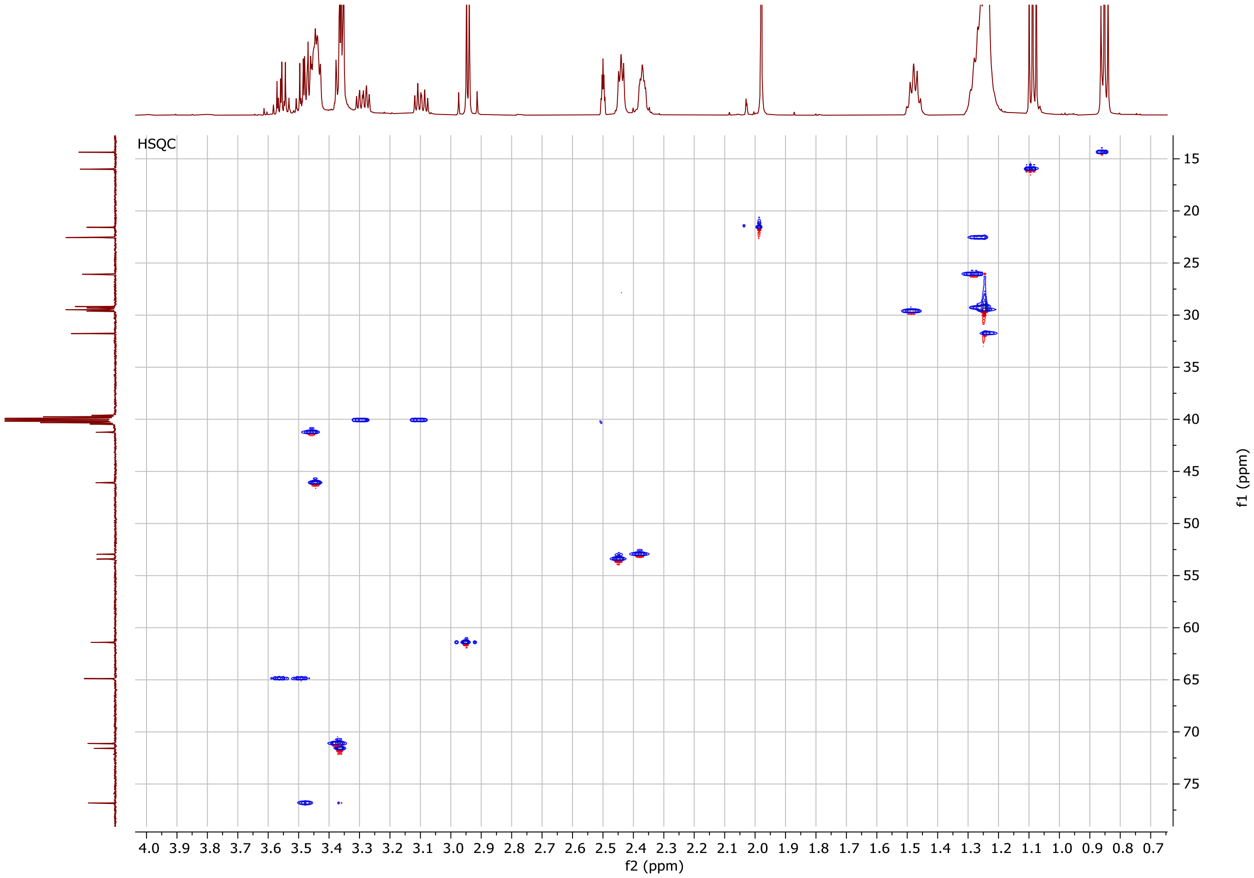

Figure S34. {¹H-¹³C} HSQC NMR spectrum of compound **12d.**

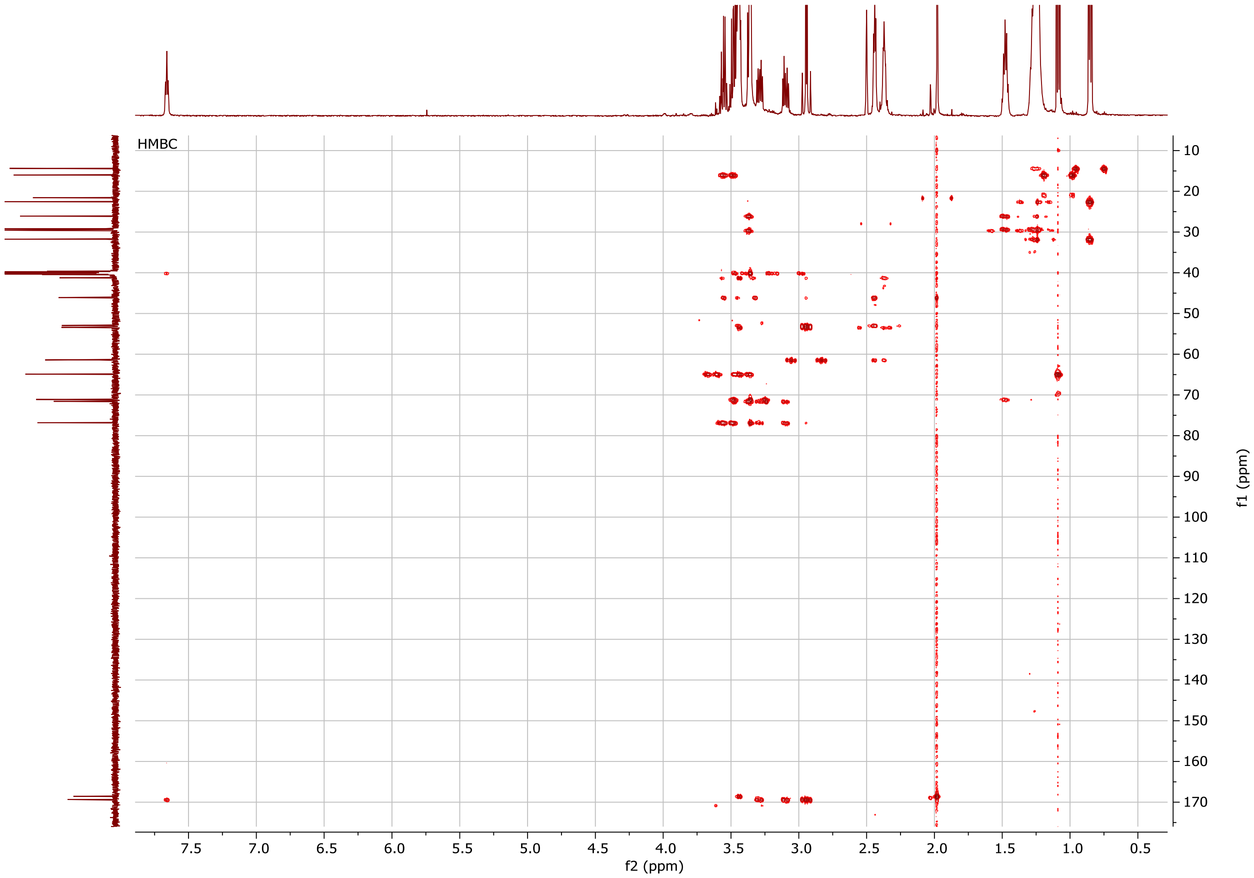

Figure S35. {¹H-¹³C} HMBC NMR spectrum of compound **12d.**

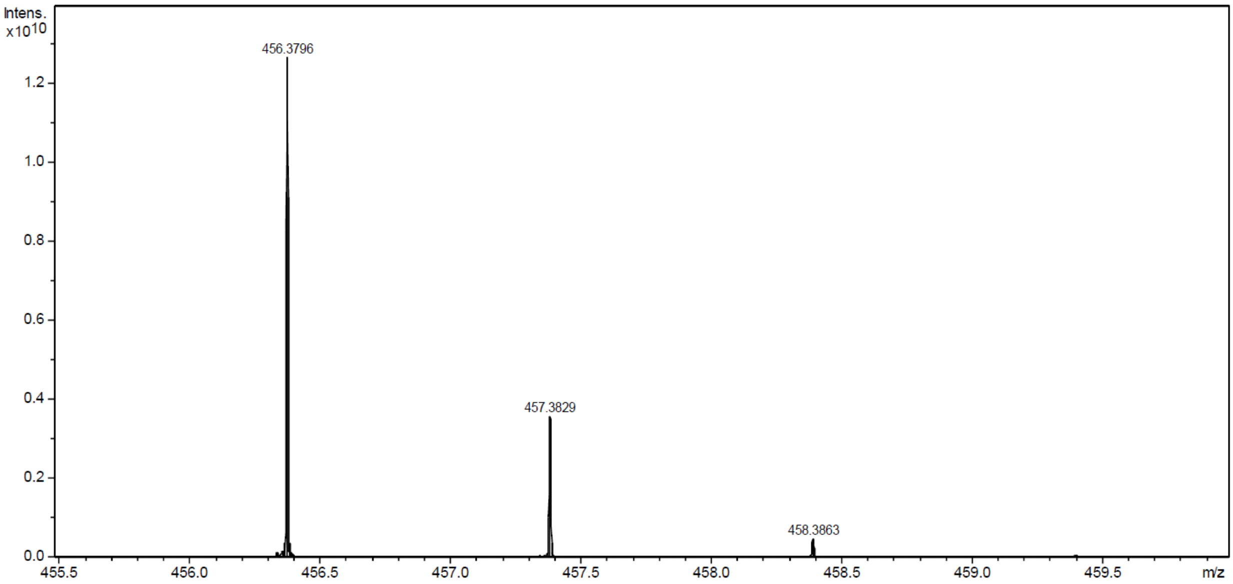

Figure S36. HRMS spectrum of compound **12d.**

N^1-acetyl-N^4-[N-(cyclohexyl)aminocarbonyl]methylpiperazin (**12e**)

Yield: 75%, colorless oil. Eluent: EA-MeOH (4:1). ^{1}H NMR (300 MHz, $CDCl_3$) δ 1.04 – 1.25 (m, 3H, 2 $CHCH_2H_e\underline{H}_a$, $CHCH_2CH_2H_e\underline{H}_a$), 1.25 – 1.45 (m, 2H, 2 $CHCH_2CH_e\underline{H}_a$), 1.61 (m, 3H, 2 $NHCHC\underline{H}_eH_a$, $CHCH_2CH_2C\underline{H}_eH_a$), 1.77 – 1.90 (m, 2H, 2 $NHCHCH_e\underline{H}_a$), 2.04 (s, 3H, $COCH_3$), 2.35 – 2.57 (m, 4H, 2 $COCH_2NC\underline{H}_2$ Pip), 2.96 (s, 2H, $COCH_2N$), 3.39 – 3.50 (m, 2H, 2 $CONC\underline{H}_eH_a$ Pip), 3.54 – 3.64 (m, 2H, 2 $CONCH_e\underline{H}_a$ Pip), 3.65 – 3.86 (m, 1H, $CONHC\underline{H}$), 6.88 (d, 1H, J = 8.2 Hz, NH). ^{13}C NMR (75 MHz, $CDCl_3$) δ 21.31, 24.73, 25.54, 33.12, 41.44, 46.28, 47.46, 53.12, 53.46, 61.60, 168.39, 169.03. HRMS ESI m/z: $[M+H]^+$ calcd for $C_{14}H_{26}N_3O_2$ 268.20195, found: 268.20195.

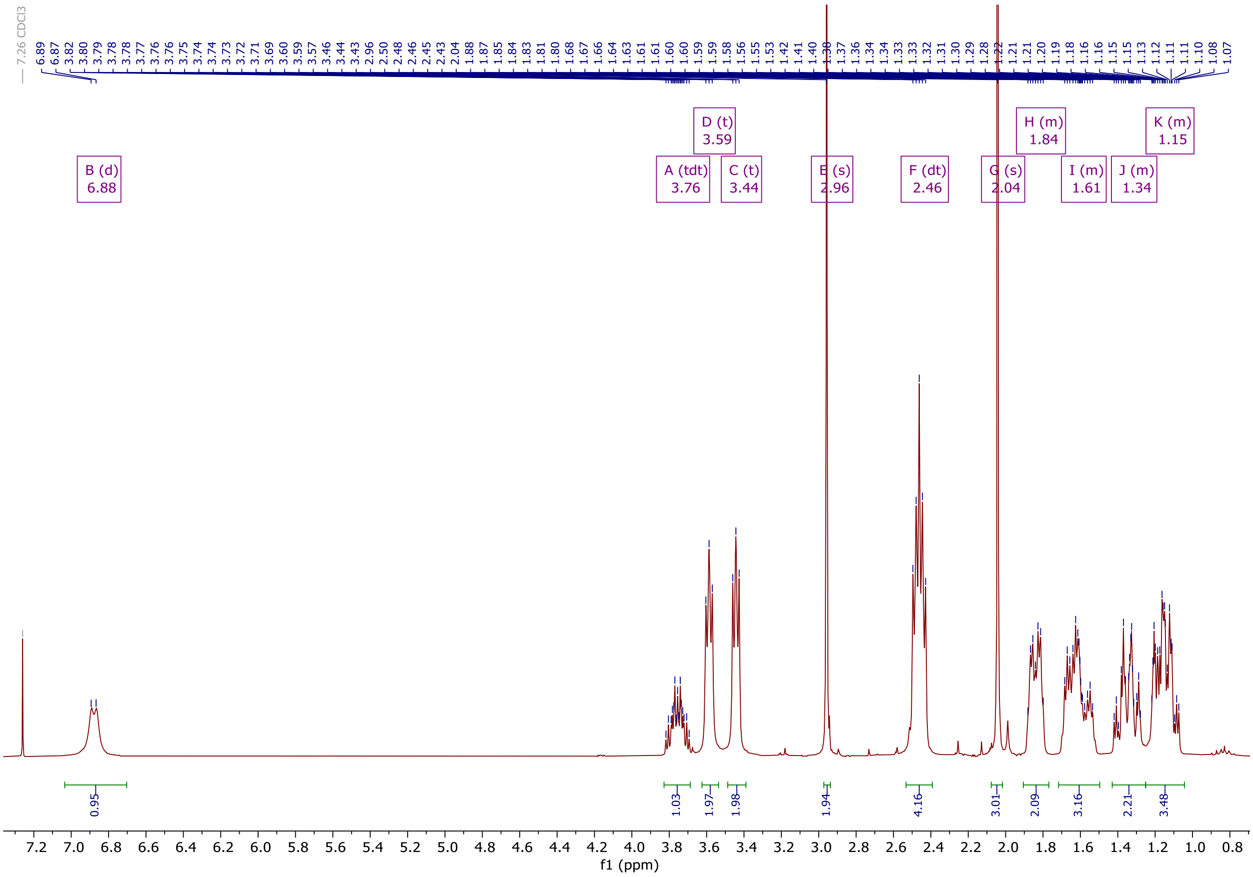

Figure S37. ^{1}H NMR spectrum of compound **12e.**

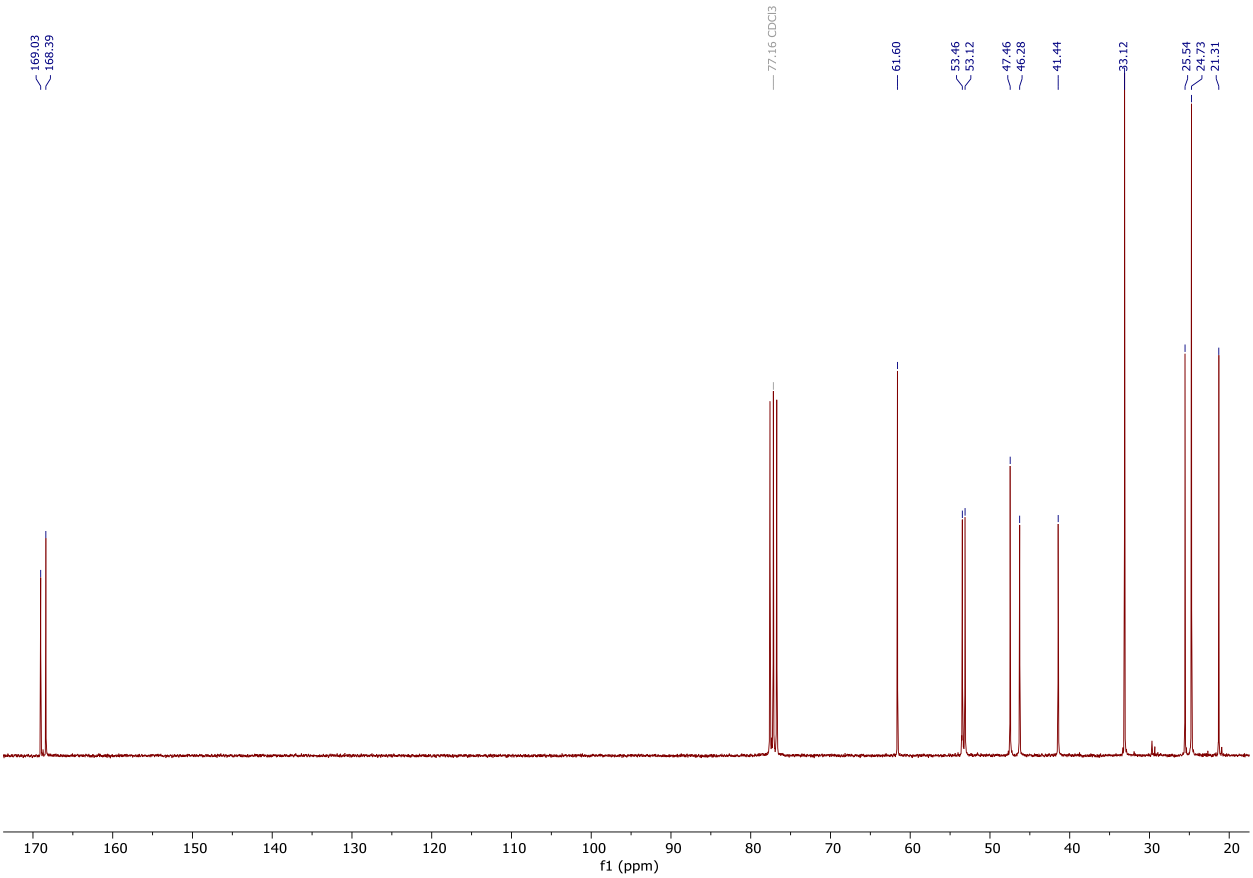

Figure S38. ^{13}C NMR spectrum of compound **12e.**

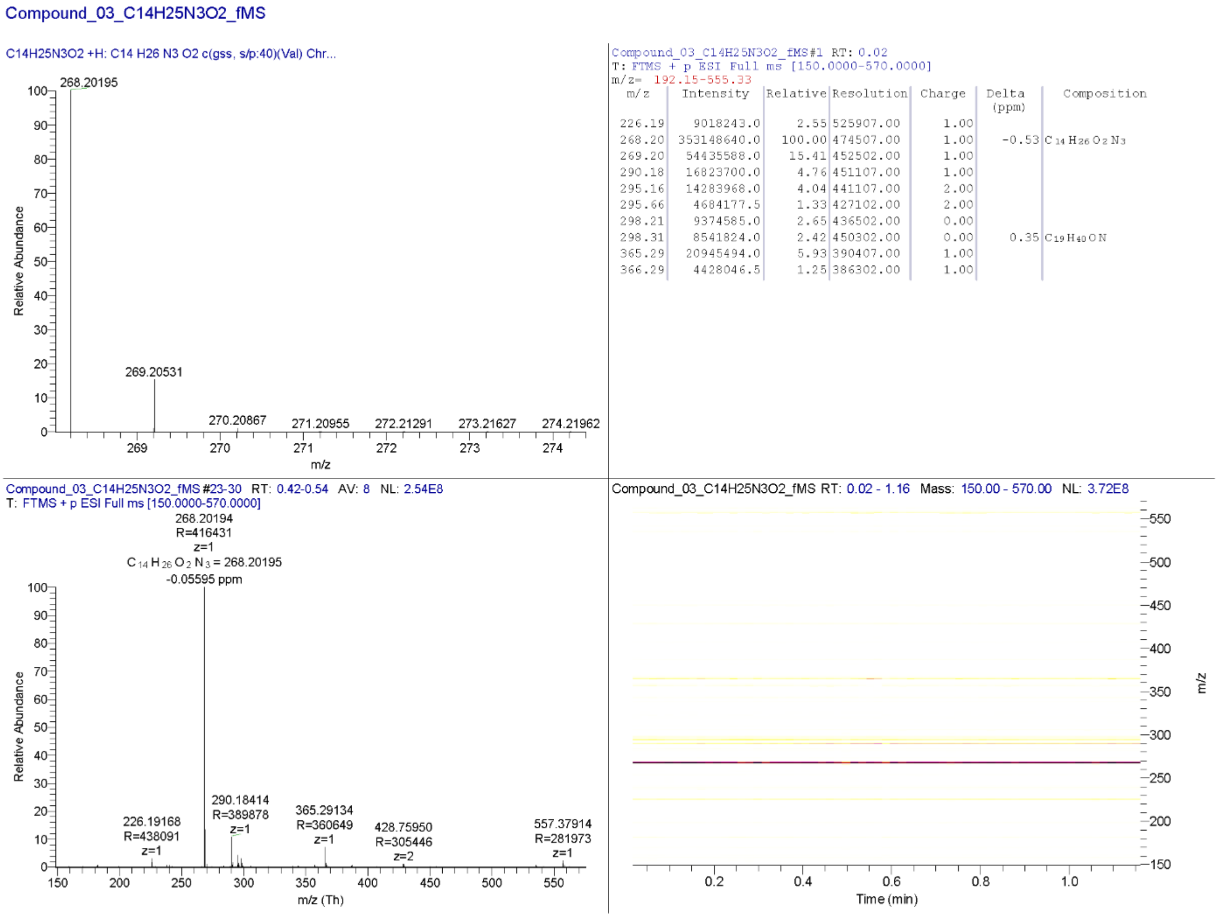

Figure S39. HRMS spectrum of compound **12e.**

N^1-[*N*-(*rac*-1-decyloxy-2-ethyloxyprop-3-yl)aminocarbonyl]methylpiperazin (**13a**)

Yield: 50%, colorless oil. Eluent: EA-MeOH-$NH_3 \cdot H_2O$ (7:3:0.1) ^{1}H NMR (300 MHz, $CDCl_3$) δ 0.87 (t, *J* = 6.3 Hz, 3H, $(CH_2)_7C\underline{H}_3$), 1.21 (t, 3H, *J* = 7.0 Hz, $OCH_2C\underline{H}_3$), 1.26 (br.s, 14H, $(C\underline{H}_2)_7CH_3$), 1.48 – 1.63 (m, 2H, $OCH_2C\underline{H}_2$), 2.43 – 2.63 (m, 5H, CH_2NHCH_2 Pip), 2.87 – 2.96 (m, 4H, 2 NCH_2 Pip), 2.99 (s, 2H, $COCH_2N$), 3.17 – 3.75 (m, 9H, $C\underline{H}_2OC\underline{H}_2$, $C\underline{H}OC\underline{H}_2CH_3$, $C\underline{H}_2NHCO$), 7.50 (br.s, 1H, CONH). ^{13}C NMR (75 MHz, $CDCl_3$) δ 14.26, 15.80, 22.82, 26.23, 29.46, 29.61, 29.72, 29.75, 40.23, 46.13, 54.71, 62.33, 65.53, 71.51, 72.01, 76.90, 170.30. HRMS ESI [M+H]$^+$ calcd for $C_{21}H_{44}N_3O_3$ 386.3377, found 386.3371.

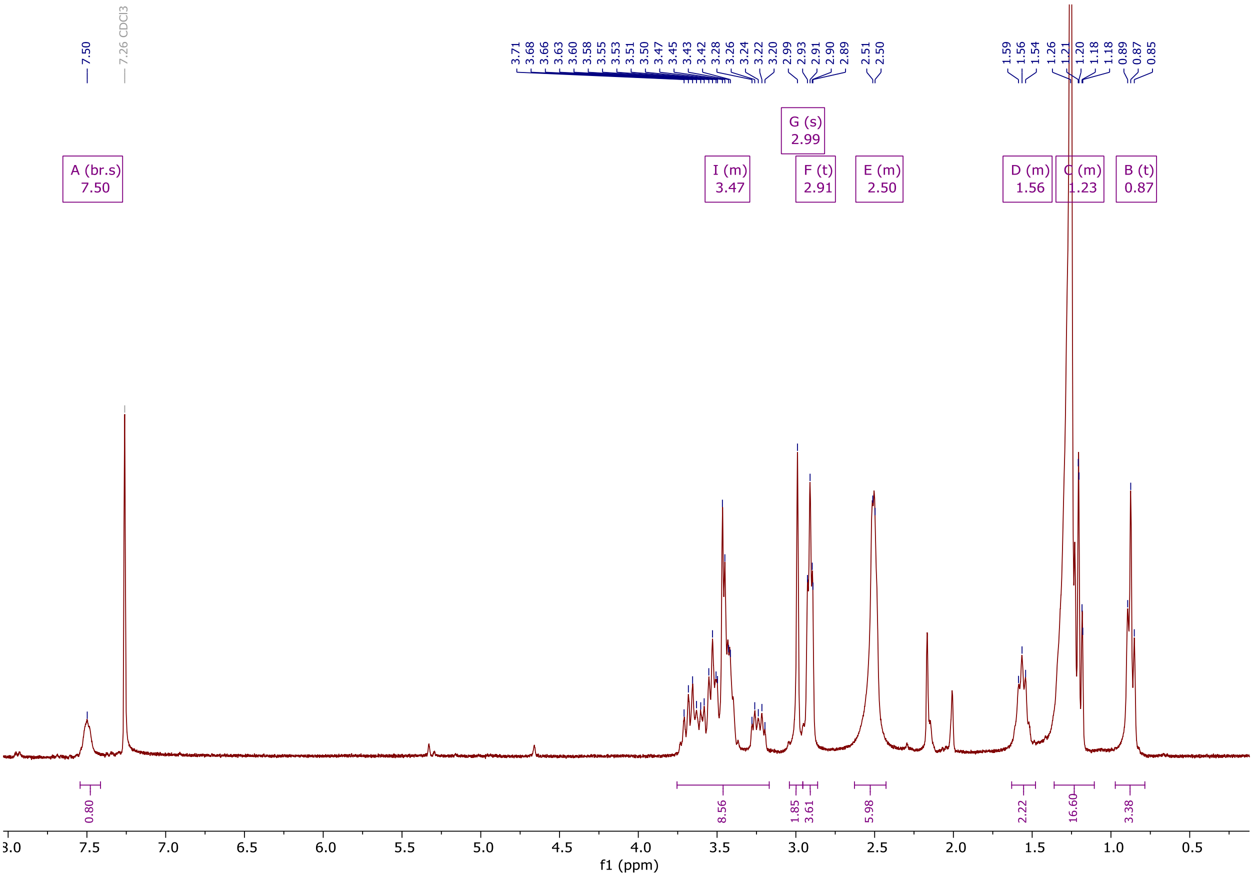

Figure S40. ^{1}H NMR spectrum of compound **13a.**

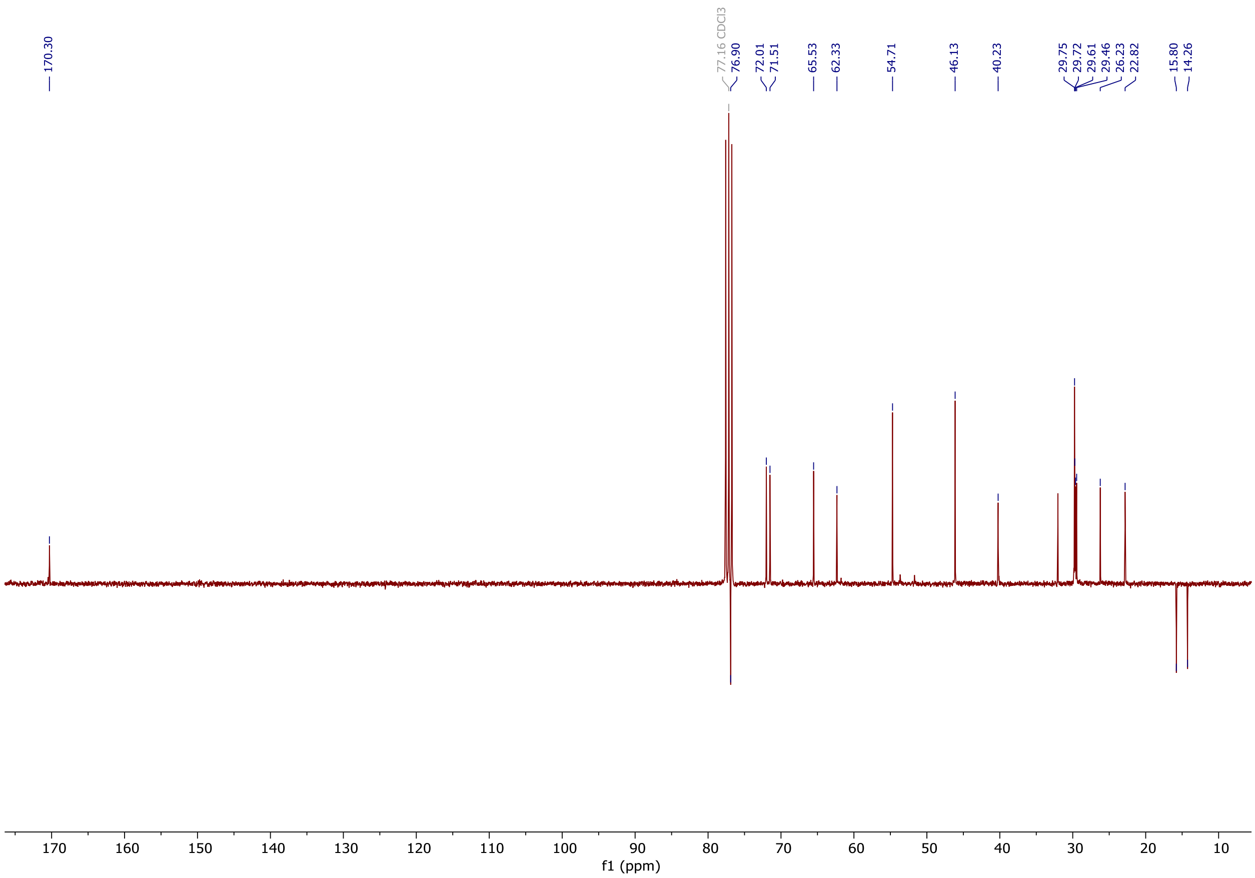

Figure S41. ^{13}C (APT)C NMR spectrum of compound **13a.**

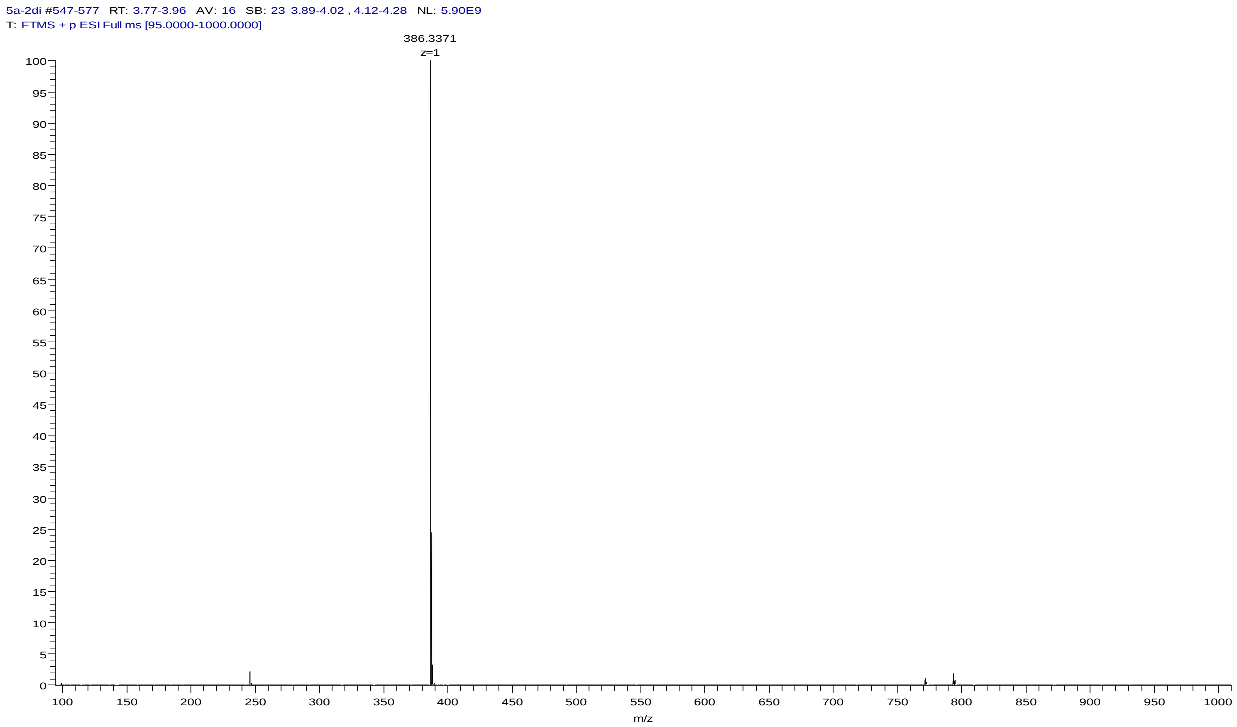

Figure S42. {^{1}H-^{13}C} HMBC NMR spectrum of compound **13a.**

N[1],*N*[4]-bis[*N*-(*rac*-1-decyloxy-2-ethyloxyprop-3-yl)aminocarbonyl]methylpiperazin (**13b**)

Yield: 22%, colorless oil. Eluent: EA-MeOH (95:5). ^{1}H NMR (300 MHz, $CDCl_3$) δ 0.87 (t, 6H, *J* = 6.5 Hz, $(CH_2)_7C\underline{H}_3$), 1.19 (t, 6H, *J* = 7.0 Hz, $OCH_2C\underline{H}_3$), 1.27 (br.s, 28H,

$(C\underline{H}_2)_7CH_3$), 1.50 – 1.63 (m, 4H, $OCH_2C\underline{H}_2$), 2.56 (br.s, 8H Pip), 3.01 (s, 4H, $COCH_2N$), 3.16 – 3.31 (m, 2H, 2 $C\underline{H}_aH_bNHCO$), 3.34 – 3.48 (m, 8H, 2 $C\underline{H}OC\underline{H}_2CH_3$, 2 $CH_a\underline{H}_bNHCO$), 3.48 – 3.56 (m, 4H, 2 $C\underline{H}_2OC\underline{H}_2$), 3.56 – 3.74 (m, 4H, 2 $C\underline{H}_2OCH_2$). ^{13}C NMR (75 MHz, $CDCl_3$) δ 14.26, 15.83, 22.82, 26.23, 29.46, 29.62, 29.71, 29.75, 29.77, 32.04, 40.26, 53.75, 61.67, 65.49, 71.51, 72.01, 76.81, 170.05. HRMS ESI $[M+H]^+$calcd for $C_{38}H_{77}N_4O_6$ 685.5838, found 685.5828. HRMS ESI $[M+2H]^{2+}$calcd for $C_{38}H_{78}N_4O_6$ 343.2955, found 343.2954.

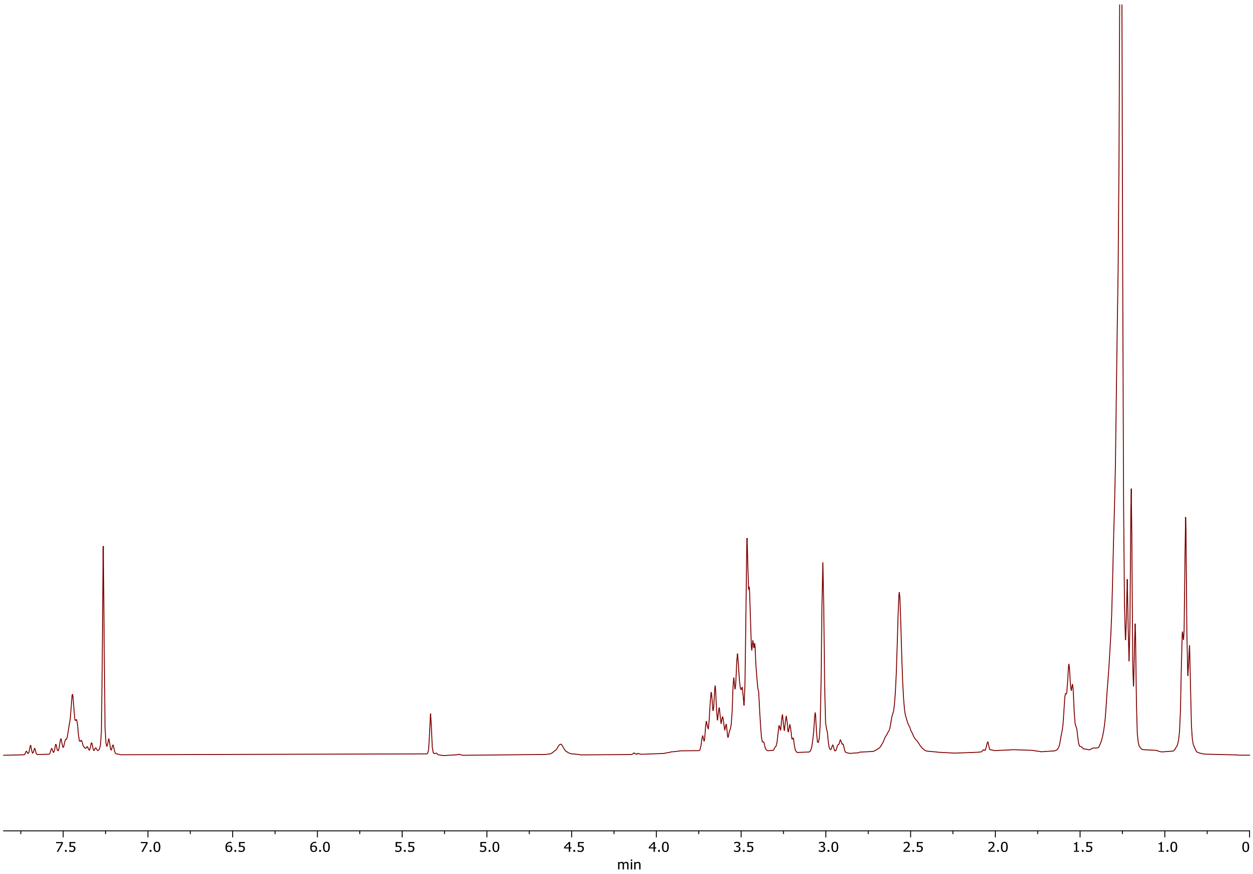

Figure S43. ^1H NMR spectrum of compound **13b.**

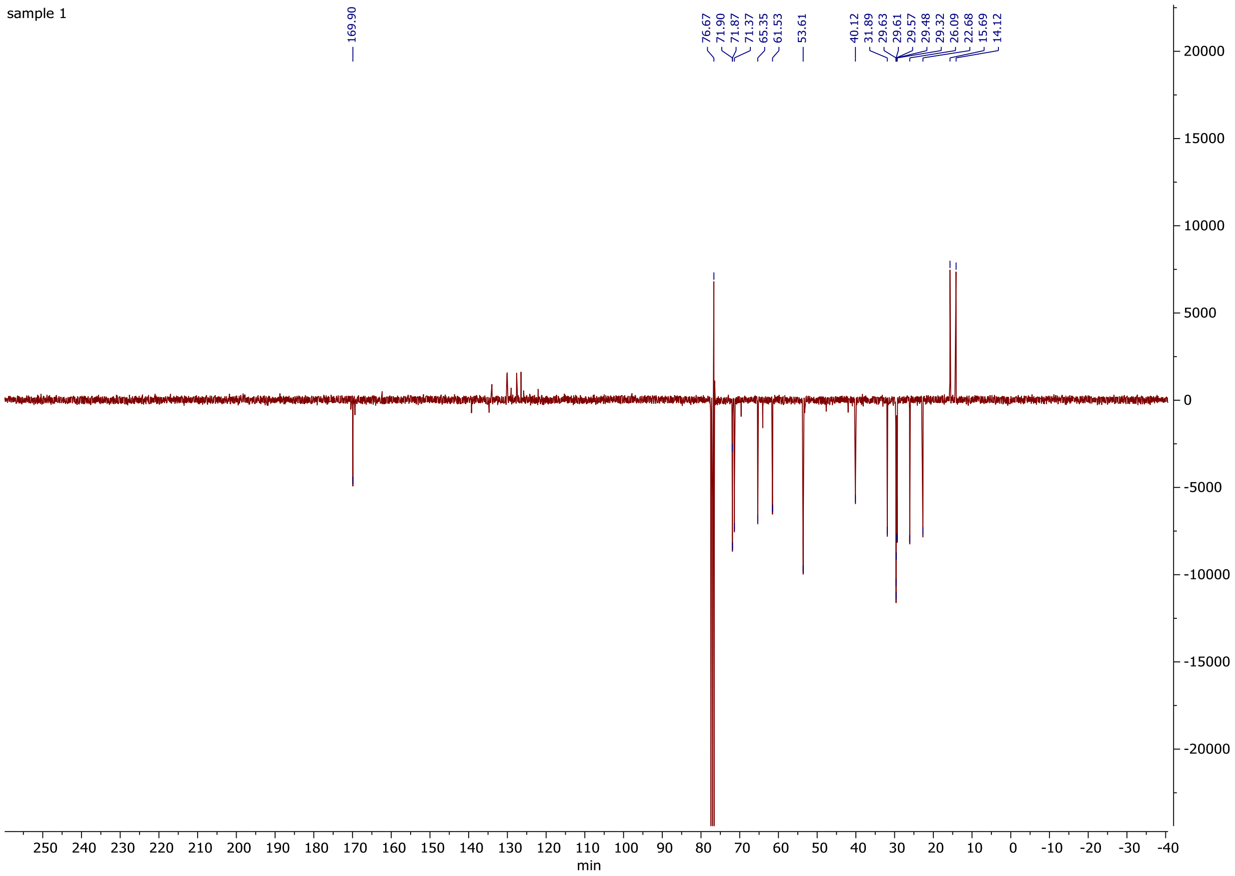

Figure S44. ^{13}C (APT) NMR spectrum of compound **13b.**

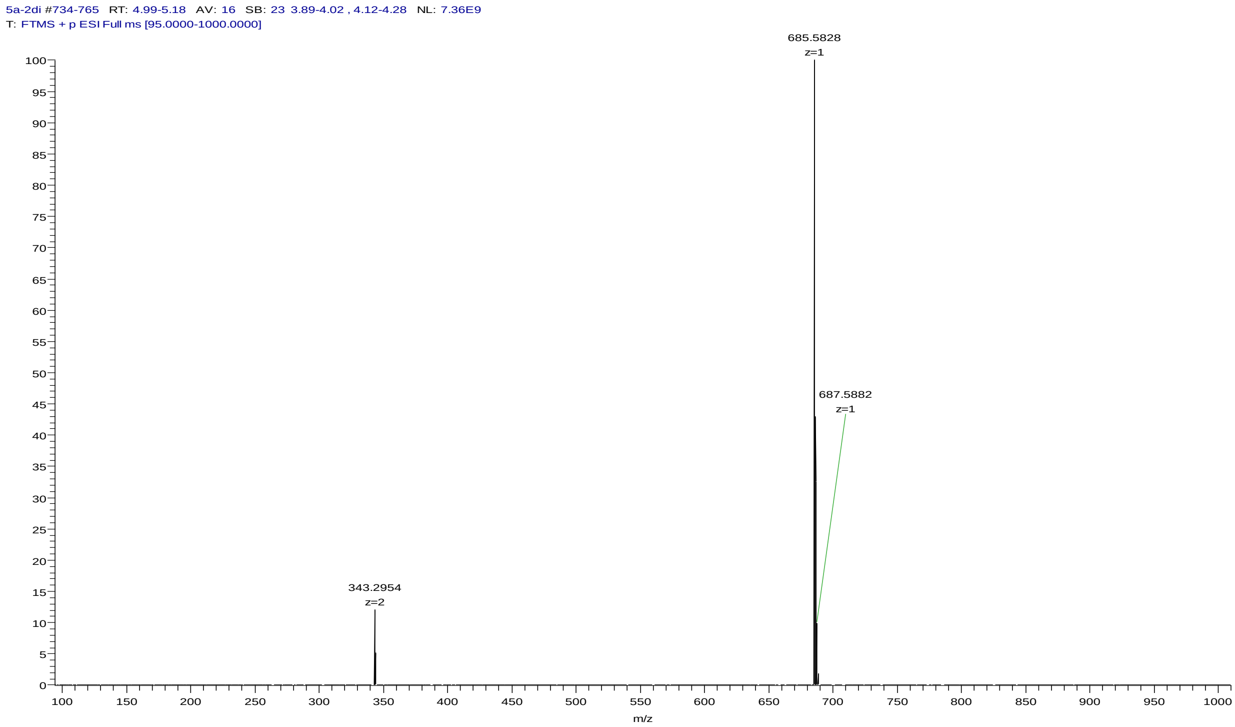

Figure S45. HMRS spectrum of compound **13b.**

N[1]-[*N*-(isopropyl)aminocarbonyl]methyl-*N*[4]-[*N*-(*rac*-1-decyloxy-2-ethyloxyprop-3-yl)aminocarbonyl]methylpiperazin (**15a**)

Yield: 80%, colorless oil. Eluent: EA-MeOH (85:15). ^{1}H NMR (300 MHz, $CDCl_3$) δ 0.0.87 (t, *J* = 7.0 Hz, 3H, $(CH_2)_7C\underline{H}_3$), 1.16 (d, *J* = 6.6 Hz, 6H, $CH(C\underline{H}_3)_2$), 1.20 (t, *J* = 7.0 Hz, 3H, $OCH_2C\underline{H}_3$), 1.27 (br.s, 14H, $(C\underline{H}_2)_7CH_3$), 1.50 – 1.62 (m, 2H, $OCH_2C\underline{H}_2$), 2.56 (br.s, 8H Pip protons), 2.97 (s, 2H, $CHNHC(O)C\underline{H}_2$), 3.03 (d, *J* = 1.6 Hz, 2H, $COCH_2N$), 3.23 (ddd, *J* = 4.8, 6.4, 13.7 Hz, 1H, $CHC\underline{H}_aH_bNH$), 3.34 – 3.75 (m, 8H, $CHCH_a\underline{H}_bNH$, $C\underline{H}OC\underline{H}_2CH_3$, $C\underline{H}_2OC\underline{H}_2$), 4.01 – 4.16 (m, 1H, $C\underline{H}(CH_3)_2$), 6.86 (br.d, *J* = 8.4 Hz, 1H, $N\underline{H}CH$), 7.44 (br.t, *J* = 5.6 Hz, 1H, $N\underline{H}CH_2$). ^{13}C NMR (75 MHz, $CDCl_3$) δ 14.26, 15.83, 22.83, 22.96, 26.25, 29.47, 29.63, 29.72, 29.76, 29.78, 32.04, 40.27, 40.87, 53.69, 53.74, 61.65, 61.72, 65.49, 71.54, 72.03, 76.82, 168.98, 170.03. HRMS ESI $[M+H]^+$ calcd for $C_{26}H_{53}N_4O_4$ 485.4061, found 485.4062.

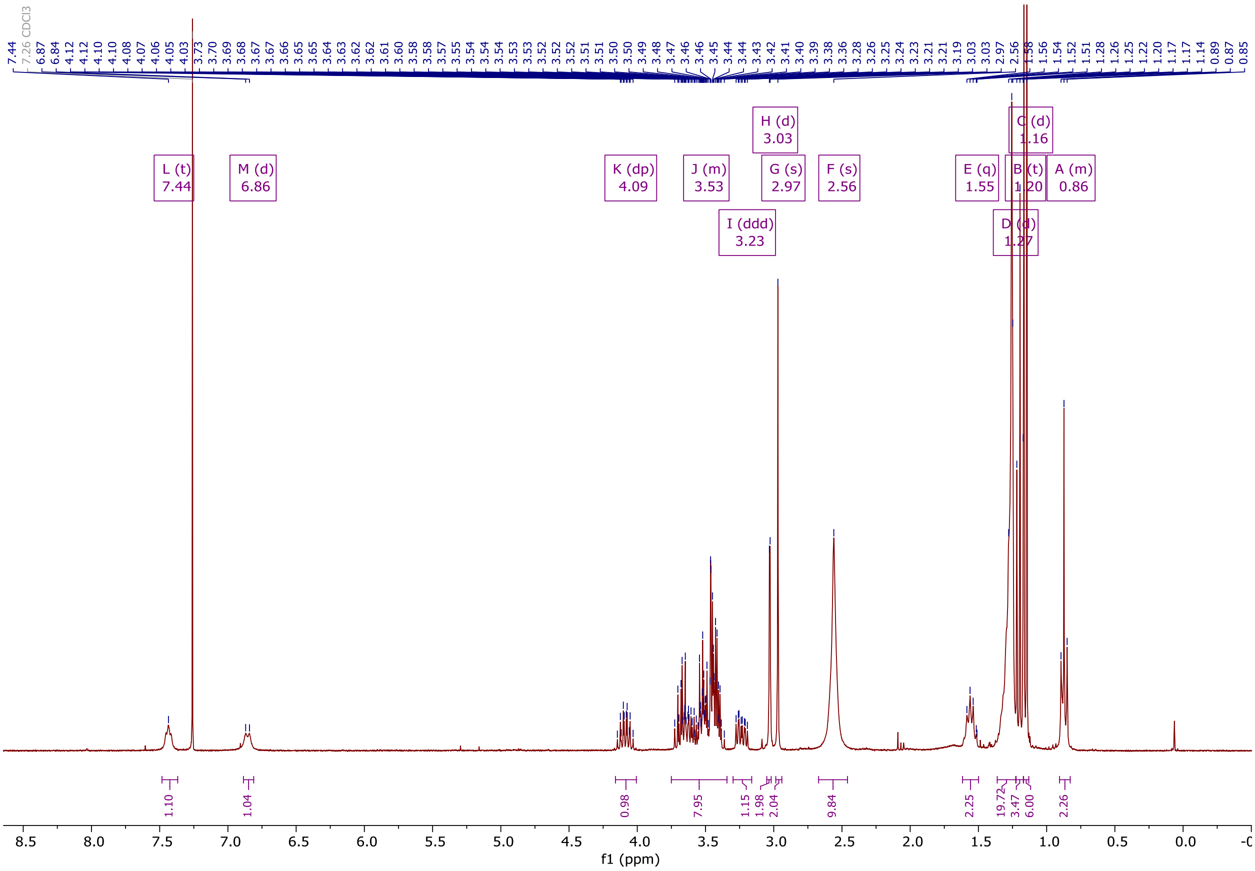

Figure S46. ^{1}H NMR spectrum of compound **15a.**

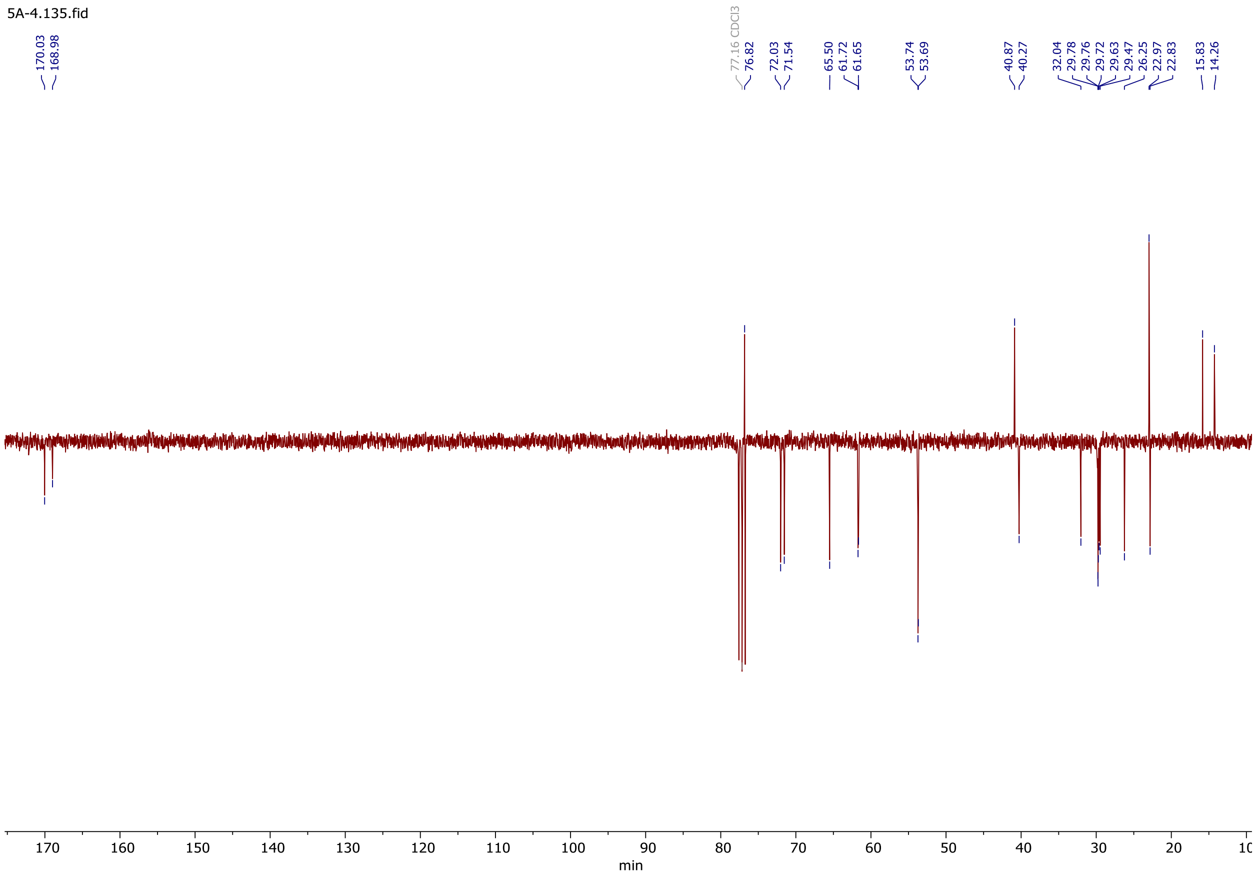

Figure S47. ^{13}C NMR spectrum of compound **15a.**

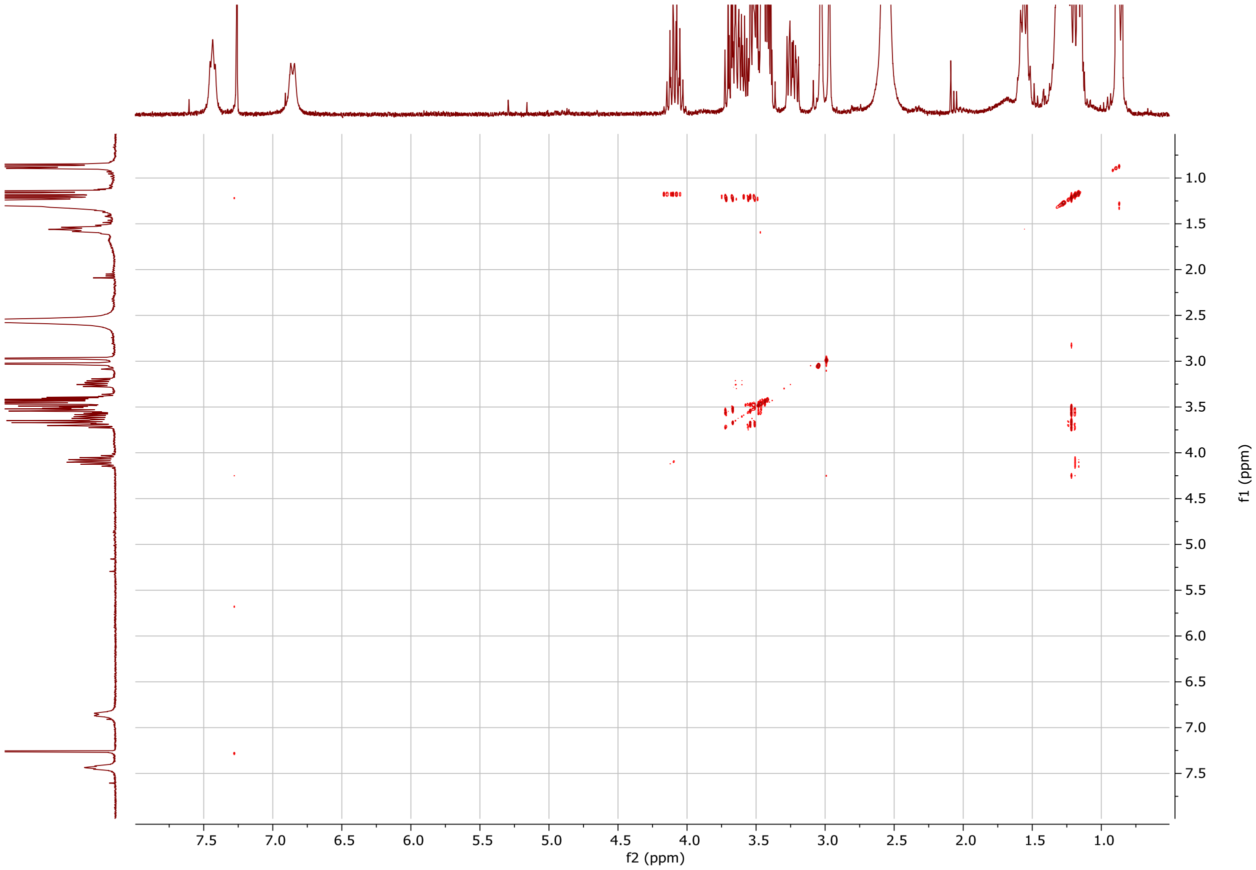

Figure S48. {^{1}H-^{1}H} COSY NMR spectrum of compound **15a.**

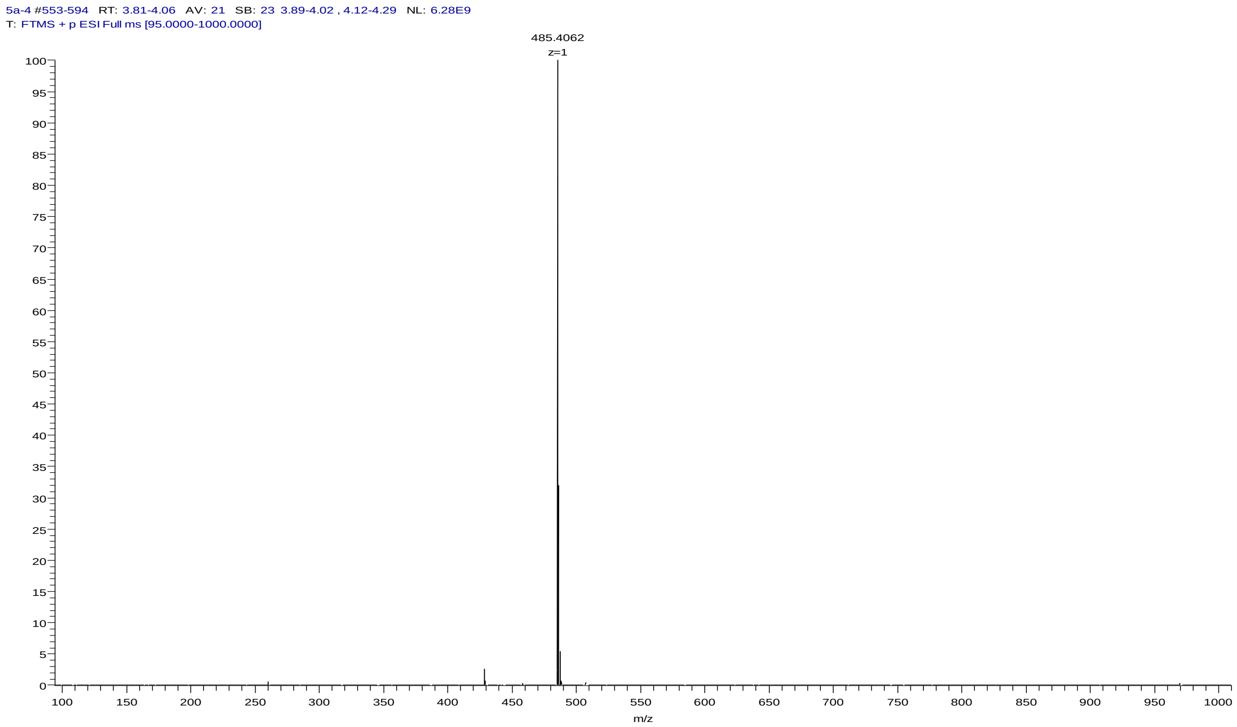

Figure S49. HRMS spectrum of compound **15a.**

N[1]-[*N*-(pentyl)aminocarbonyl]methyl-*N*[4]-[*N*-(*rac*-(1-decyloxy-2-ethyloxyprop-3-yl)aminocarbonyl]methylpiperazin (**15b**)

Yield: 75%, colorless oil. Eluent: EA-MeOH (9:1). ^1H NMR (400 MHz, $CDCl_3$, COSY, HSQC, HMBC) δ 0.83 – 0.94 (m, 6H, $(CH_2)_7C\underline{H}_3$, $(CH_2)_4C\underline{H}_3$), 1.20 (t, 3H, *J* = 7.0 Hz, $OCH_2C\underline{H}_3$), 1.23 – 1.39 (br. s, 18H, $(C\underline{H}_2)_7CH_3$, $NHCH_2CH_2(C\underline{H}_2)_2CH_3$), 1.46 – 1.63 (m, 4H, $NHCH_2C\underline{H}_2$, $OCH_2C\underline{H}_2$), 2.42 – 2.70 (br.s, 8H, Pip protons), 2.96 – 3.07 (m, 4H, 2 $COCH_2N$), 3.18 – 3.32 (m, 3H, $CHOC\underline{H}_aH_bN$, $NHC\underline{H}_2CH_2$), 3.37 – 3.59 (m, 6H, 2 $C\underline{H}_2OC\underline{H}_2$, $OC\underline{H}_aH_bCH_3$, CHO), 3.59 – 3.76 (m, 2H, $OCH_a\underline{H}_bCH_3$, $CHOCH_a\underline{H}_bN$), 7.09 (br. s, 1H, NH), 7.45 (bras, 1H, NH). ^{13}C NMR (101 MHz, $CDCl_3$) δ 14.12, 14.22, 15.79, 22.43, 22.78, 26.20, 29.22, 29.43, 29.45, 29.58, 29.68, 29.720, 29.74, 32.00, 39.01, 40.23, 53.63, 53.72, 61.59, 61.61, 65.44, 71.48, 71.98, 76.75, 169.74, 169.96. MS ESI m/z: $[M+H]^+$ calcd for $C_{28}H_{57}N_4O_4$ 513.44, found: 513.50.

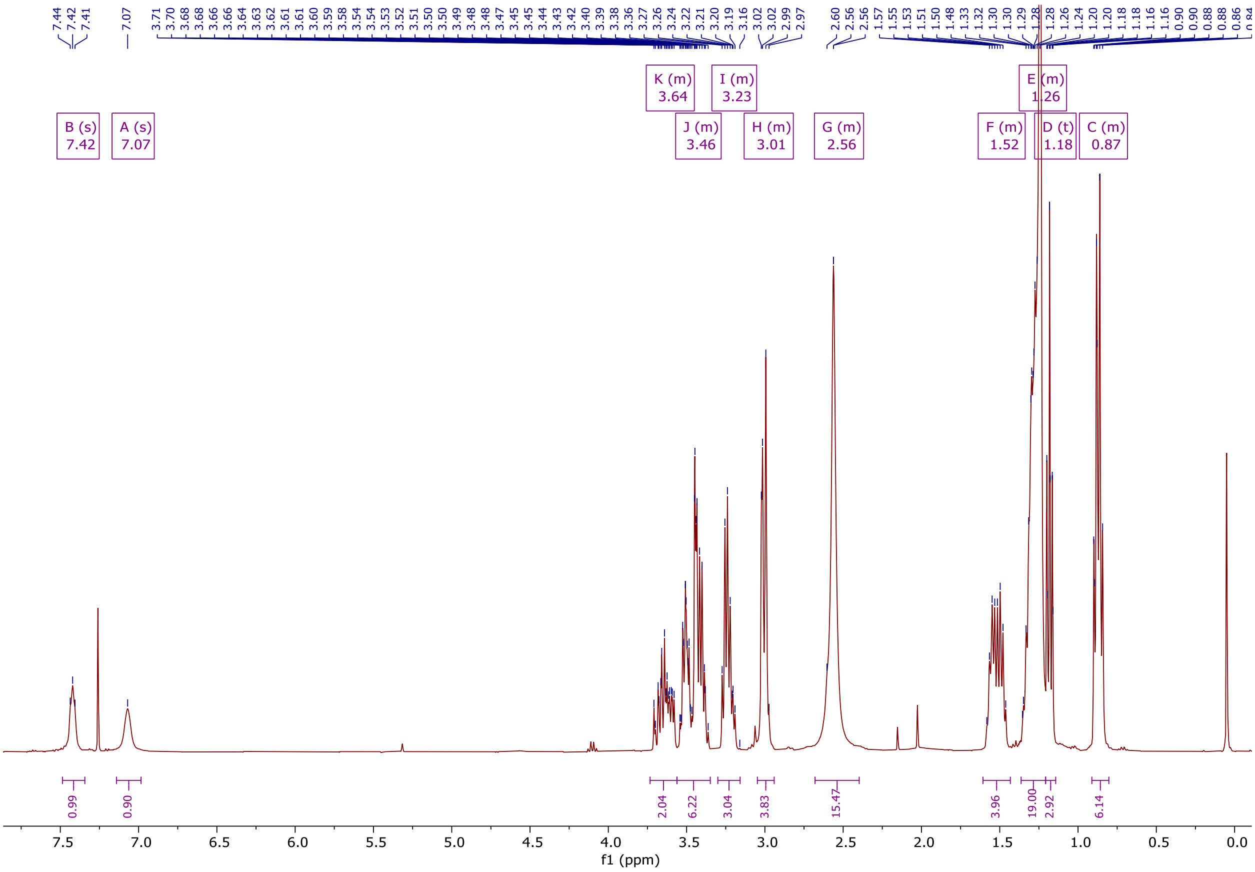

Figure S50. ^1H NMR spectrum of compound **15b.**

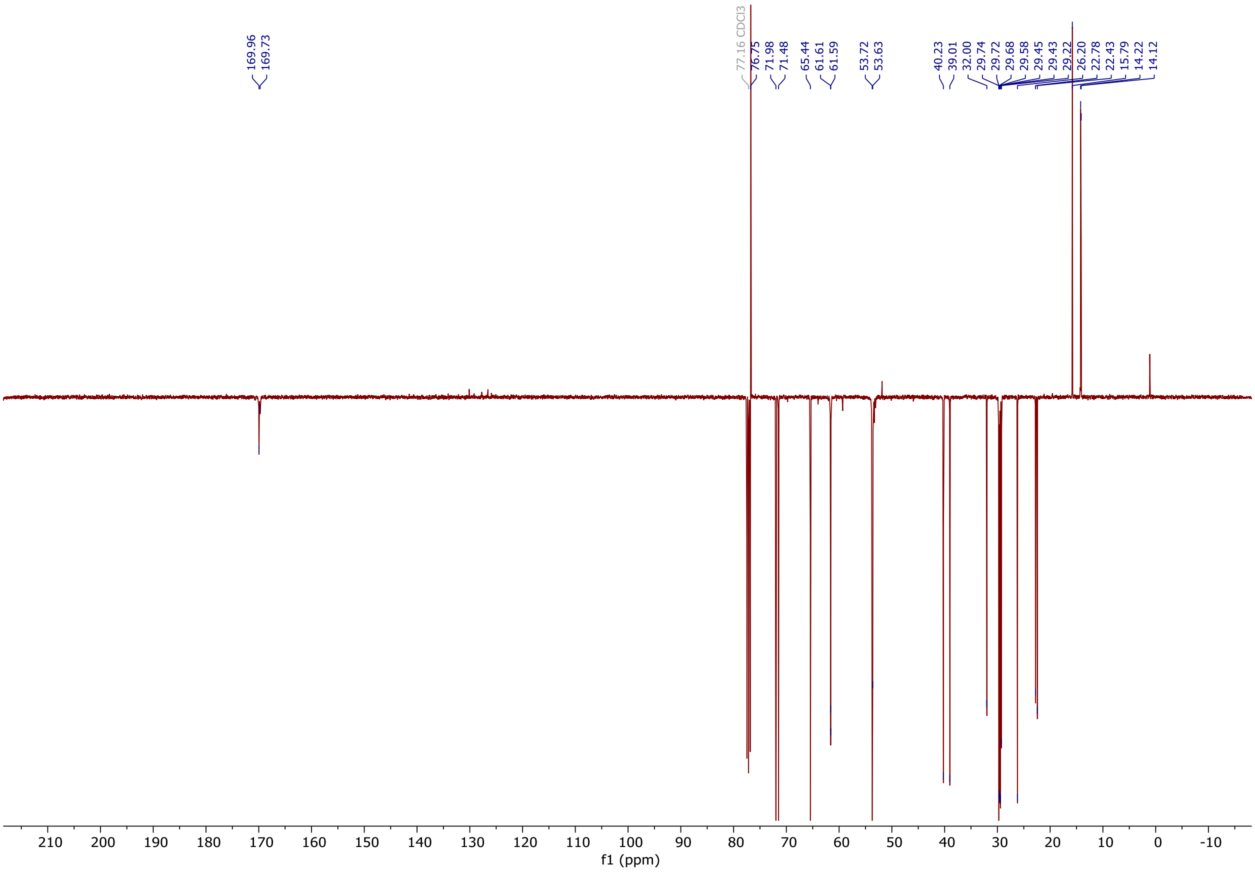

Figure S51. ^{13}C (APT) NMR spectrum of compound **15b.**

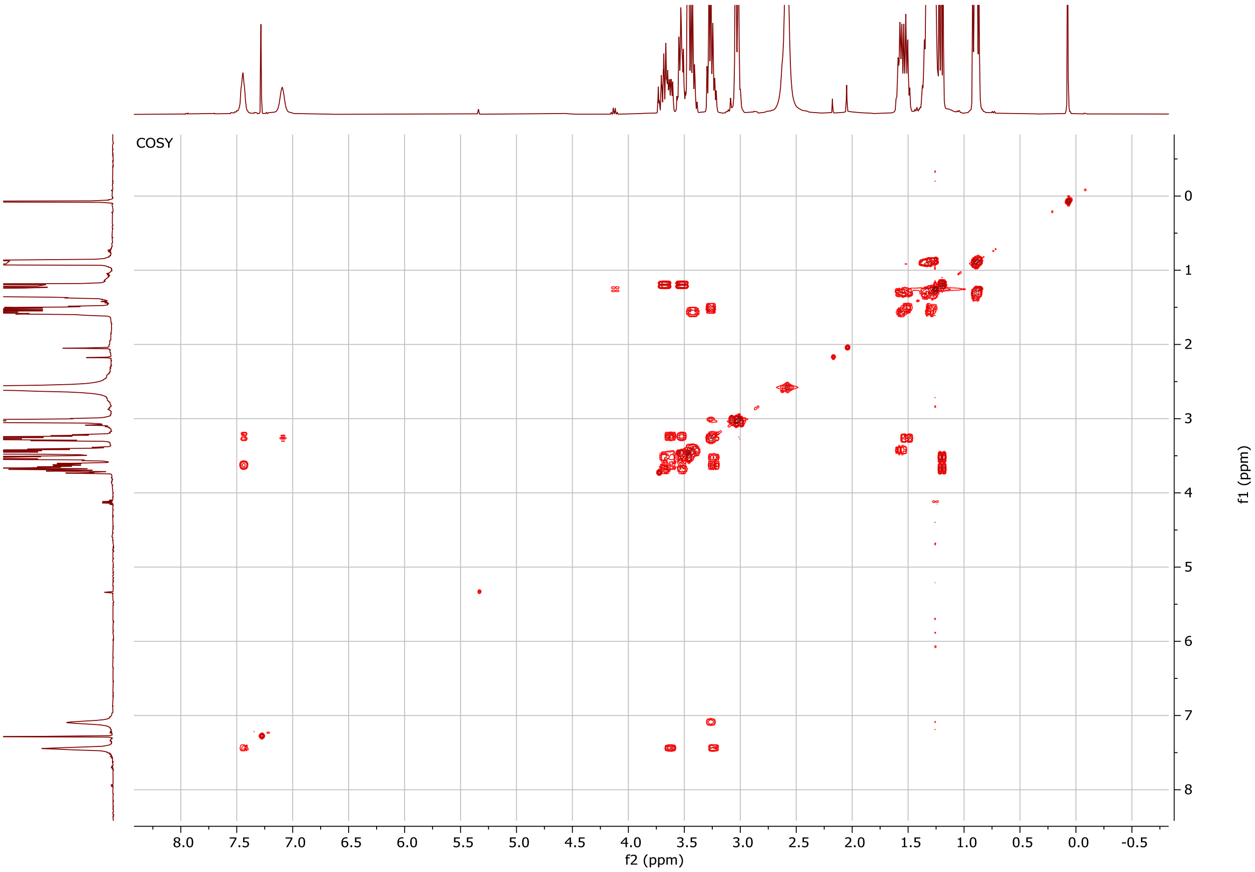

Figure S52. {^{1}H-^{1}H} COSY NMR spectrum of compound **15b.**

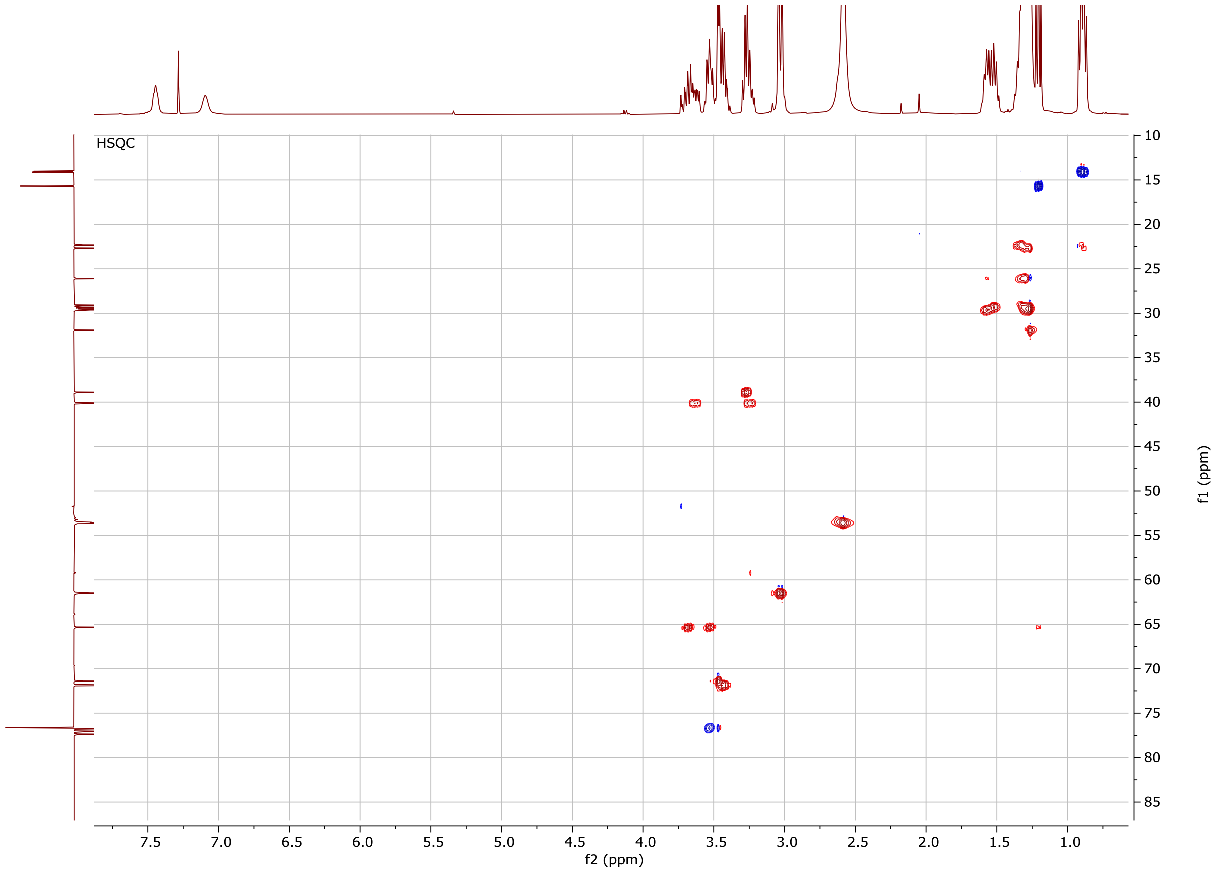

Figure S53. {^{1}H-^{13}C} HSQC NMR spectrum of compound **15b.**

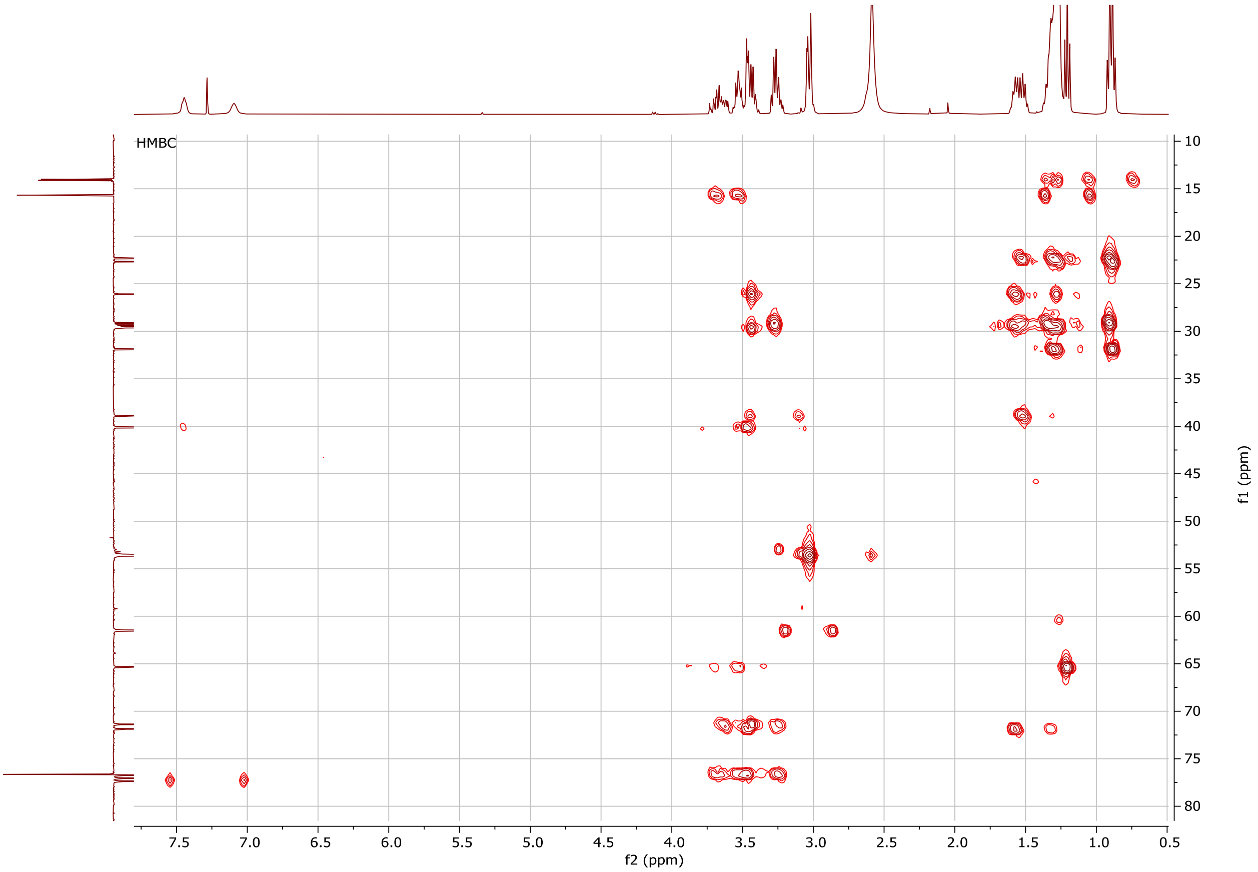

Figure S54. {^{1}H-^{13}C} HMBC NMR spectrum of compound **15b.**

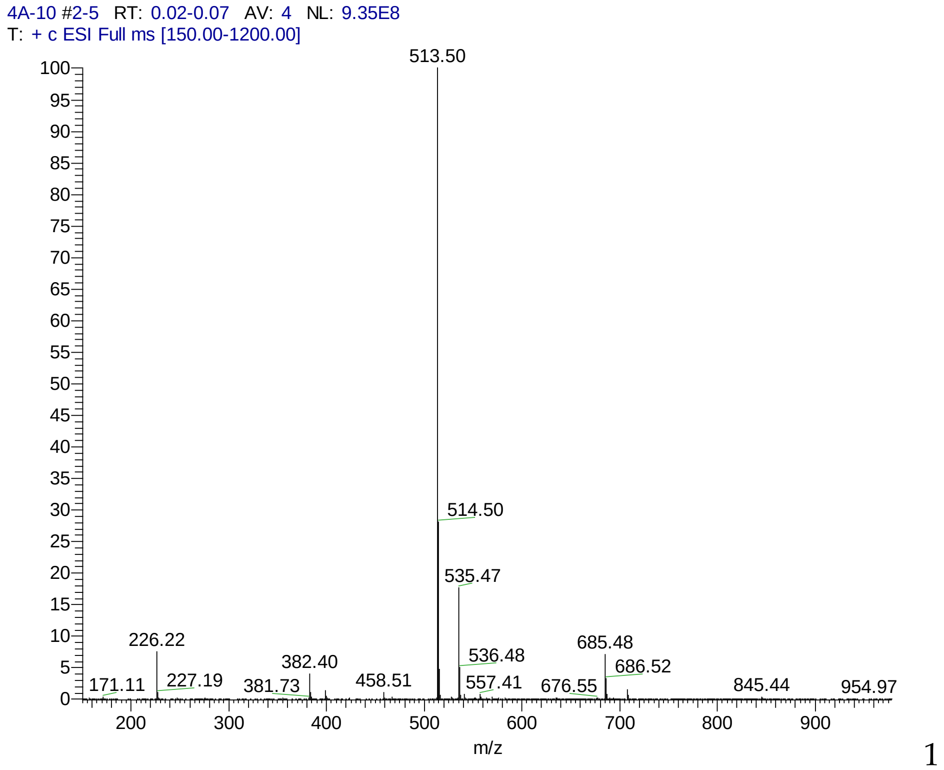

Figure S55. HRMS spectrum of compound **15b.**

N[1]-[*N*-(2,3,4,6-tetra-*O*-acetyl-β-D-glucopyranosyl)aminocarbonyl]methyl-*N*[4]-[*N*-(*rac*-1-decyloxy-2-ethyloxyprop-3-yl)aminocarbonyl]methylpiperazin (**15c**)

Yield: 30%, colorless oil. Eluent: EA-MeOH (95:5) ^{1}H NMR (600 MHz, $CDCl_3$) δ 0.85 (t, *J* = 7.0 Hz, 3H, $(CH_2)_7C\underline{H}_3$), 1.16 (t, 7.0 Hz, 3H, $OCH_2C\underline{H}_3$), 1.21 – 1.31 (m, 14H, $(C\underline{H}_2)_7CH_3$), 1.49 – 1.57 (m, 2H, $OCH_2C\underline{H}_2$), 1.96, 1.98, 2.0, 2.05 (s, 3H, 4 $COCH_3$), 2.46 (m, 4H, CH_2NCH_2 Pip), 2.58 (br.s, 4H, CH_2NCH_2 Pip), 2.92 (dd, *J* = 2.3, 16.7 Hz, 1H, $CHNHC(O)C\underline{H}_aH_b$), 3.02 (d, *J* = 16.4 Hz, 1H, $CH_2NHC(O)C\underline{H}_aH_b$), 3.05 (d, *J* = 16.4 Hz, 1H, $CH_2NHC(O)CH_a\underline{H}_b$),3.09 (dd, *J* = 3.5, 16.7 Hz, 1H, $CHNHC(O)CH_a\underline{H}_b$), 3.21 (dddd, *J* = 4.8, 6.8, 8.0, 13.8 Hz, 1H, $C\underline{H}_aH_bNH$), 3.34 – 3.54 (m, 6H, $C\underline{H}_2OC\underline{H}_aH_b$, $C\underline{H}OC\underline{H}_2CH_3$), 3.56 – 3.69 (m, 2H, $CH_a\underline{H}_bNH$, $CHCH_a\underline{H}_b$), 3.80 (ddd, *J* = 2.2, 4.4, 10.1 Hz, 1H, H-5), 4.05 (dd, *J* = 2.2, 12.5 Hz, 1H, H-6), 4.29 (dd, 1H, *J* = 4.4, 12.5 Hz, H-6), 4.98 (dd, *J* = 9.5, 9.6 Hz, H-2), 5.05 (dd, *J* = 9.4, 10.1 Hz, 1H, H-4), 5.22 (dd, *J* = 9.5, 9.8 Hz, 1H, H-1), 5.28 (dd, *J* = 9.4, 9.6 Hz, 1H, H-3), 7.81 (d, *J* = 9.8 Hz, 1H, $CH_2N\underline{H}$). ^{13}C NMR (151 MHz, $CDCl_3$) δ 14.16, 15.74, 20.55, 20.56, 20.62, 20.78, 22.72, 26.15, 29.36, 29.53, 29.62, 29.66, 29.69, 31.94, 40.20, 40.23, 53.24, 53.70, 61.38, 68.25, 70.46, 71.46, 71.47, 72.95, 73.79, 76.70, 76.74, 76.93, 77.15, 77.36, 77.82, 169.55, 169.94, 170.18, 170.21, 170.61, 171.13. HRMS ESI $[M+H]^+$ calcd for $C_{37}H_{65}N_4O_{13}$ 773.4543, found 773.4535.

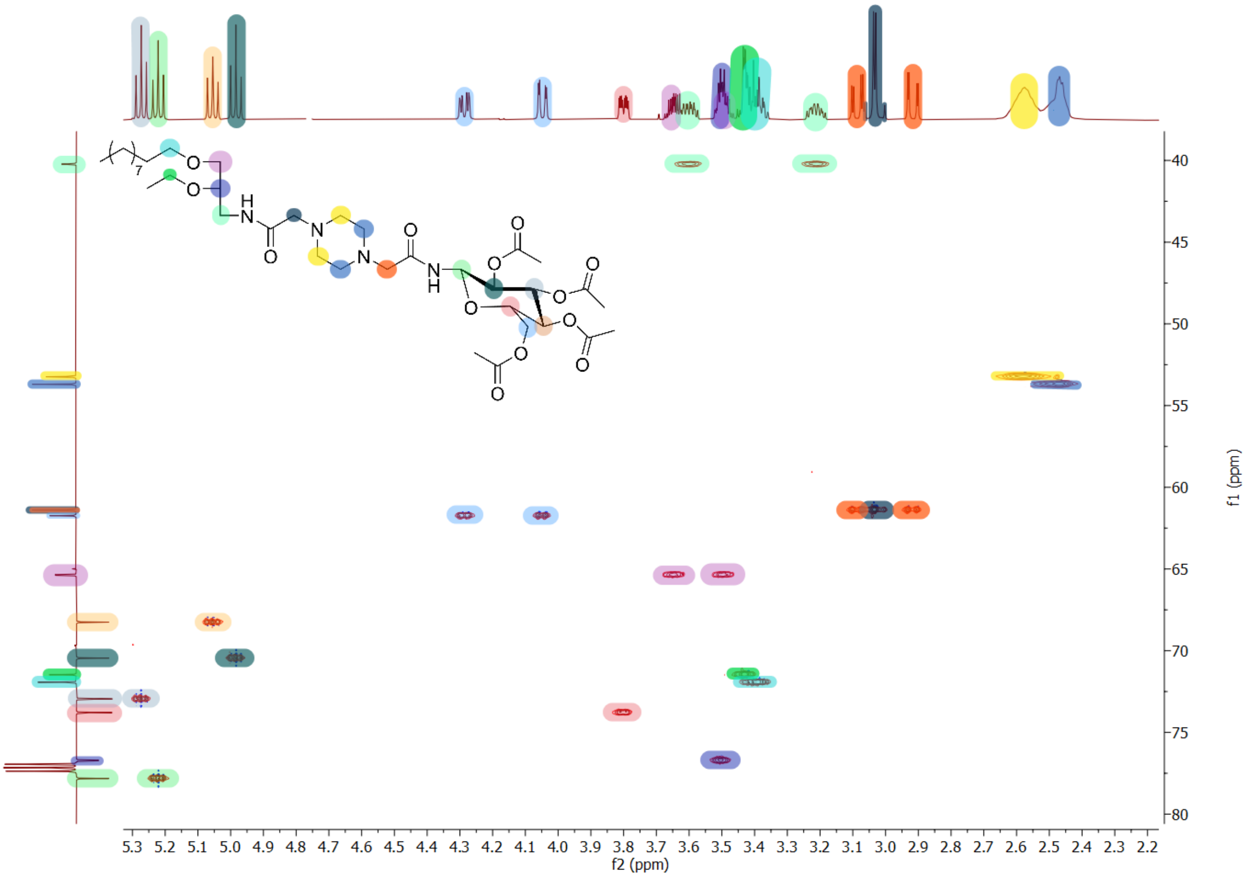

Figure S56. Fragment of {^{1}H-^{13}C} HSQC NMR spectrum of compound **15c.**

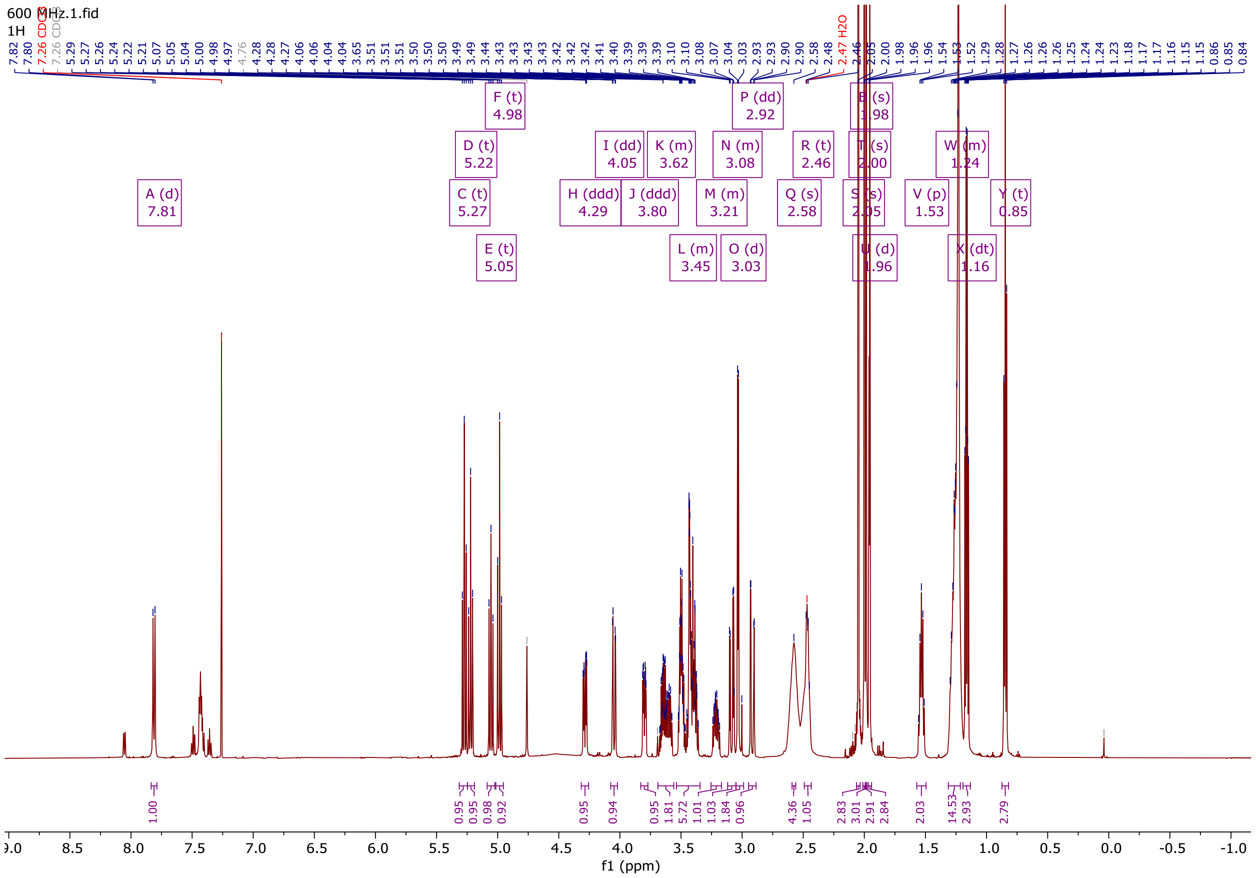

Figure S57. ^{1}H NMR spectrum of compound **15c.**

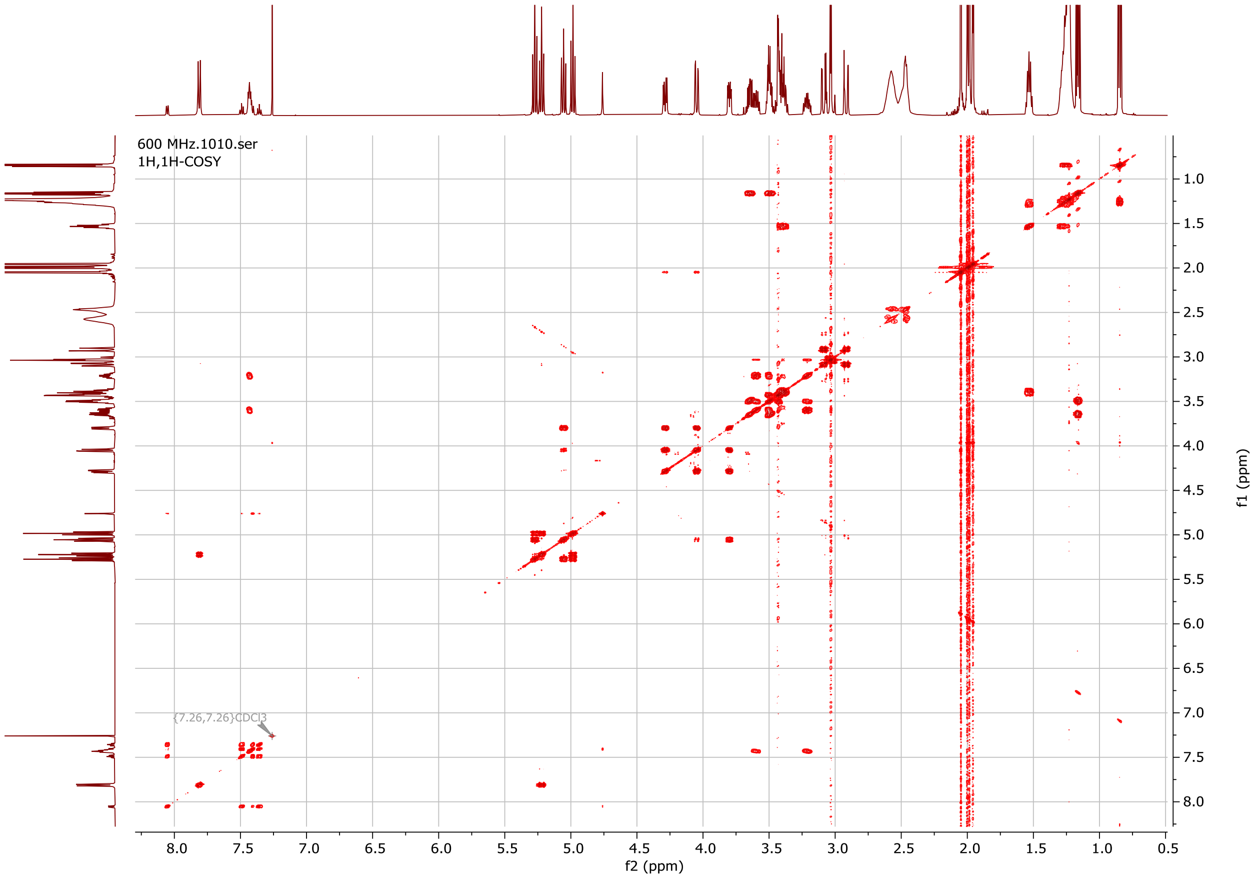

Figure S58. {^{1}H-^{1}H} COSY NMR spectrum of compound **15c.**

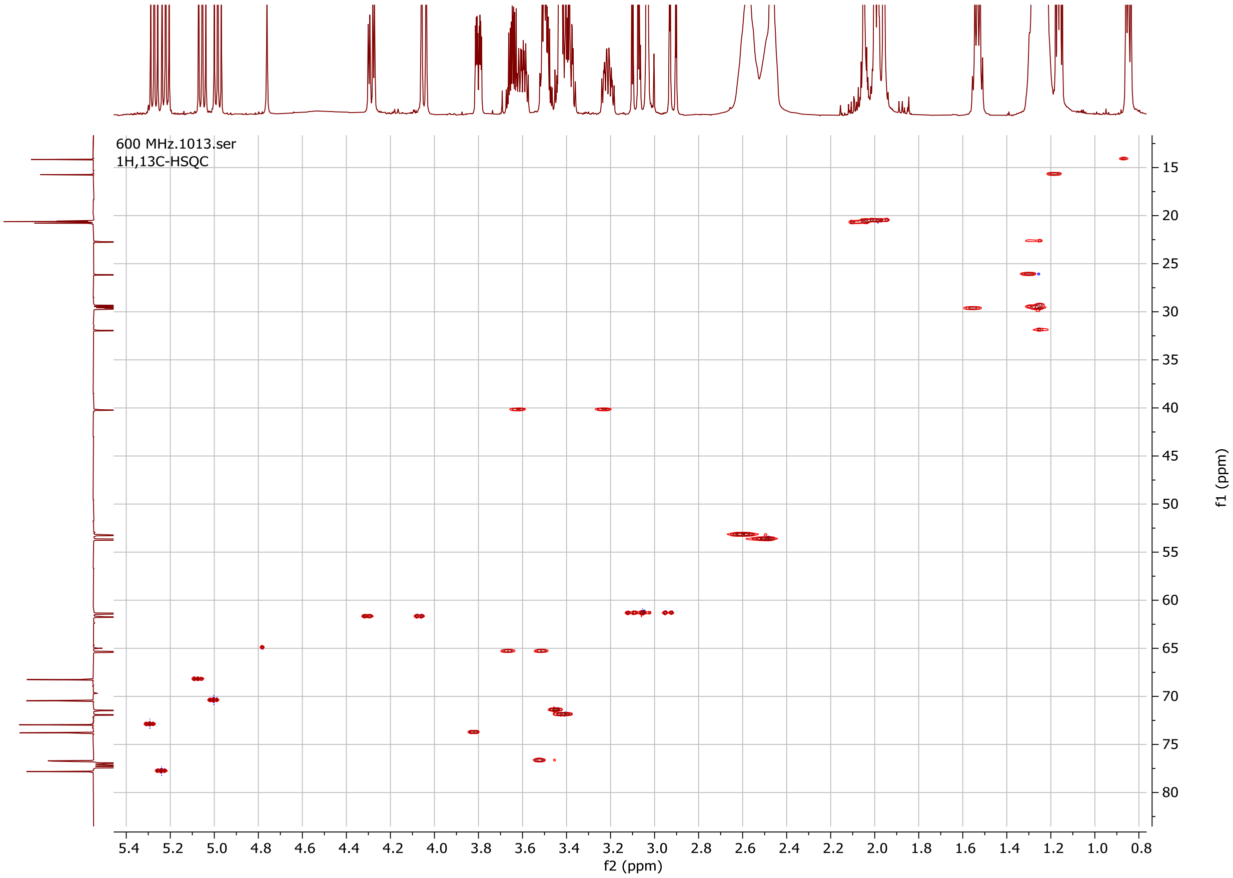

Figure S59. {^{1}H-^{13}C} HSQC NMR spectrum of compound **15c.**

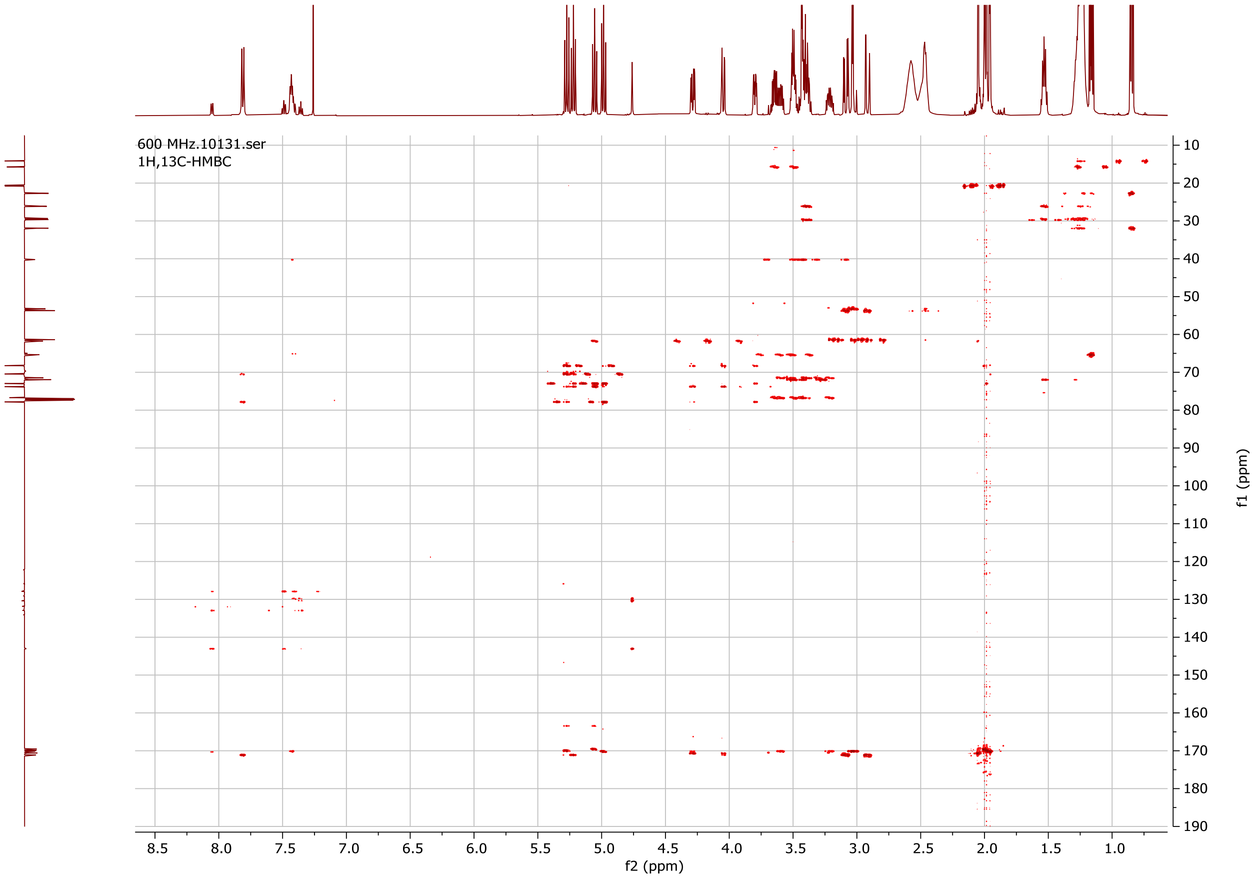

Figure S60. {^{1}H-^{13}C} HMBC NMR spectrum of compound **15c.**

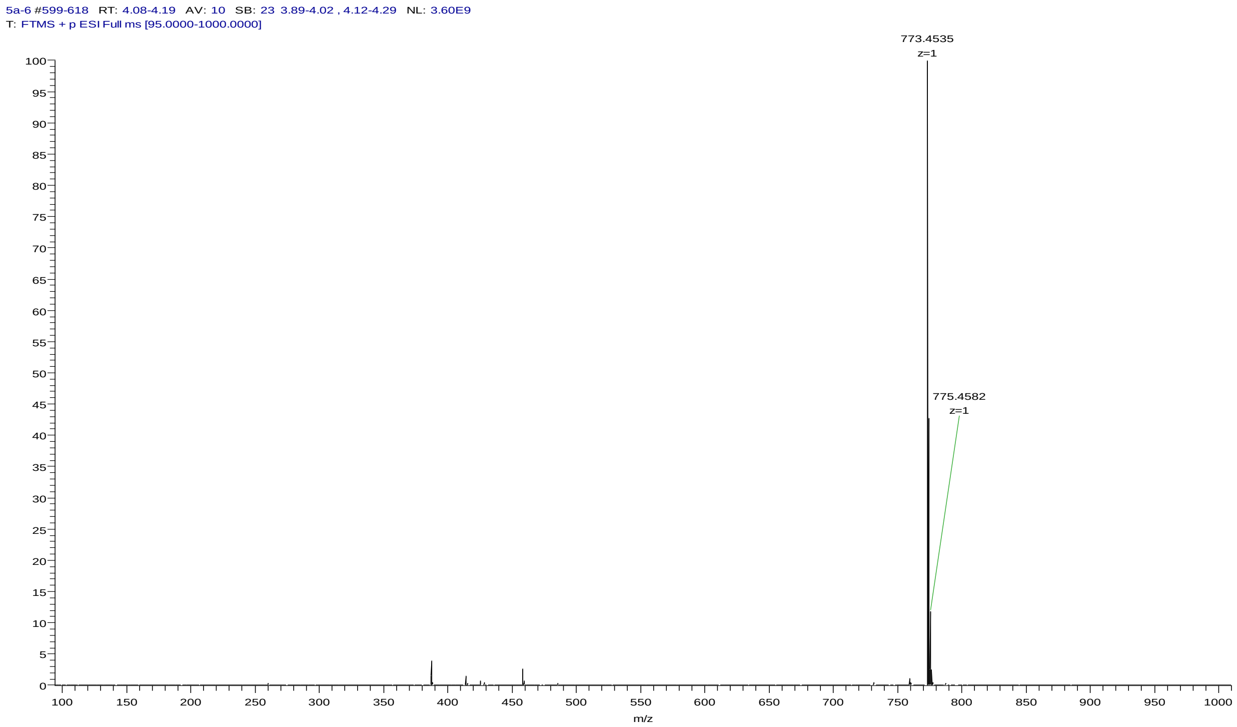

Figure S61. HRMS spectrum of compound **15c.**

1,9-diamino-N^9-ethyl-N^1-octadecyl-N^3,N^7-dibenzyl-3,7-diazanonane (**16a**)

Yield 410 mg (66%), colorless oil. Eluent: ACN-NH_3·H_2O (9:1). ^{1}H NMR (400 MHz, $CDCl_3$) δ 0.91 (t, *J* = 7.0 Hz, 3H, $(CH_2)_{15}C\underline{H}_3$), 1.07 (t, *J* = 7.1 Hz, 3H, $CH_2C\underline{H}_3$), 1.29 (s, 30H, $(C\underline{H}_2)_{15}CH_3$), 1.44 (m, 2H, $CH_2C\underline{H}_2(CH_2)_{15}$), 1.61 – 1.79 (m, 2H, $PhCH_2NCH_2C\underline{H}_2CH_2$), 2.34 – 2.75 (m, 16H, 4 $NHC\underline{H}_2$, 4 NCH_2), 3.55 (s, 4H, 2 $PhCH_2$), 7.16 – 7.43 (m, 10H, 2 Ph). ^{13}C NMR (101 MHz, $CDCl_3$) δ 14.17, 15.14, 22.73, 24.77, 29.40, 29.65, 29.70, 29.75, 43.94, 47.19, 47.44, 49.94, 52.63, 52.65, 53.66, 59.01, 59.03, 126.85, 126.94, 128.22, 128.39, 128.78, 128.84, 134.17, 139.81, 139.84. MS ESI m/z: $[M+H]^+$ calcd for $C_{41}H_{73}N_4$ 621.58, found: 621.52.

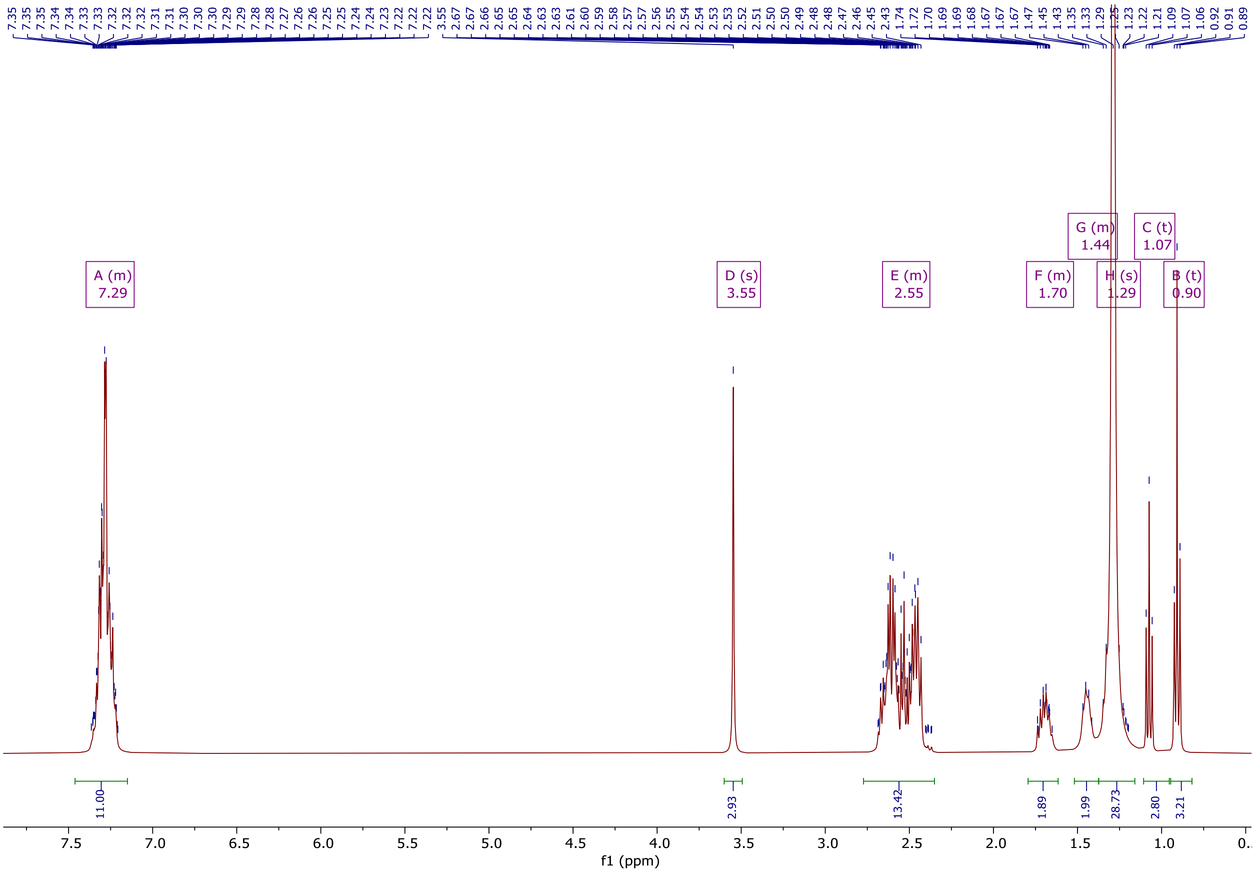

Figure S62. ^{1}H NMR spectrum of compound **16a.**

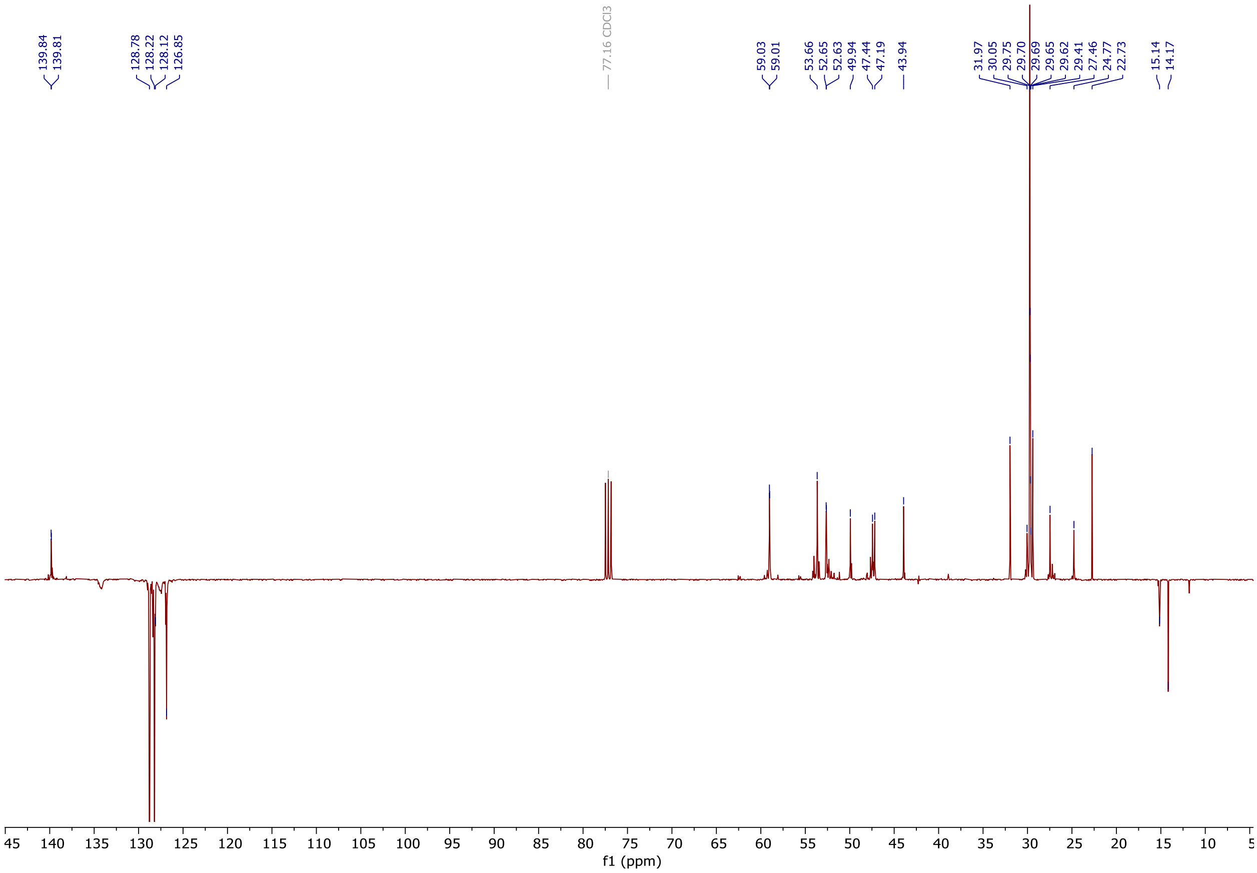

Figure S63. ^{1}H (APT) NMR spectrum of compound **16a.**

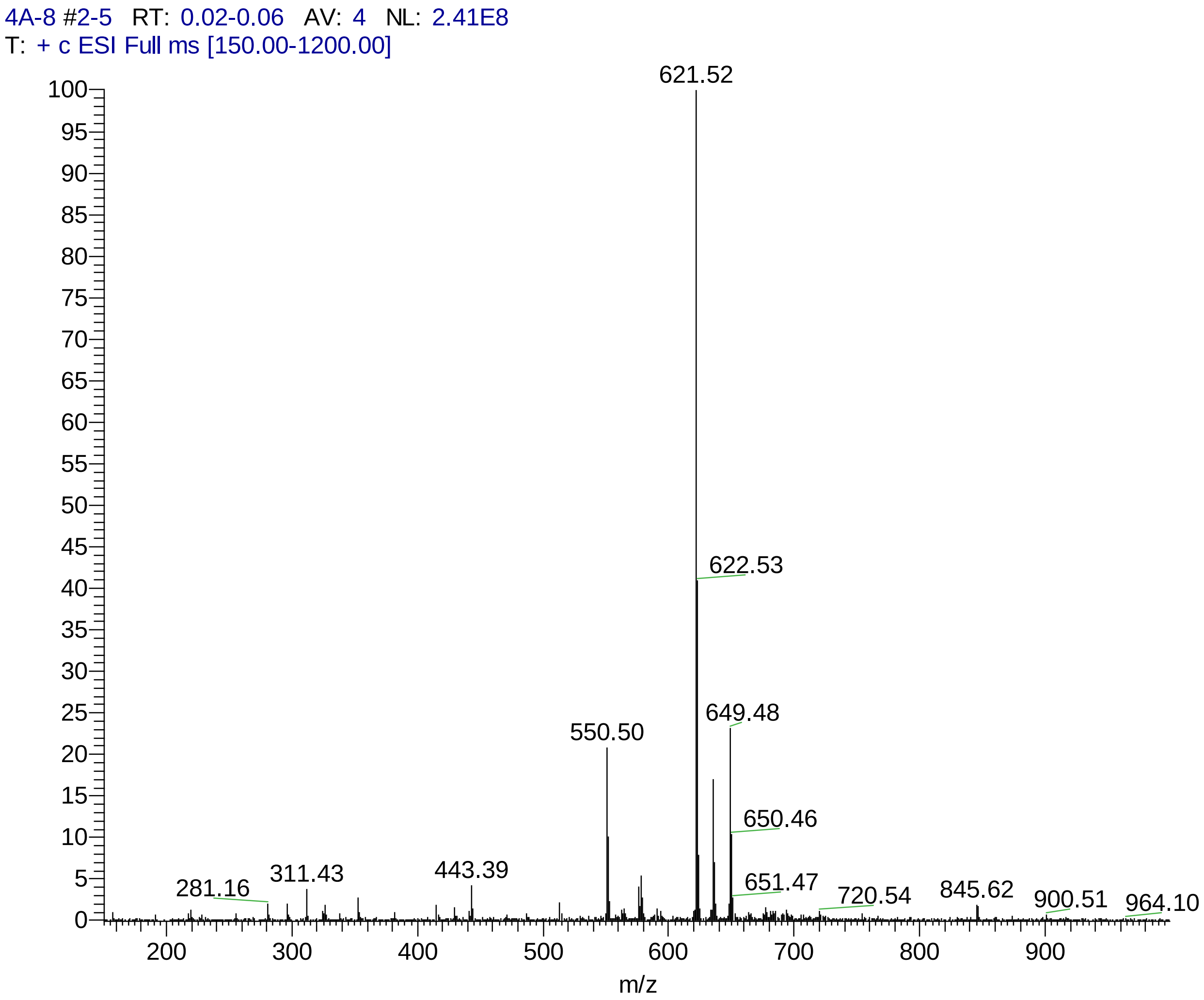

Figure S64. HRMS spectrum of compound **16a.**

N^1-ethyl-N^4-[(N-octadecyl)aminoethyl]piperazin (**16b**)

Yield: 31%, colorless oil. Eluent: ACN-$NH_3 \cdot H_2O$ (95:5). ^{1}H NMR (300 MHz, MeOD) δ 0.90 (t, J = 6.5 Hz, 3H, $(CH_2)_{15}C\underline{H}_3$), 1.10 (td, J = 3.2, 7.2 Hz, 3H, NCH_2CH_3), 1.31 (br.s, 30H, $(C\underline{H}_2)_{15}CH_3$), 1.55 – 1.75 (m, 2H, $CH_2C\underline{H}_2(CH_2)_{15}$), 2.14 (td, J = 2.8, 12.5 Hz, 1H, $NHC\underline{H}_aH_bCH_2N$), 2.39 – 3.11 (m, 13H, Pip protons, $NHCH_a\underline{H}_bC\underline{H}_2N$, $NC\underline{H}_2CH_3$). ^{13}C NMR (75 MHz, MeOD) δ 11.80, 14.49, 23.77, 27.11, 28.01, 30.47, 30.51, 30.71, 30.82, 33.10, 52.34, 52.48, 52.70, 53.17, 53.28, 53.69, 54.65, 56.54. HRMS ESI m/z: $[M+H]^+$ calcd for $C_{26}H_{56}N_3$ 410.4469, found: 410.4468.

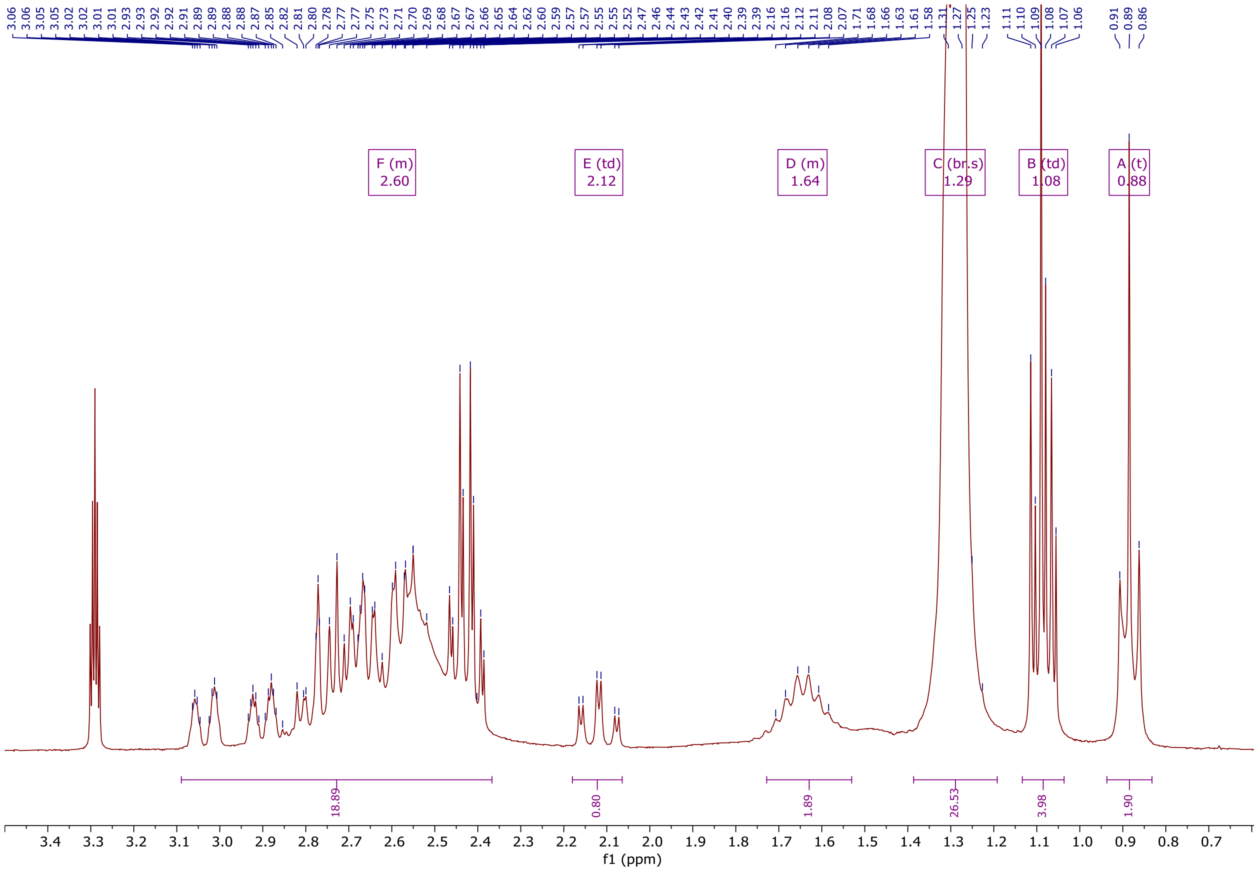

Figure S65. ^{1}H NMR spectrum of compound **16b.**

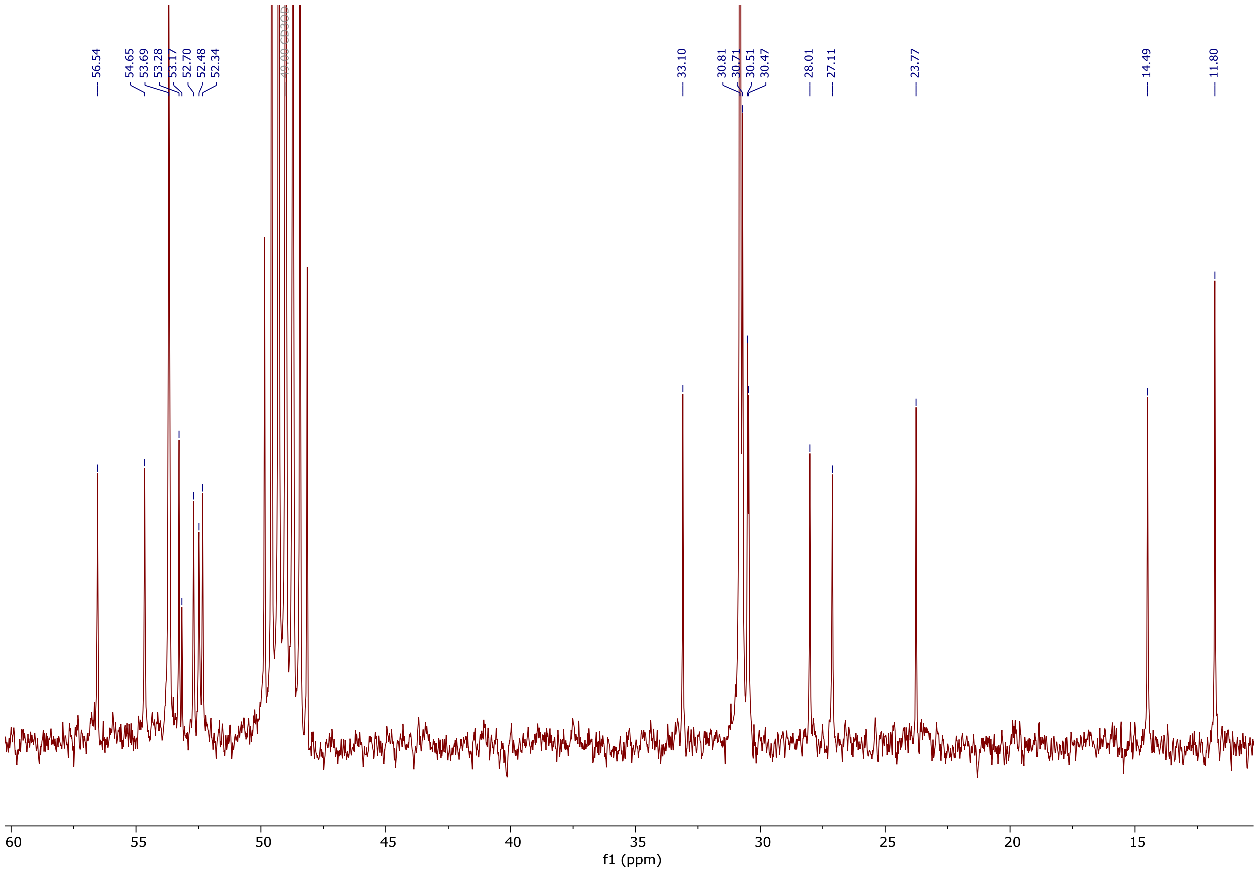

Figure S66. ^{13}C NMR spectrum of compound **16b.**

Display Report

Analysis Info

Analysis Name	D:\Data\Kolotyrkina\2021\Maksimenko\0415003.d	Acquisition Date	15.04.2021 9:28:17
Method	tune_50-1600.m	Operator	BDAL@DE
Sample Name	/MAKS 5A-42	Instrument / Ser#	micrOTOF 10248
Comment	C26H55N3 mH 410.4468 calibrant added CH3CN		

Acquisition Parameter

Source Type	ESI	Ion Polarity	Positive	Set Nebulizer	1.0 Bar
Focus	Not active			Set Dry Heater	200 °C
Scan Begin	50 m/z	Set Capillary	4500 V	Set Dry Gas	4.0 l/min
Scan End	1600 m/z	Set End Plate Offset	-500 V	Set Divert Valve	Waste

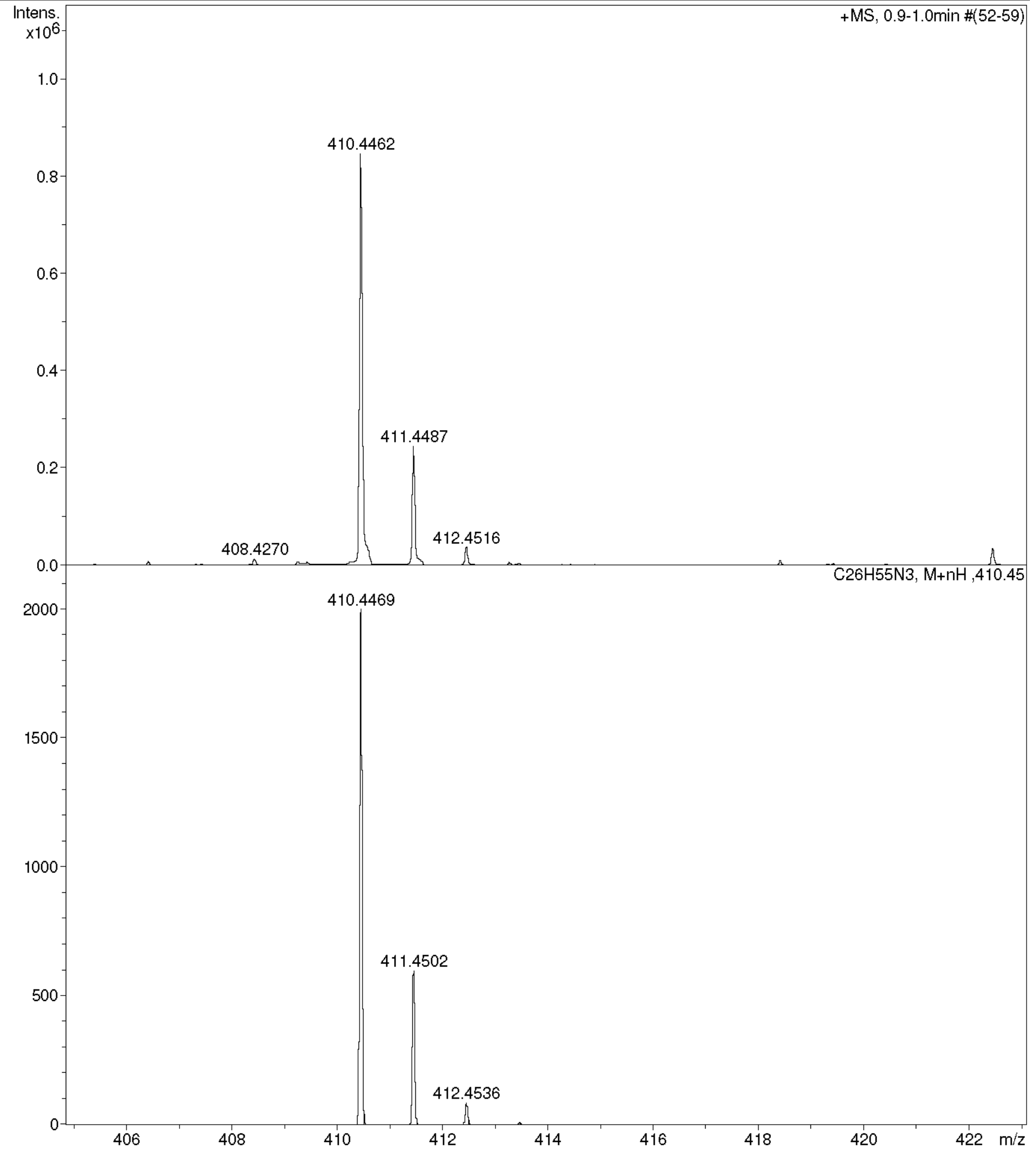

Bruker Compass DataAnalysis 4.0 printed: 15.04.2021 9:31:23 Page 1 of 1

Figure S67. HRMS spectrum of compound **16b.**

N[1]-[2-(ethylamino)ethyl]-*N*[4]-[*N*-(*rac*-1-decyloxy-2-ethyloxyprop-3-yl)amino]ethylpiperazin (**16c**)

Yield: 23%, colorless oil. Eluent: ACN-$NH_3{\cdot}H_2O$ (9:1). ^{1}H NMR (600 MHz, CD_2Cl_2) δ 0.88 (t, *J* = 7.0 Hz, 3H, $(CH_2)_7C\underline{H}_3$), 1.03 (t, *J* = 7.2 Hz, 3H, $NCH_2C\underline{H}_3$), 1.16 (t, *J* = 7.0 Hz, $OCH_2C\underline{H}_3$, 3H), 1.29 (br.s, $(C\underline{H}_2)_7CH_3$, 14H), 1.51 – 1.57 (m, 3H, $CH_2C\underline{H}_2(CH_2)_7$), 2.35 (q, *J* = 7.2 Hz, 2H, $NHC\underline{H}_2CH_3$), 2.37-2.58 (m, 14H, $3C\underline{H}_2NH$, Pip protons), 2.58 – 2.71 (m, 4H, $C\underline{H}_2N(CH_2CH_2)_2NC\underline{H}_2$), 3.38 – 3.46 (m, 4H, $C\underline{H}_2OC\underline{H}_2$), 3.48 – 3.54 (m, 2H, $C\underline{H}OC\underline{H}_aH_bCH_3$), 3.65 (dq, *J* = 7.0, 9.3 Hz, 1H, $OCH_a\underline{H}_bCH_3$). ^{13}C NMR (151 MHz, CD_2Cl_2) δ 12.35, 14.30, 16.00, 23.12, 26.60, 29.77, 29.93, 30.03, 30.08, 30.18, 32.35, 47.14, 51.64, 52.64, 53.40, 53.81, 58.33, 65.62, 71.95, 72.28, 78.26.

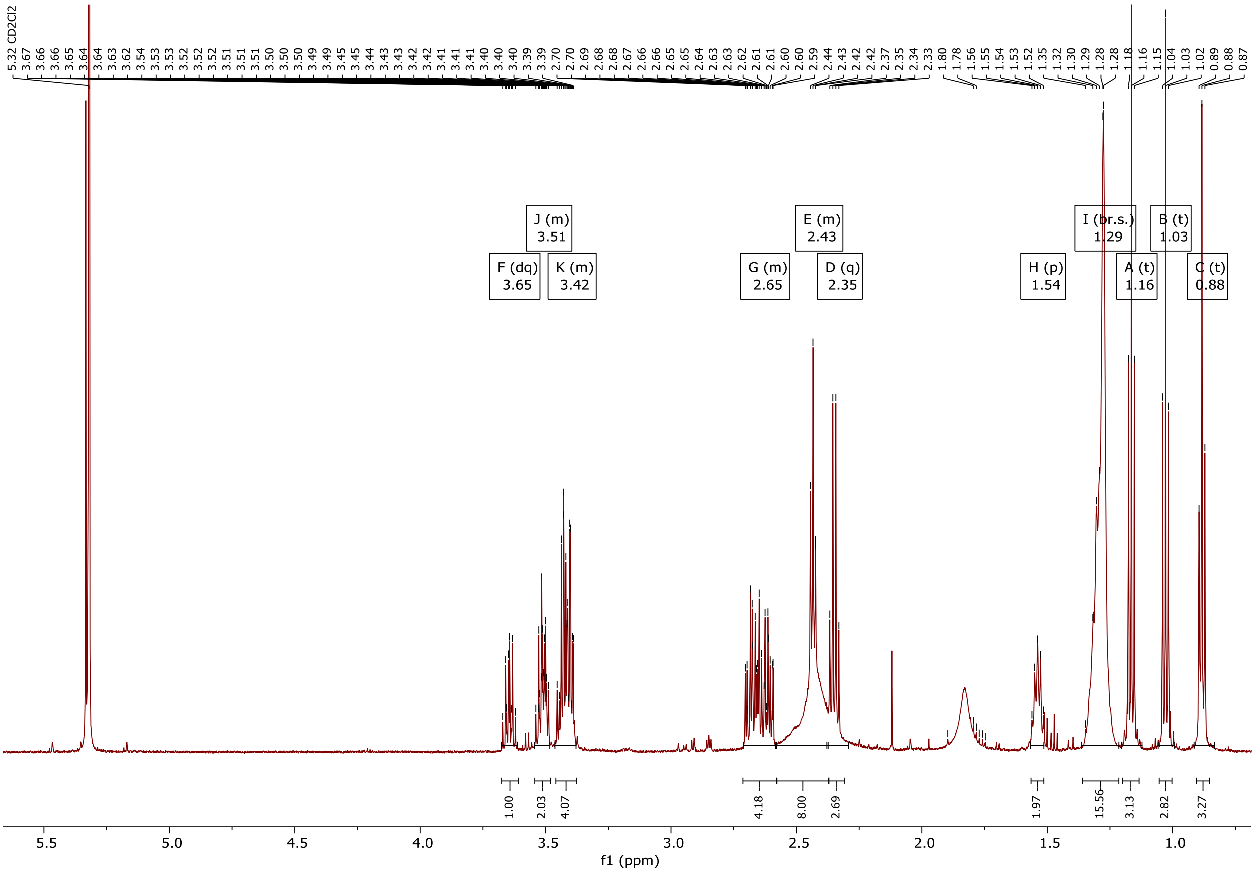

Figure S68. ^{1}H NMR spectrum of compound **16c.**

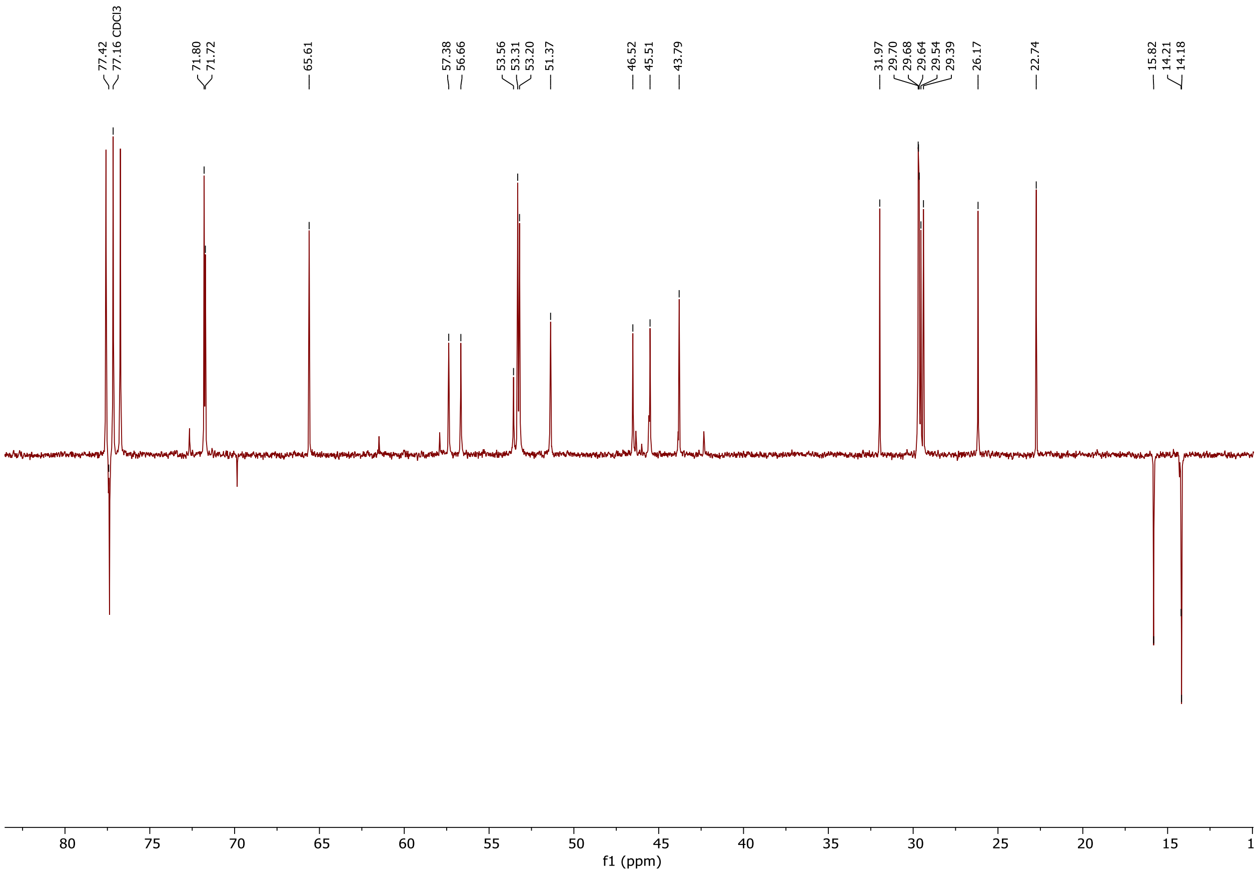

Figure S69. ^{13}C (APT) NMR spectrum of compound **16c.**

N^1-ethyl-N^4-[N-(*rac*-1-decyloxy-2-ethyloxyprop-3-yl)amino]ethylpiperazin (**16d**)

Yield 184 mg (38%), colorless oil. Eluent: EA-MeOH-$NH_3{\cdot}H_2O$ (7:3:0.2). ^{1}H NMR (300 MHz, $CDCl_3$) δ 0.77 – 0.94 (t, 3H, $(CH_2)_7C\underline{H}_3$), 1.09 – 1.34 (m, 20H, $(C\underline{H}_2)_7CH_3$, $OCH_2C\underline{H}_3$, $NCH_2C\underline{H}_3$), 1.45 – 1.59 (m, 2H, $CH_2C\underline{H}_2(CH_2)_{15}$), 2.31 – 2.82 (m, 16H, 2 $NHC\underline{H}_2$, Pip protons), 3.30 – 3.75 (m, 7H, $C\underline{H}_2OC\underline{H}_2$, $C\underline{H}OC\underline{H}_2$). ^{13}C NMR (75 MHz, $CDCl_3$) δ 14.18, 14.21, 15.82, 22.74, 26.17, 29.39, 29.54, 29.64, 29.68, 29.70, 31.97, 43.79, 45.51, 46.52, 51.37, 53.20, 53.31, 53.56, 56.66, 57.38, 65.61, 71.72, 71.80, 77.42. HRMS ESI m/z: $[M+2Na]^{2+}$ calcd for $C_{23}H_{49}N_3O_2Na_2$ 222.6805, found: 222.2215.

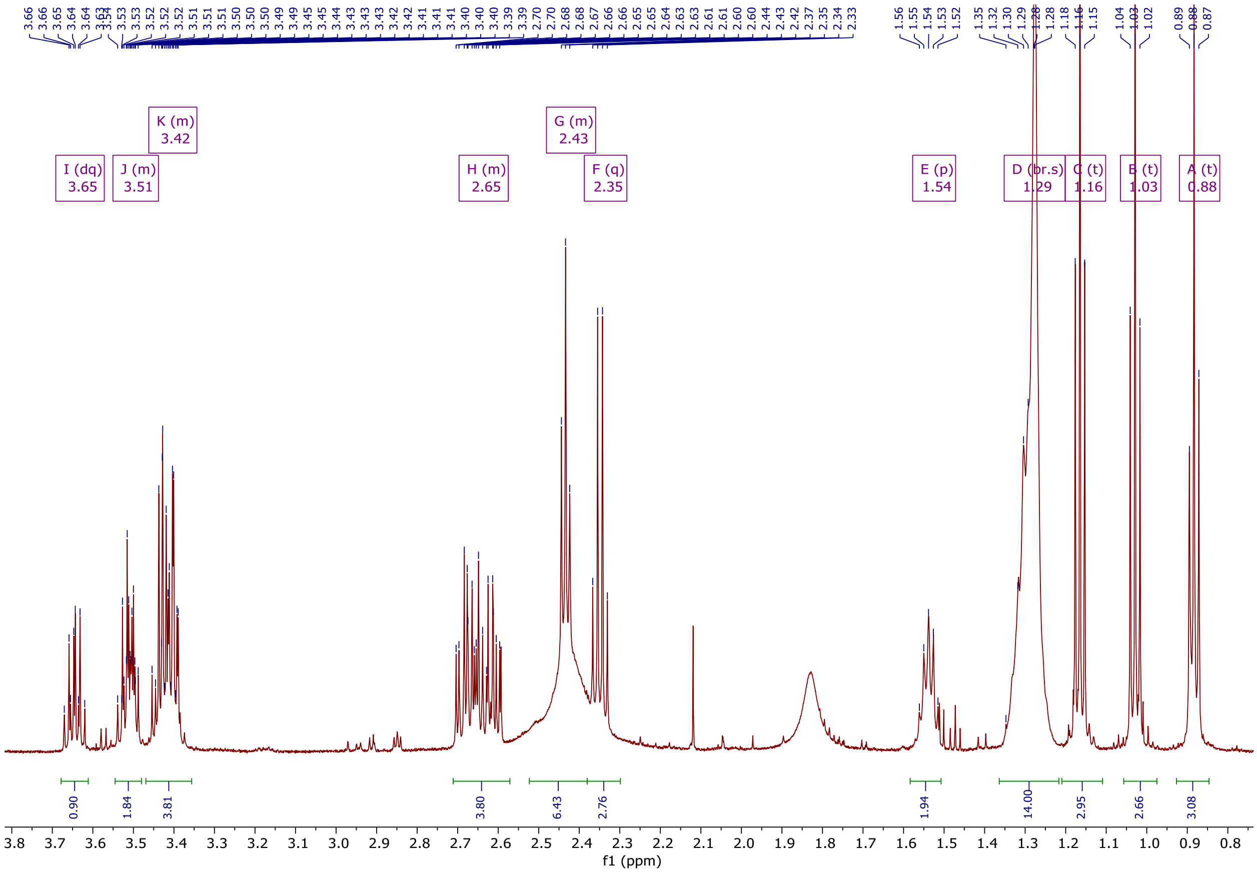

Figure S70. ^{1}H NMR spectrum of compound **16d.**

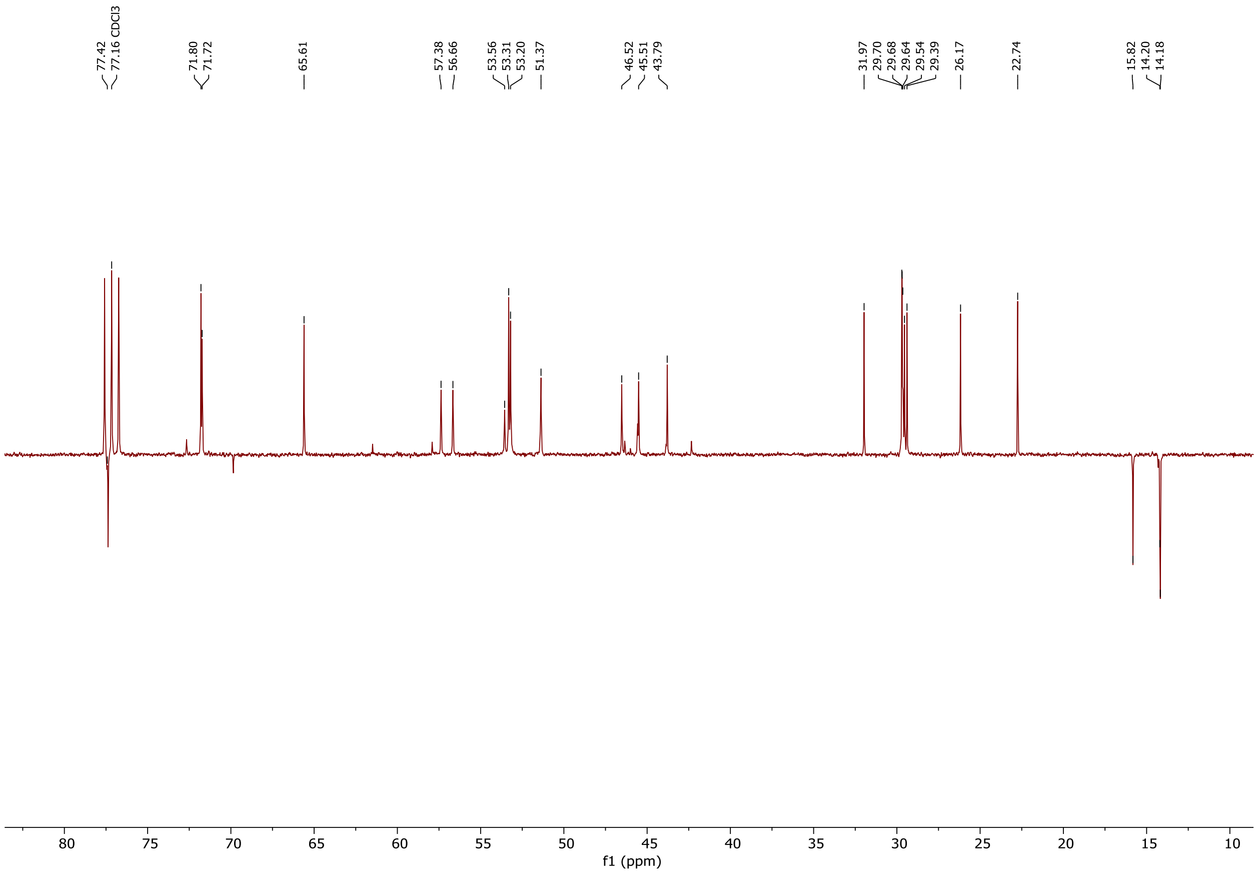

Figure S71. ^{13}C (APT) NMR spectrum of compound **16d.**

Display Report

Analysis Info

Analysis Name	D:\Data\Kolotyrkina\2021\Maksimenko\0413019.d	Acquisition Date	13.04.2021 12:29:16
Method	tune_50-1600.m	Operator	BDAL@DE
Sample Name	/MAKS 5A-56	Instrument / Ser#	micrOTOF 10248
Comment	C23H49N3O2 mH 400.3897 calibrant added CH3OH		

Acquisition Parameter

Source Type	ESI	Ion Polarity	Positive	Set Nebulizer	1.0 Bar
Focus	Not active			Set Dry Heater	200 °C
Scan Begin	50 m/z	Set Capillary	4500 V	Set Dry Gas	4.0 l/min
Scan End	1600 m/z	Set End Plate Offset	-500 V	Set Divert Valve	Waste

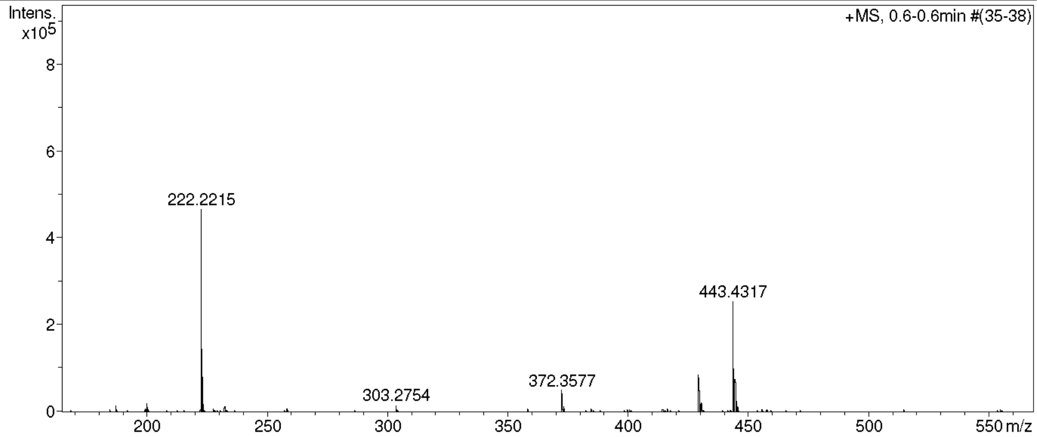

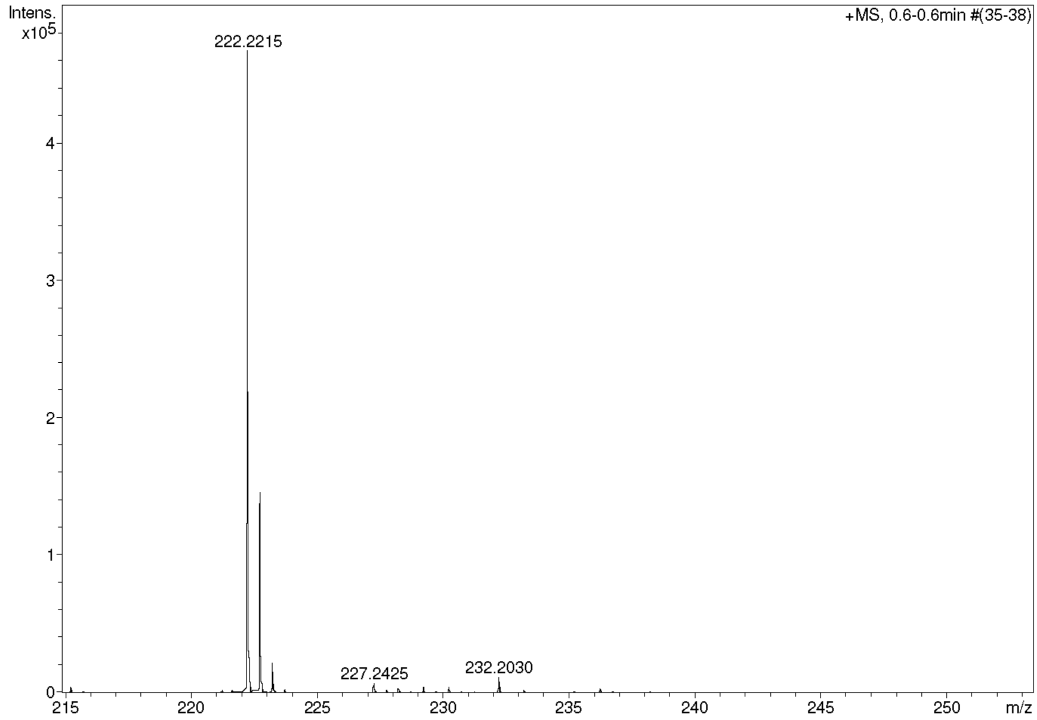

Figure S72. HRMS spectrum of compound **16d.**

N[1]-[2-[*N*-(isopropylamino)ethyl]-*N*[4]-[*N*-(*rac*-1-decyloxy-2-ethyloxyprop-3-yl)amino]ethylpiperazin (**16e**)

Yield: 25%, colorless oil. Eluent: EA-MeOH-$NH_3 \cdot H_2O$ (7:3:0.3). ^1H NMR (300 MHz, $CDCl_3$) δ 0.86 (t, *J* = 7.0 Hz, 3H, $(CH_2)_7C\underline{H}_3$), 1.08 (d, *J* = 6.3 Hz, 6H, $CH(C\underline{H}_3)_2$), 1.18 (t, *J* = 7.0 Hz, 3H, $OCH_2C\underline{H}_3$), 1.25 (d, *J* = 5.6 Hz, 14H, $(C\underline{H}_2)_7CH_3$), 1.47 – 1.60 (m, 2H, $CH_2C\underline{H}_2(CH_2)_7$), 2.34 – 2.58 (m, 12H, 2 CH_2N and Pip protons), 2.60 – 2.79 (m, 6H, 3 $NHC\underline{H}_2$), 2.82 (sept, *J* = 6.3 Hz, 1H, $C\underline{H}(CH_3)_2$), 3.36 – 3.63 (m, 6H, $C\underline{H}_2OC\underline{H}_2$, $C\underline{H}OC\underline{H}_aH_b$), 3.69 (dq, *J* = 7.0, 9.3 Hz, 1H, $CHOCH_a\underline{H}_b$). ^{13}C NMR (75 MHz, $CDCl_3$) δ 14.21, 15.86, 22.69, 22.79, 26.23, 29.44, 29.59, 29.69, 29.73, 29.77, 32.02, 43.83, 46.74, 49.15, 51.57, 53.38, 57.59, 57.74, 65.66, 71.83, 71.92, 77.66. HRMS ESI m/z: $[M+H]^+$ calcd for $C_{26}H_{57}N_4O_2$ 457.4476, found: 457.4483. HRMS ESI m/z: $[M+2H]^{2+}$ calcd for $C_{26}H_{58}N_4O_2$ 229.2275, found: 229.2276.

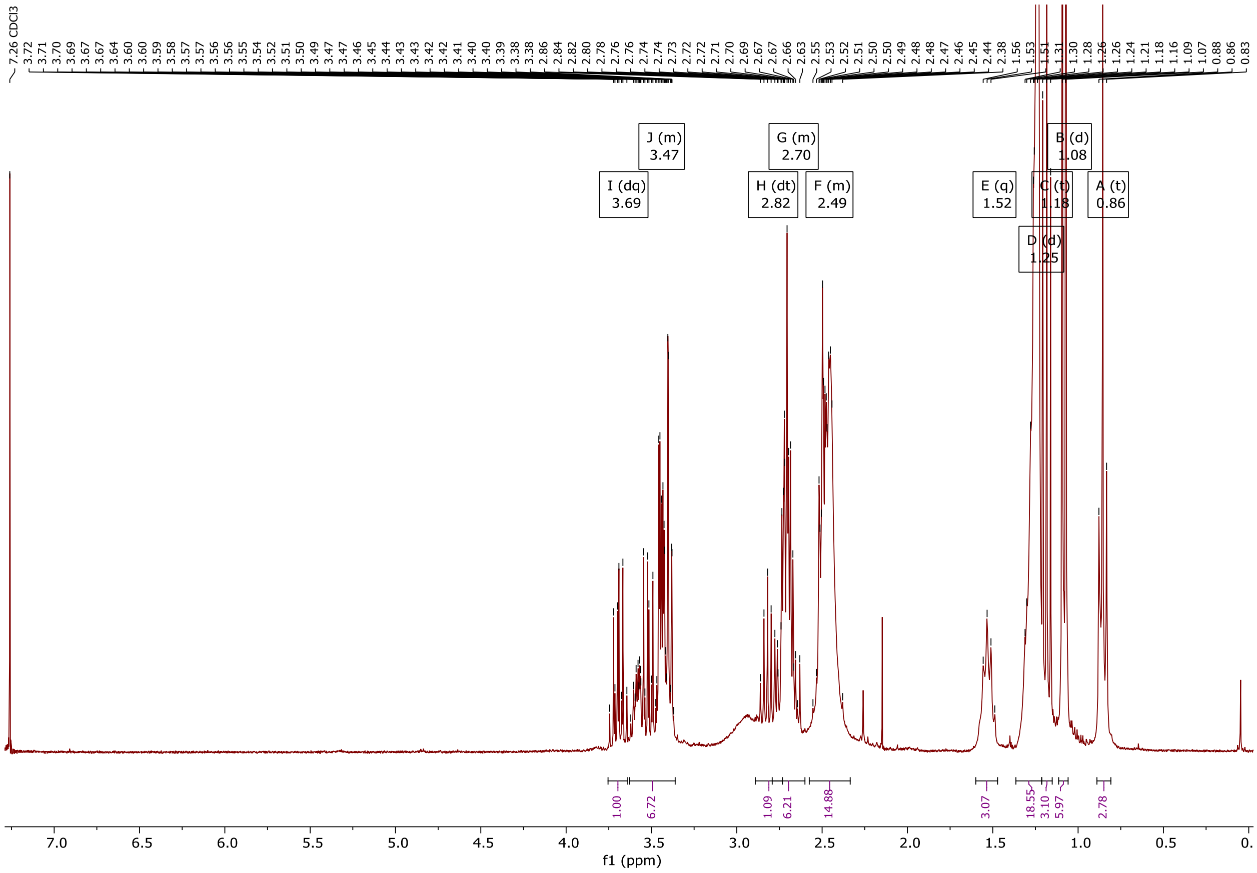

Figure S73. ^1H NMR spectrum of compound **16e.**

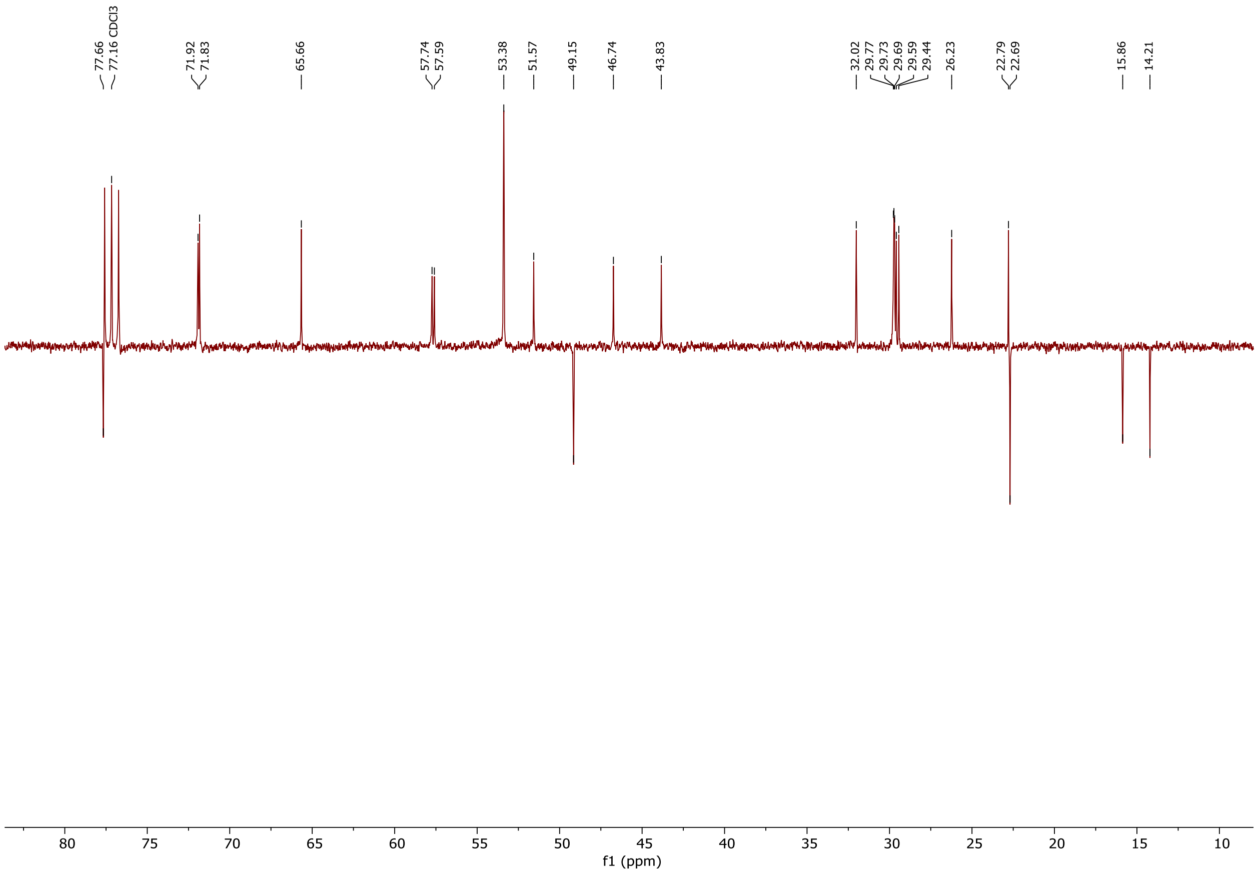

Figure S74. ^{13}C (APT) NMR spectrum of compound **16e.**

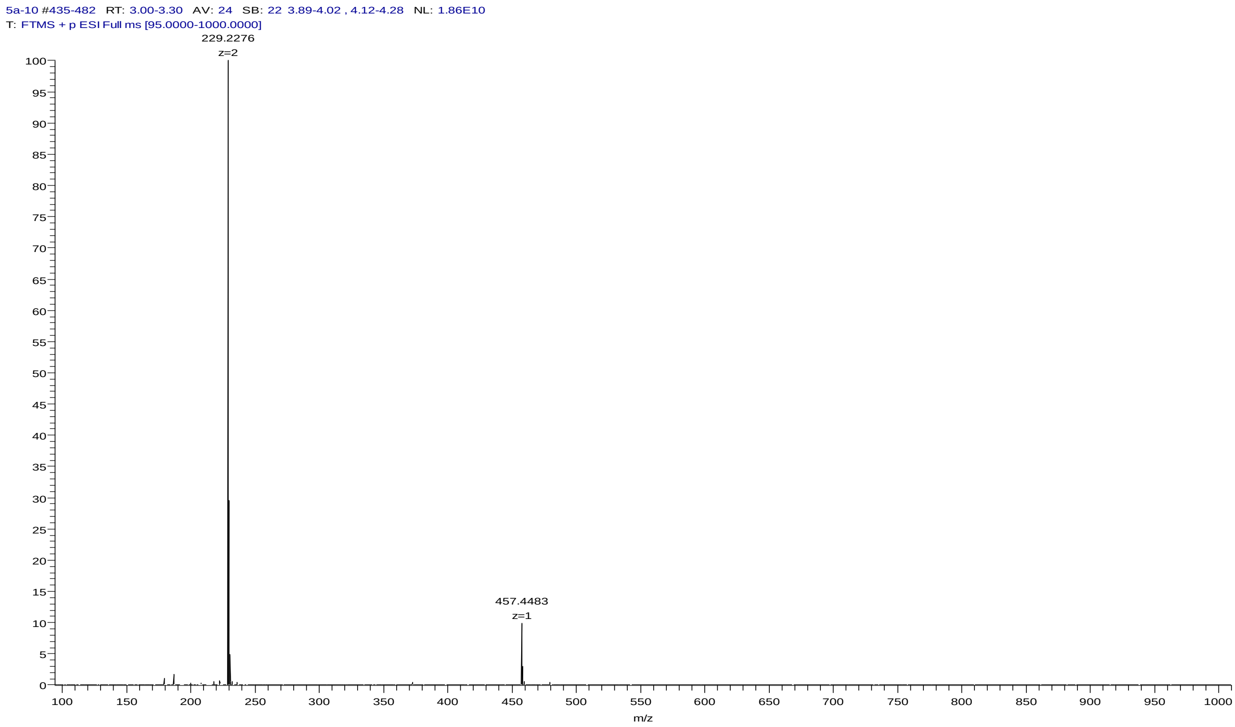

Figure S75. HRMS spectrum of compound **16e.**

N^1-[2-(N-pentylamino)ethyl]-N^4-[N-(*rac*-1-decyloxy-2-ethyloxyprop-3-yl)amino]ethylpiperazin (**16f**)

Yield: 43%, colorless oil. Eluent: ACN-$NH_3 \cdot H_2O$ (9:1). ^1H NMR (300 MHz, $CDCl_3$) δ 0.79 – 0.91 (m, 6H, $(CH_2)_7C\underline{H}_3$, $(CH_2)_2C\underline{H}_3$), 1.17 (t, J = 7.0 Hz, 3H, $OCH_2C\underline{H}_3$), 1.25 (br.s, 18H, $(C\underline{H}_2)_7CH_3$, $(C\underline{H}_2)_2CH_3$), 1.41 – 1.58 (m, 4H, $CH_2C\underline{H}_2(CH_2)_7$, $NCH_2C\underline{H}_2$), 2.13 – 2.76 (m, 20H, 2 NCH_2, 4 $NHC\underline{H}_2$, Pip protons), 3.32 – 3.60 (m, 1H, $C\underline{H}_2OC\underline{H}_2$, $C\underline{H}OC\underline{H}_aH_b$), 3.68 (dq, J = 7.1, 9.4 Hz, 1H, $OCH_a\underline{H}_bCH_3$). ^{13}C NMR (101 MHz, $CDCl_3$) δ 14.16, 14.21, 22.68, 22.77, 26.21, 29.42, 29.57, 29.64, 29.67, 29.71, 29.74, 29.78, 31.99, 46.55, 46.77, 50.14, 51.64, 53.39, 53.43, 57.95, 65.65, 71.77, 71.99, 77.82. HRMS ESI m/z: $[M+2Na]^+$ calcd for $C_{23}H_{49}N_3O_2Na_2$ 222.6805, found: 222.2215.

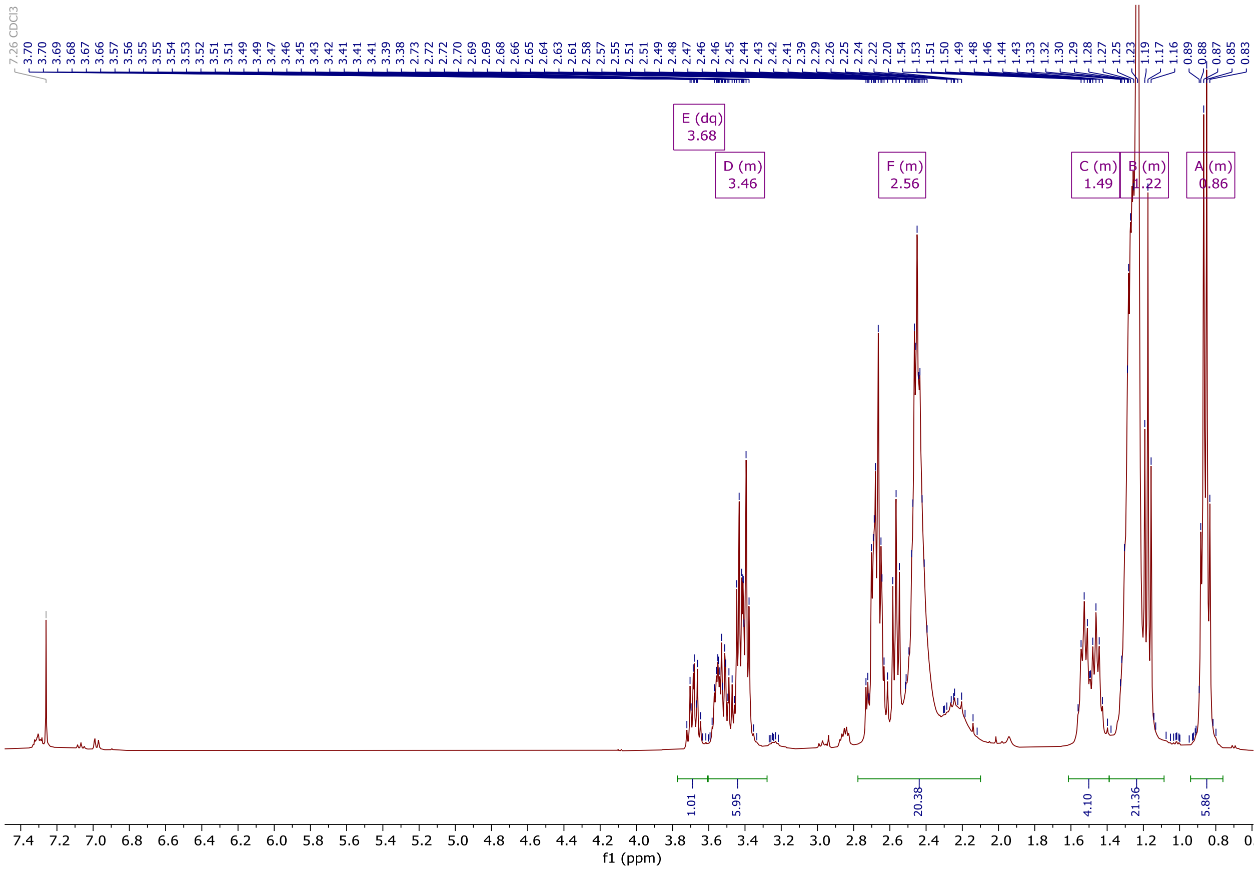

Figure S76. HRMS spectrum of compound **16f.**

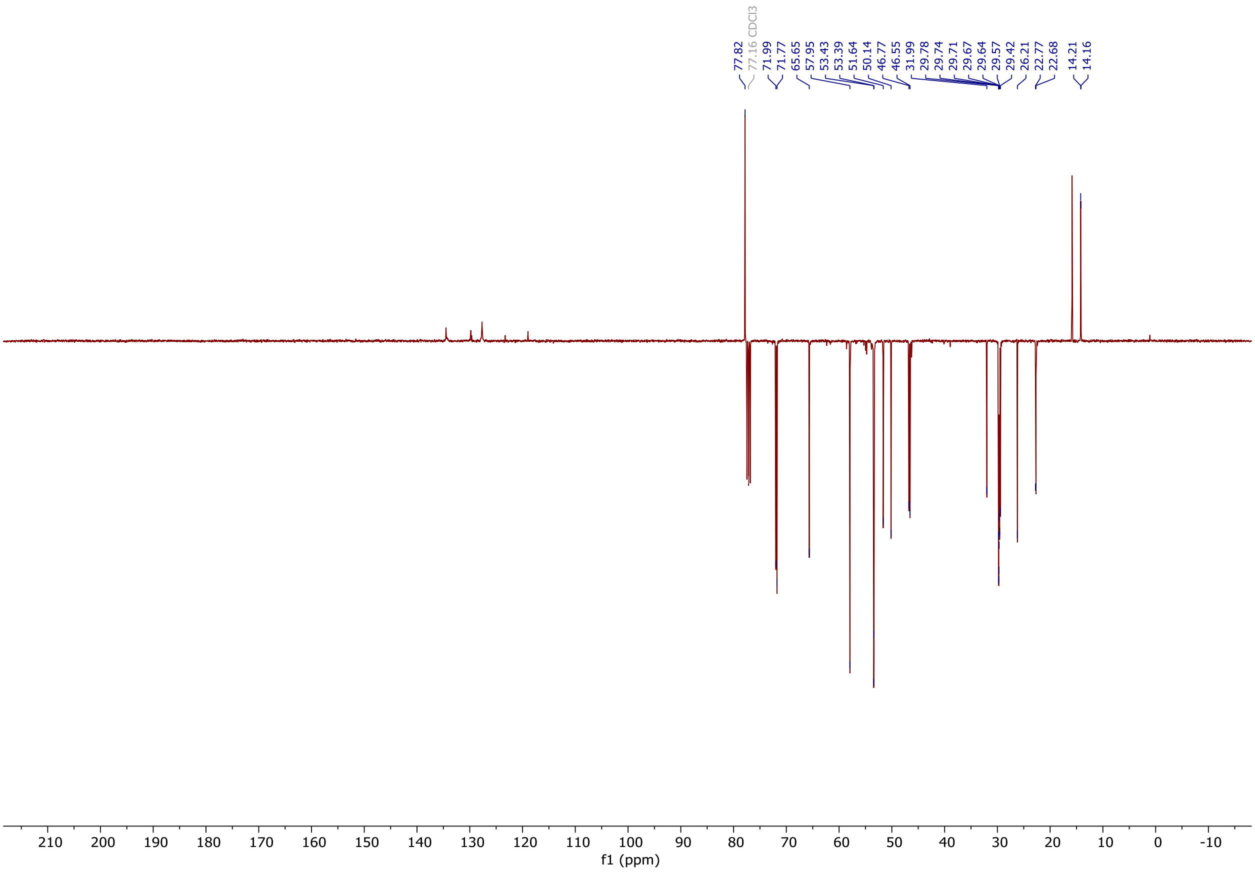

Figure S77. 13C (APT) spectrum of compound **16f.**

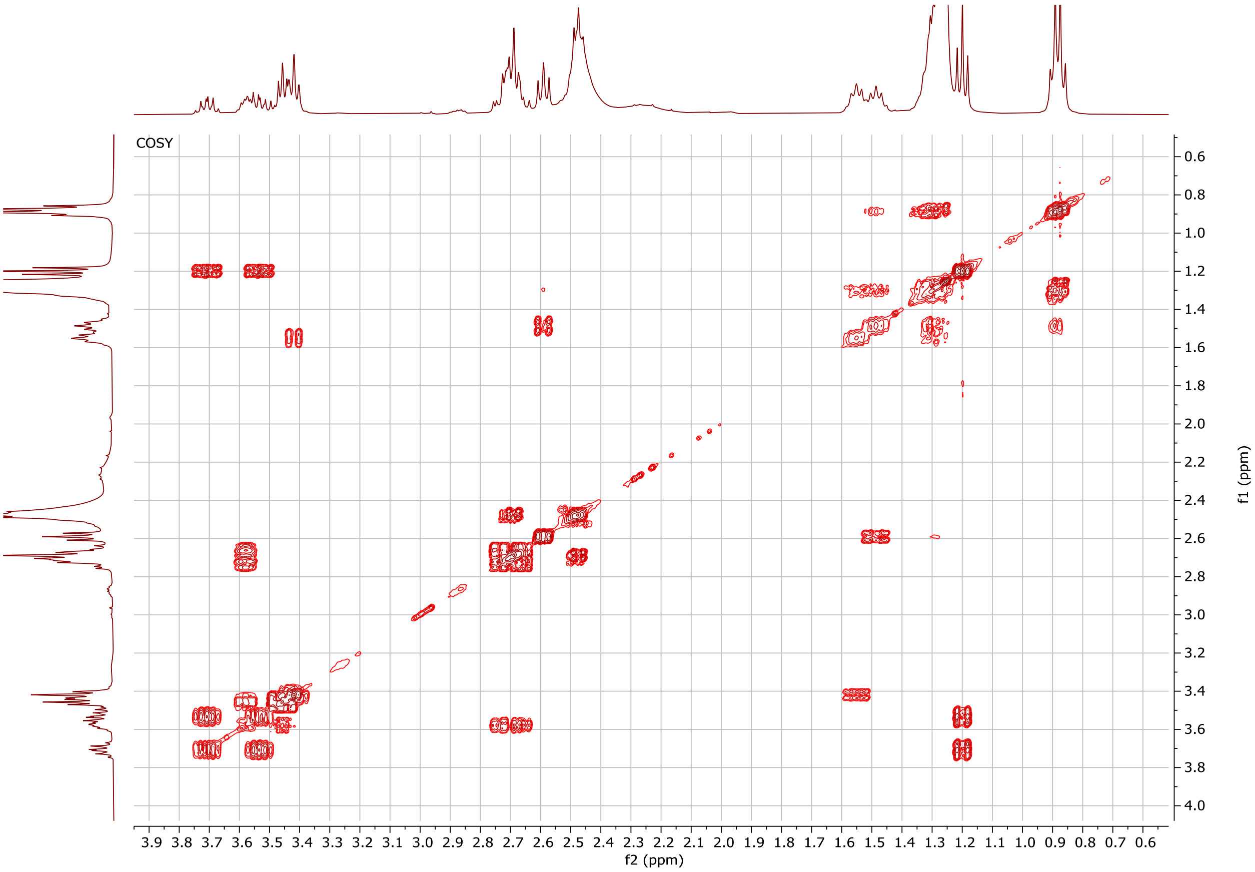

Figure S78. {^{1}H-^{1}H} COSY NMR spectrum of compound **16f.**

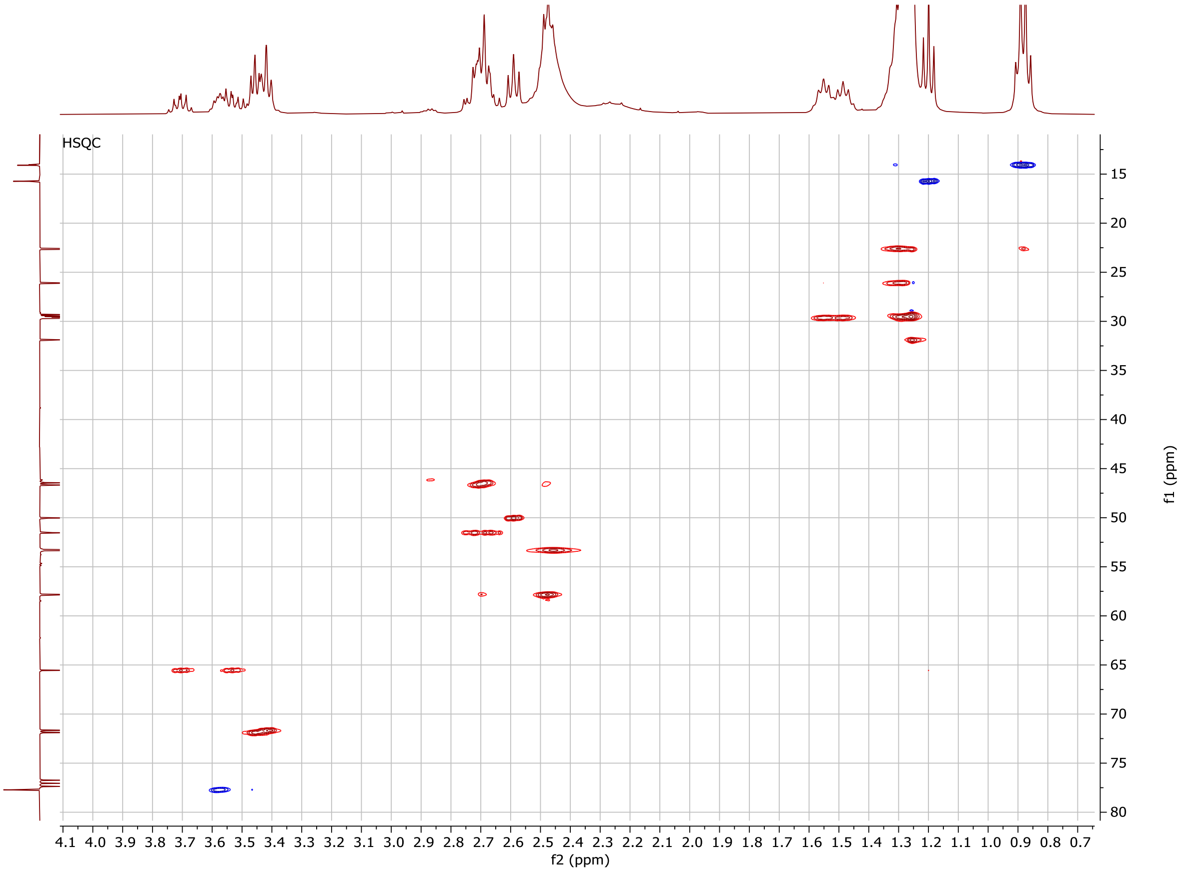

Figure S79. {¹H-¹³C} HSQC NMR spectrum of compound **16f.**

N[1]-[(β-D-glucopyranosyl)aminocarbonyl]methyl-*N*[4]-[(*N*-(*rac*-1-decyloxy-2-ethyloxyprop-3-yl)aminocarbonyl]methylpiperazin (**17**)

Yield: 89%, colorless oil. Eluent: EA-MeOH (8:2). ^{1}H NMR (600 MHz, $CDCl_3$) δ 0.87 (t, *J* = 7.0 Hz, 3H, $(CH_2)_7C\underline{H}_3$), 1.18 (t, *J* = 7.0 Hz, 3H, $OCH_2C\underline{H}_3$), 1.20 – 1.33 (m, 14H, $(C\underline{H}_2)_7CH_3$), 1.50 – 1.58 (m, 2H, $CH_2C\underline{H}_2(CH_2)_7$), 2.57 (br.s, 8H, Pip protons), 2.96 – 3.14 (m, 2H, 2 NCH_2CO), 3.14 – 3.23 (m, 1H, H-6), 3.35 – 3.63 (m, 11H, H-1, H-2, H-3, H-4, H-6, $C\underline{H}OC\underline{H}_aH_bCH_3$, $C\underline{H}_2OC\underline{H}_2$), 3.63 – 3.70 (m, 1H, $OCH_a\underline{H}_bCH_3$), 3.74 – 3.87 (m, 2H, CH_2), 7.43 (br. t, *J* = 5.9 Hz, 1H, $CH_2N\underline{H}$), 7.99 (d, *J* = 8.3 Hz, 1H, $CHN\underline{H}$). ^{13}C NMR (151 MHz, $CDCl_3$) δ 14.23, 15.86, 22.79, 26.20, 29.44, 29.60, 29.69, 29.73, 29.75, 32.01, 40.41, 40.45, 53.30, 53.59, 61.56, 65.50, 65.53, 71.37, 71.39, 72.00, 76.80, 76.85, 77.96, 79.77, 170.44, 171.92.

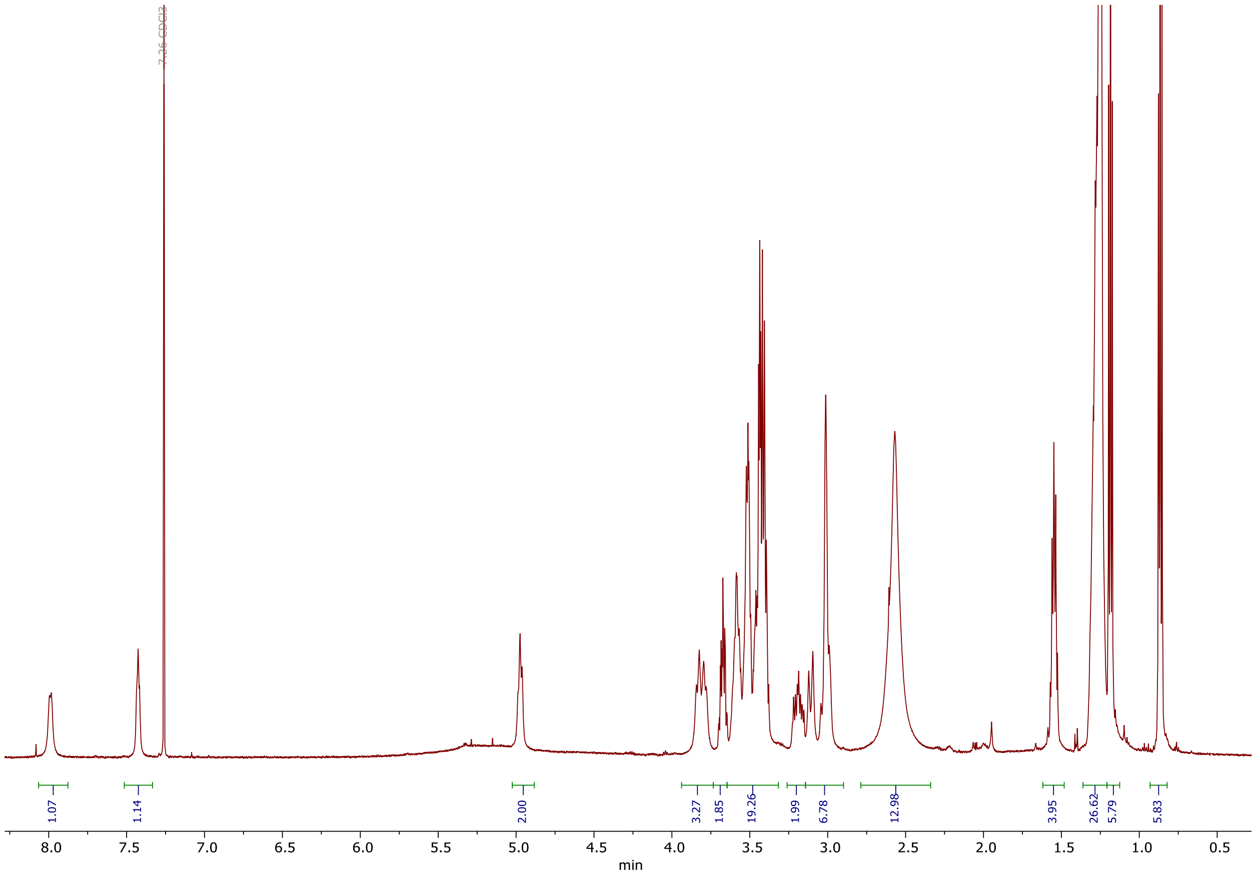

Figure S69. ^{1}H NMR spectrum of compound **17.**

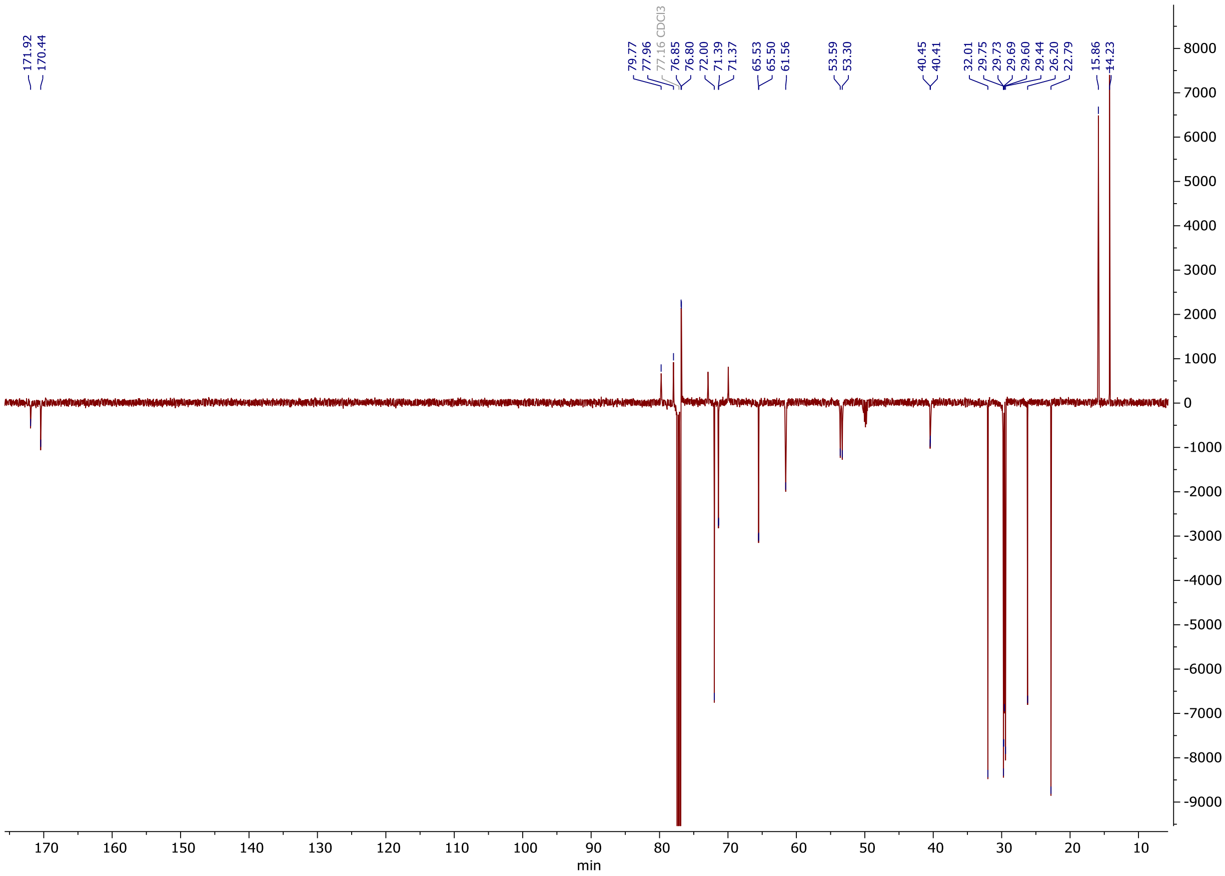

Figure S80. ^{13}C (APT) NMR spectrum of compound **17.**

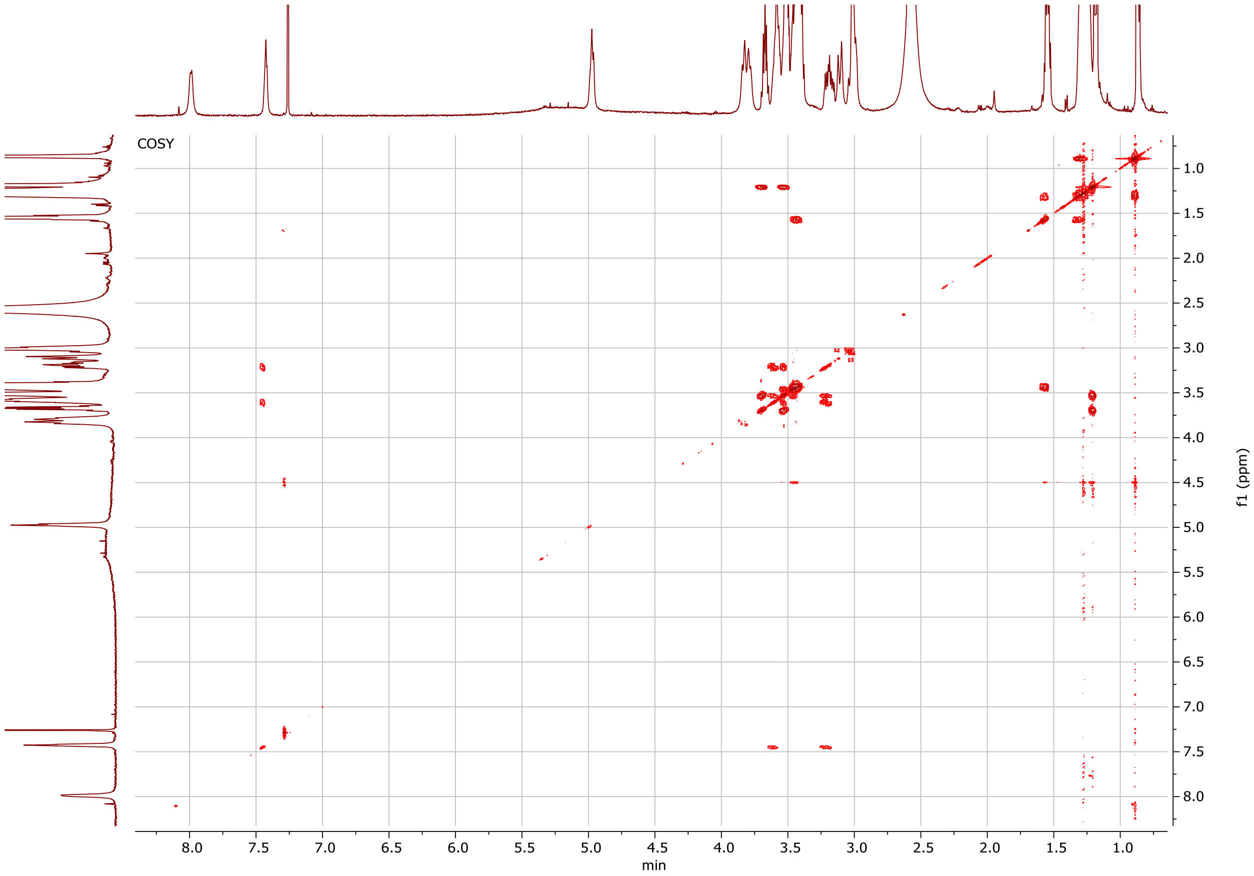

Figure S81. {^{1}H-^{1}H} COSY NMR spectrum of compound **17.**

Cell viability

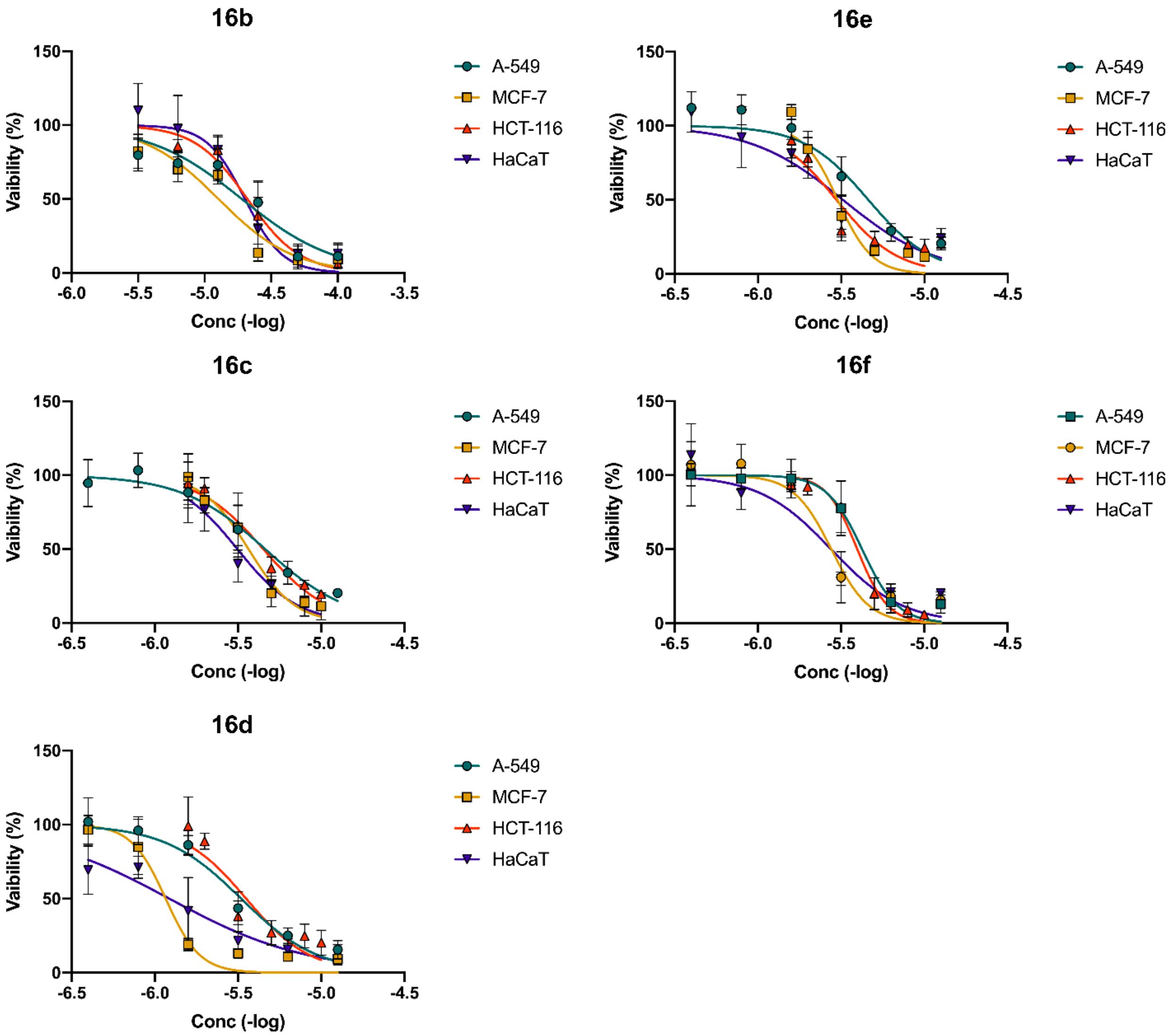

Figure S82. Cell viability of obtained compounds.
